# The habu genome reveals accelerated evolution of venom protein genes

**DOI:** 10.1038/s41598-018-28749-4

**Published:** 2018-07-26

**Authors:** Hiroki Shibata, Takahito Chijiwa, Naoko Oda-Ueda, Hitomi Nakamura, Kazuaki Yamaguchi, Shousaku Hattori, Kazumi Matsubara, Yoichi Matsuda, Akifumi Yamashita, Akiko Isomoto, Kazuki Mori, Kosuke Tashiro, Satoru Kuhara, Shinichi Yamasaki, Manabu Fujie, Hiroki Goto, Ryo Koyanagi, Takeshi Takeuchi, Yasuyuki Fukumaki, Motonori Ohno, Eiichi Shoguchi, Kanako Hisata, Noriyuki Satoh, Tomohisa Ogawa

**Affiliations:** 1 Division of Genomics, Medical Institute of Bioregulation, Kyushu University, Fukuoka, 812-8582 Japan; 2 Graduate School of Systems Life Sciences, Department of Bioscience and Biotechnology, Kyushu University, Fukuoka, 812-8581 Japan; 3 Department of Applied Life Science, Faculty of Bioscience and Biotechnology, Sojo University, Kumamoto, 860-0082 Japan; 4 Department of Biochemistry, Faculty of Pharmaceutical Sciences, Sojo University, Kumamoto, 860-0082 Japan; 5 Institute of Medical Science, University of Tokyo, Oshima-gun, Kagoshima, 894-1531 Japan; 6 Department of Information and Biological Sciences, Graduate School of Natural Sciences, Nagoya City University, Nagoya, Aichi 467-0802 Japan; 7 Department of Applied Molecular Biosciences, Graduate School of Bioagricultural Sciences, Nagoya University, Nagoya, Aichi 464-8601 Japan; 8 Department of Biomolecular Science, Graduate School of Life Sciences, Tohoku University, Sendai, Miyagi 980-8577 Japan; 9 Computational Bio-Big Data Open Innovation Laboratory, National Institute of Advanced Industrial Science and Technology, Shinjuku-ku, Tokyo, 169-0072 Japan; 10 DNA Sequencing Section, Okinawa Institute of Science and Technology Graduate University, Onna, Okinawa, 904-0495 Japan; 11 Marine Genomics Unit, Okinawa Institute of Science and Technology Graduate University, Onna, Okinawa, 904-0495 Japan

## Abstract

Evolution of novel traits is a challenging subject in biological research. Several snake lineages developed elaborate venom systems to deliver complex protein mixtures for prey capture. To understand mechanisms involved in snake venom evolution, we decoded here the ~1.4-Gb genome of a habu, *Protobothrops flavoviridis*. We identified 60 snake venom protein genes (SV) and 224 non-venom paralogs (NV), belonging to 18 gene families. Molecular phylogeny reveals early divergence of SV and NV genes, suggesting that one of the four copies generated through two rounds of whole-genome duplication was modified for use as a toxin. Among them, both SV and NV genes in four major components were extensively duplicated after their diversification, but accelerated evolution is evident exclusively in the SV genes. Both venom-related SV and NV genes are significantly enriched in microchromosomes. The present study thus provides a genetic background for evolution of snake venom composition.

## Introduction

**Figure 1:**
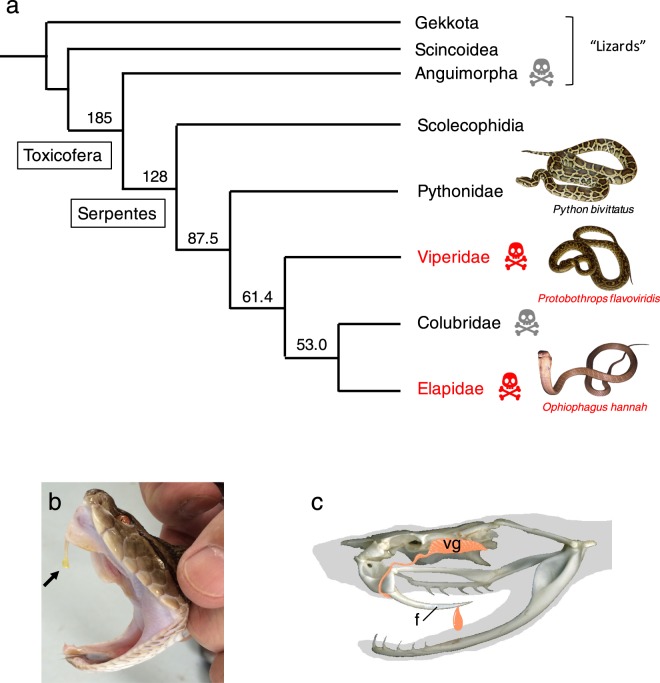
Squamate phylogeny and postorbital venom glands. (**a**) Simplified phylogenetic relationships between major clades of snakes with non-snake squamates. Numbers at nodes denote estimated divergence times in millions of years (based on Zheng and Wiens (2016)). Two entirely venomous clades, Viperidae and Elapidae are indicated with skull-and-crossbones in red. Two partially venomous clades, Anguimorpha and Clubridae are indicated with skull-and-crossbones in grey. The three clades in which draft genome sequences have been reported are shown with pictures, the Burmese Python, *Python bivattatus* [Castoe *et al*.^16^], the habu, *Protobothrops flavoviridis* (this study) and the king cobra, *Ophiophagus hannah* [Vonk *et al*.^12^]. Images of the Burmese python and the king cobra were provided by Koki Terada of the Okinawa Prefectural Institute of Health and Environment, Okinawa, Japan. (**b**) Habu venom (arrow) dripping from the fang. (**c**) A drawing of the fang (**f**) and the postorbital venom gland (vg) of *P*. *flavoviridis*.

Among squamates, the lineage leading to snakes is estimated to have diverged from that leading to Iguania and Anguimorpha more than 120 million years ago (MYa) (Fig. 1a)^1^. Snakes comprise an enormously speciose lineage (approximately 3,100 species) and show phenotypically diverse radiation. One of the key events enabling them to achieve such high diversity is the development of venoms. Extant venomous snakes are classified into three major clades, Viperidae, Colubridae, and Elapidae (Fig. 1a). Two entirely venomous clades, Viperidae and Elapidae, commonly produce highly toxic venoms with elaborate venom delivery systems. The venom gland, together with associated cranial musculature delivers the venom through specialized front fangs for prey immobilization (Fig. 1b,c). Therefore, in addition to models for evolutionary ecology and adaptation, snakes provide a unique opportunity to study venoms in association with physiological remodeling and metabolic cycling of cells^2–5^.

Snake venoms are complex protein mixtures encoded by various multi-locus gene families that function synergistically to incapacitate the prey^6–8^. So far, more than 15,000 studies have been conducted to fully characterize snake venom repertoires and to understand molecular mechanisms involved in evolution and physiological functions of snake venoms. Recent analyses of high-throughput transcriptomics have shown highly divergent venom profiles^9–11^. Despite such extensive studies, the evolutionary origins of snake venom proteins and the genetic bases of venom diversity are as yet poorly understood. One general issue is to what degree differences among species are due to differences in gene number or gene regulation. It has been proposed, for example, that major differences in venom composition between related viperid species are due to transcriptional and post-transcriptional regulatory mechanisms^6^, whereas other studies have asserted that gene duplication and divergence accounts for interspecific differences^9,11^.

However, nearly all studies of snake venom diversity have been conducted without the benefit of genome sequences that are necessary to identify orthologous genes and to untangle the contributions of gene content and regulation. Two venomous snake genomes, the king cobra (*Ophiophagus hannah*) and the five-pacer viper (*Deinagkistrodon acutus*) have been analyzed to date^12,13^. Partially due to the complexity of the composition of the snake venom proteins, the genomic basis for venom gene evolution has been left unresolved, awaiting better genome assembly and comprehensive annotations of venom protein genes, as well as highly similar non-venom paralogs. For example, to what extent did venom genes duplicate (gene diversity)? What sorts of alternative splicing (transcriptomic diversity) are involved in diversification of venom genes? Did two-rounds of whole genome duplication (2R-WGD) that occurred in the vertebrate lineage contribute to the modification of ancestral genes to produce toxic protein variants? Are duplicated gene copies scattered throughout the genome or clustered in certain chromosomal regions? If so, do macrochromosomes (MACs) or microchromosomes (MICs) host the cluster? To address these questions, we sequenced the genome of the habu, *P*. *flavoviridis*, and comprehensively analyzed genes encoding venom (SV) and paralogous, non-venom proteins (NV).

## Results

### The genome assembly

The habu (*Protobothrops flavoviridis*) genome is estimated to be approximately 1.41 Gb in size by *k*-mer analysis (Supplementary Fig. S1b). A total of 135.95 Gb of shotgun sequence reads were obtained, achieving a sequencing depth of ~96-fold (Supplementary Table S1). Contig and scaffold N50’s were 3.8 and 467 kb, respectively (Supplementary Table S2). Sequences were deposited with accession BFFQ01000001-BFFQ01084502. GC content of the *P*. *flavoviridis* genome was 39.5%, excluding gaps. The quality and completeness of the genome assembly were assessed by searching for a set of 233 core vertebrate genes using BUSCO v2^14^ implemented in gVolante^15^. Percentages of complete and partial coverages were 92.7% and 97.0%, respectively (Supplementary Table S3). The genome is estimated to contain 25,134 protein-coding gene models.

As shown in Supplementary Table S3, sizes of four extant genome assemblies are comparable, ranging 1.4 to 1.5 Gbp. Estimated numbers of protein-coding genes in *O*. *hannah* (18,445) and in *D*. *acutus* (21,194) were smaller than in *P*. *flavoviridis* (25,134) and *Python bivittatus* (25,385)^16^. Since *P*. *flavoviridis*, *O*. *hannah* and *D*. *acutus* develop venom while *Py*. *bivittatus* does not, the difference in gene numbers is probably not related to the development of venom. Detailed genic information, such as average gene length or exon-intron organization has not been published for *O*. *hannah* or *D*. *acutus*^12,13^. In *P*. *flavoviridis*, the average length of genes was 33 kb, while the average lengths of exons and introns were 219 and 3,922 bp, respectively (Supplementary Table S3). This indicates that genes of *P*. *flavoviridis* harbor longer intronic regions in general, compared to those in *Py*. *bivittatus* (Supplementary Table S3).

For transcriptomic analyses, RNA prepared from 20 samples of 18 adult tissues and organs including two venom glands was sequenced (Illumina) (Supplementary Table S4a). In addition, we prepared cDNA libraries from the venom gland, which were sequenced using the PacBio platform (Supplementary Table S4b). Transcriptomic data were used for gene annotation and examination of gene expression in the venom gland. Using BLASTP against the NCBI NR database, we carefully annotated predicted genes and identified 20,540 protein-coding genes in the habu genome, comparable to decoded genomes of other snakes (Supplementary Table S3)^12,13,16^. We prepared a genome browser for the assembly labeled as HabAm1 with gene models using the JavaScript-based Genome Browser (JBrowse) 1.11.6^17^, which is accessible at http://marinegenomics.oist.jp/habu/.

### Identification of genes encoding venom proteins

We compiled sixty keywords commonly used in description of snake venom components and their physiological functions (Supplementary Table S5). With a keyword search against 20,540 annotated gene models of HabAm1, we obtained 340 candidates potentially related to venoms. Manual curation of candidates excluded 124 as unrelated to venom, such as TNF and TNF receptor selected using the word “necrosis,” galectin selected with the word “lectin,” and anthrax toxin receptor using the word “toxin”. We also identified 24 genes encoding venom inhibitors (=endogenous anti-venoms) such as PLA2-inhibitor (gammaPLI), which were excluded from further analyses. We defined the remaining 192 as venom-related gene. Due to tandem duplications, some gene models have been resolved into multiple duplicated genes. We also utilized RNA assembly from Illumina RNA-seq reads as well as nearly full-length mRNA sequences yielded by PacBio with BLAST homology searches against the habu gene models. As a result, we identified 100 additional venom-related genes that have been unannotated in public databases. In total, we validated 284 genes as venom-related genes (SV + NV); 60 are associated with venom (SV genes) and the remaining 224 are non-venom paralogs (NV genes) (Table 1).

**Table 1:** Snake venom (SV) genes and non-venom (NV) paralogs identified in the *Protobothrops flavoviridis* genome.

Category	Family name	Venom proteins (SV)				Non-venom proteins (NV)			
		No of genes	No of transcript variants	Gene duplication	Accelerated evolution	No of genes	No of transcript variants	Gene duplication	Accelerated evolution
III	MP	11	55	+	+	57	128	+	−
	SP	11	72	+	+	34	43	+	−
	CTLP	10	11	+	+	40	54	+	−
	PLA2	9	17	+	+	31	48	+	−
II	3FTX	4	4	+	+	2	8	+	−
	APase	2	8	+	−	10	35	+	−
	CRISP	2	17	+	+	2	4	+	−
I	Vespryn	1	1	−	−	11	18	+	−
	5Nase	1	6	−	−	10	24	+	−
	DDPase	1	4	−	−	7	11	+	−
	Hyal	1	2	−	−	5	5	+	−
	NGF	1	4	−	−	3	4	+	−
	VEGF	1	6	−	−	2	6	+	−
	LAAO	1	8	−	−	2	3	+	−
	PDE	1	19	−	−	2	4	+	−
	PLB (LysoPL)	1	6	−	−	4	8	+	−
	BNP	1	1	−	−	1	1	−	−
	GPCase	1	5	−	−	1	1	−	−
Total		60	246			224	405		

Variation in SV gene transcripts were compiled from almost full-length RNA-seq data of venom gland using PacBio.

Variation in NV gene transcripts were compiled from the HabAm1 gene model with RNA-seq data of other tissues.

Venom-related genes we identified were classified into 18 families (Table 1), including metalloproteinases (MP), serine proteases (SP), C-type lectin-like proteins (CTLP), phospholipases A_2_ (PLA2), three-finger toxins (3FTX), aminopeptidases (APaseN), cysteine-rich secretory proteins (CRISP), vespryns/SPla and ryanodine receptor domain proteins (Vespryn), 5′-nucleotidases (5Nase), dipeptidyl peptidases (DDPase), hyaluronidases (Hyal), nerve growth factors or neurotrophins (NGF), vascular endothelial growth factors (VEGF), L-amino acid oxidases (LAAO), phosphodiesterases (PDE), phospholipases B (PLB), bradykinin-potentiating peptides and C-type natriuretic peptides (BNP), and glutaminyl peptide cyclotransferases (GPCase). All gene families include both SV and NV gene copies (Table 1) (see Supplementary Information for detailed characterization of each of the 18 families). The first four families have been shown to be major protein components of habu venom^9^.

### Categorization of gene families based upon the level of gene duplication

Levels of gene duplication are highly variable among the 18 gene families. Based upon the level, we categorized them into three groups. As shown in Table 1, Category I exhibits a low level of duplication and includes 11 families with a single SV gene copy, represented by Hyal (Supplementary Fig. S2a), NGF (Supplementary Fig. S2b) and LAAO (Supplementary Fig. S2c), although the number of NV counterparts varied from 1–11. Category II shows an intermediate level of duplication and includes three families with two to four SV gene copies, represented by APase, 3FTX, and CRISP (Table 1). Category III exhibits a high level of duplication and comprises four families, MP (Supplementary Fig. S3), SP (Supplementary Fig. S4), CTLP (Supplementary Fig. S5) and PLA2 (Supplementary Fig. S6). In habu venom, all four of these families display numerous SV (10–11) and NV genes (31–57) (Table 1).

To establish clear relationships of SV and NV genes within given families, molecular phylogeny was conducted in each family. These results showed that all habu SV genes of a given family clustered with SV homologs of the same family from species in the Viperidae, such as *P*. *mucrosquamatus* (Brown spotted pitviper) and *Ovophis okinavensis* (Himehabu), and in the Elapidae, such as *O*. *hannah* (King cobra). On the other hand, habu NV genes of each family are clustered with NV counterparts of other species. Several typical examples were seen in other venom protein families: Hyal (Supplementary Fig. S2a), NGF (Supplementary Fig. S2b), LAAO (Supplementary Fig. S2c), MP (Supplementary Fig. S3), SP (Supplementary Fig. S4), CTLP (Supplementary Fig. S5), and PLA2 (Supplementary Fig. S6). This suggests that the common ancestor of the Colubroidea, a superfamily including the Viperidae and Elapidae, had already developed multiple copies of SV/NV genes by duplication, and one copy had come into use in venom before the divergence of the two venomous snake families.

**Figure 2:**
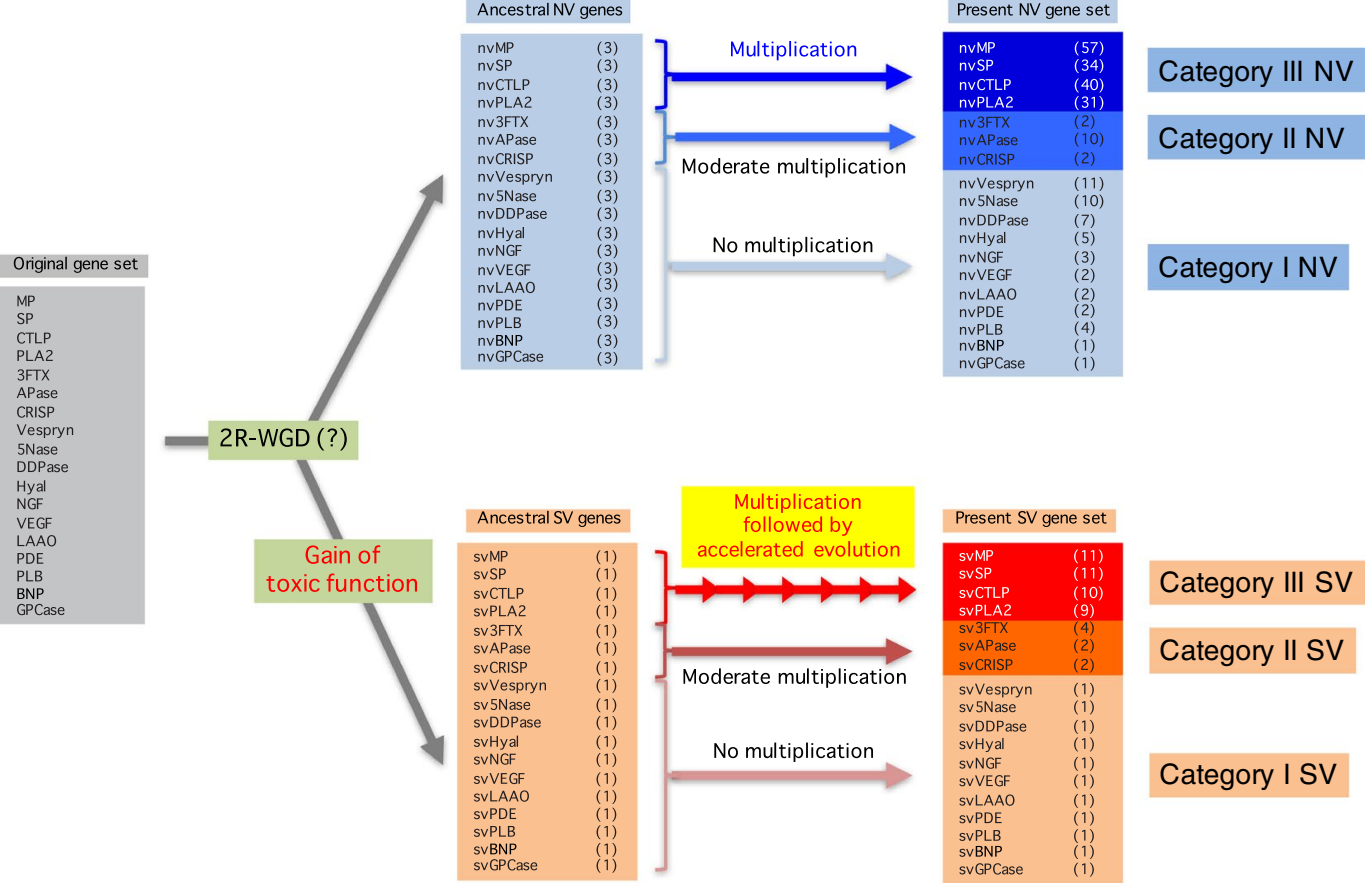
Deduced evolutionary history of venom-related gene families in the *Protobothrops flavoviridis* genome. Through two rounds of whole-genome duplication, an original set of 18 genes (shown in a grey box in the left column) became 72 (four copies each). Then, a single copy of each family was likely co-opted to develop toxic functions, resulting in one toxic copy (SV) (shown in a pale orange box in the middle column) and three non-toxic (NV) paralogs (shown in a light blue box in the middle column). Then the 18 venom protein families experienced different numbers of additional gene duplications. Eleven families (Vespryn, 5Nase, DDPase, Hyal, NGF, VEGF, LAAO, PDE, PLB, BNP and GPCase) retained more or less the original configuration, with a single SV copy and one to eleven NV copies (Category I, shown in light blue and pale orange boxes in the right column). Three families (3FTX, APase and CRISP) have experienced stochastic gene losses and gains, resulting in moderately diverse configurations with two to four SV copies and two to ten NV copies (Category II, shown in blue and orange boxes in the right column). Four families of major protein components in the venom (MP, SP, CTLP and PLA2) have experienced repeated duplication, resulting in complex configurations with 9–11 SV genes and 31–57 NV genes (Category III, shown in deep blue and red boxes in the right column). As shown in Fig. 3, SV genes in Category III also show accelerated evolution.

As described above, it is evident that gene duplication is deeply involved in diversification of genes ancestral to those encoding venom and non-venom proteins. It is generally accepted that 2R-WGD occurred during evolution of vertebrates^18^, resulting in four copies of paralogs (ohnologs). Further gene duplication to produce diversified venom proteins is likely to have originated from one of the ohnologs. In relation to this evolutionary event, several possible scenarios can be conceived (Figure S7), in which both venom and non-venom proteins are assumed to be derivatives that originated from the four ohnologs. In this molecular phylogeny, diversification of venom and non-venom protein genes occurred independently in each ohnolog lineage. The molecular phylogeny shown in Supplementary Fig. S7b shows the diversification occurred in one of the two ohnologs that were produced after the second round of WGD. On the other hand, in the phylogeny shown in Supplementary Fig. S7c, only one (in this case, ohnolog A) was duplicated to give rise to venom protein genes while in the three others, duplication did not involve the development of venom protein genes. As shown in Supplementary Figs. S2a (families of Hyal), S2b (NGF), S2c (LAAO), S3 (MP), S4 (SP), S5 (CTLP), and S6 (PLA2), all molecular phylogenetic trees of these families showed the relationship between venom proteins and non-venom proteins depicted in Supplementary Fig. S7c. Therefore, it is highly likely that one of the four copies generated by 2R-WGD was functionally modified to adopt toxic properties (SV) while the others remained non-toxic (NV) during evolution of venomous snakes (Fig. 2).

This state is more clearly maintained in Category I, that is, single SV genes with <11 NV genes. It is also likely that each family has experienced stochastic gene loss and/or gain after the divergence of SV and NV genes. In contrast, SV genes in Category III are highly expanded, with 11 copies in MP, 11 in SP, 10 in CTLP, and 9 in PLA2 (Fig. 2). Their NV counterparts are also highly multiplied with 57 copies in MP, 34 in SP, 40 in CTLP and 31 in PLA2. These four major gene families experienced massive expansions of one ohnolog following 2R-WGD in the habu genome. Category II may represent intermediate states between Categories I and III (Fig. 2).

### Extensive duplication, alternative splicing, and domain shuffling as sources for venom protein variants

Snake venom-related proteins are thought to have diversified by multiple mechanisms, such as duplication of genes, alternative splicing during mRNA expression, genic conformational changes resulting in domain-shuffling of protein products^2–5^. Footmarks of these evolutionary processes are evident in habu venom-related genes, especially in the four major protein components, MP, SP, CTLP and PLA2 of Category III.

First, as to gene duplication, both SV and NV genes of the four families are highly duplicated, resulting in 10 or 11 SV genes and >30 NV copies (Table 1). For example, the MP family contains 11 genes encoding SV proteins and 57 paralogous genes encoding NV proteins belonging to the ADAM (a disintegrin and metalloproteinase) and ADAMTS (ADAM with thrombospondin motifs) subfamilies (Fig. S3b). Among non-venom (nv) MPs, we identified 17 paralogous genes (*nvMP01* to *nvMP17*) of *ADAM*, 26 paralogous genes (*nvMP18* to *nvMP43*) of *ADAMTS* and 11 paralogous genes (*nvMP44* to *nvMP54*) of *MMP* (matrix metalloproteinase) (Supplementary Fig. S3b).

Assuming that ancestral SV or NV genes multiplied by tandem duplication, the habu genome would be expected to have retained the duplicates, creating gene clusters on the same scaffolds. We previously reported a highly complicated gene cluster of PLA2 in *P*. *flavoviridis*^19^. In the present study, we confirmed that SV and NV genes commonly form clusters in the habu genome (Supplementary Fig. S8). For example, four SV MP genes, *svMP01*, *svMP02*, *svMP03* and *svMP11* and one NV MP gene, *nvMP57* were clustered on a single scaffold, habu1_scaffold_2862 (Acc no. BFFQ01002098). Three other SV MPs, *svMP06*, *svMP07* and *svMP08* were also located on another scaffold, habu1_scaffold_14911 (Acc no. BFFQ01007560) (Supplementary Fig. S8a). Similarly, we identified three gene clusters harboring at least three svSP genes each (Supplementary Fig. S8b), and two gene clusters harboring five and nine CTLP genes (Supplementary Fig. S8c).

### Accelerated evolution of habu venom genes

Accelerated evolution has been demonstrated in toxin genes in venomous invertebrates^20^. Although data are based upon a limited number of genes, some venom genes, such as SP, PLA2, and CTLP, have also been suggested to exhibit accelerated evolution^21–26^. Using the present, complete set of SV and NV gene families in the habu genome, we analyzed molecular evolution rates by computing numbers of synonymous (*K*_S_) and non-synonymous (*K*_A_) nucleotide substitutions per site for each pair of SV and NV genes. A low ratio (*K*_A_/*K*_S_ < 1) indicates stabilizing selection, which maintains similarity between gene copies, whereas a high ratio (*K*_A_/*K*_S_ > 1) indicates diversifying selection, promoting rapid divergence of gene copies.

**Figure 3:**
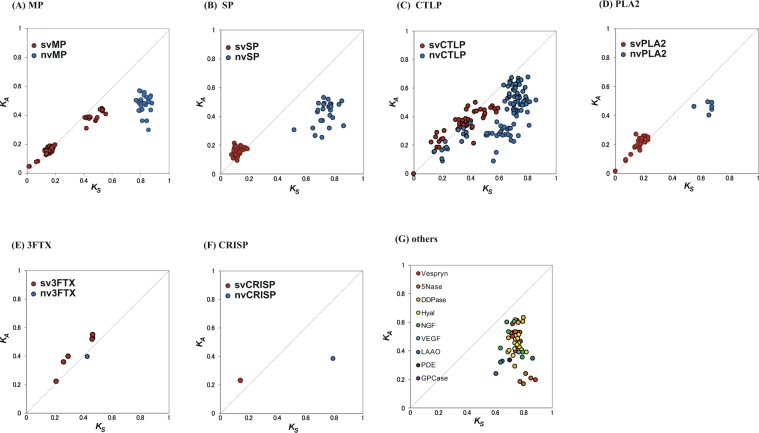
Accelerated evolution of major SV protein genes. *K*_A_ and *K*_S_ were calculated according to the Nei-Gojobori method. *K*_A_/*K*_S_ plot for MP (**A**), SP (**B**), CTLP (**c**), PLA2 (**D**), 3FTX (**E**) and CRISP (**F**), and genes in Category I (**G**). In a to f, NV and SV genes are indicated by blue and red circles, respectively.

We found that the *K*_A_/*K*_S_ ratios of both SV and NV pairs in Category I are consistently <1 (Mean +/− SE = 0.512 +/− 0.018) (Fig. 3G), suggesting rather neutral changes of nucleotides among pairs. *K*_A_/*K*_S_ ratios similar to those observed in Category I (Fig. 3G), were observed in NV genes in Category III (0.584 +/− 0.026 for nvMPs, 0.523 +/−0.024 for nvSPs, 0.304 +/− 0.066 for nvCTLPs and, 0.594 +/− 0.033 for nvPLA2s) (Fig. 3A–D). In contrast, the *K*_A_/*K*_S_ ratios were remarkably larger in SV genes in all four families in Category III (1.047 +/− 0.438 for sv MPs, 1.253 +/− 0.090 for svSPs, 0.871 +/− 0.071 for svCTLPs, and 1.093 +/− 0.062 for svPLA2s) (Fig. 3A–D). This indicates that the SV genes in Category III have been evolving in an accelerated manner, suggesting that positive selection acts on SV gene copies, causing them to diversify. Interestingly, two genes in Category II, 3FTX, and CRISP also exhibited high values of *K*_A_/*K*_S_ > 1 in SV gene copies (Fig. 3E,F), although they comprised fewer gene copies, suggesting a tendency toward accelerated evolution, as in Category III.

### Chromosomal localization of SV genes

Reptiles, including birds, have microchromosomes (MICs) in addition to the usual macrochromosomes (MACs)^27–30^. Since both GC content and recombination rate are reportedly higher in MICs than in MACs^30,31^, do MICs host preferentially the cluster of genes? To determine the chromosomal locations of venom-related genes, we utilized the synteny of *P*. *flavoviridis* chromosomes with those of the non-venomous snake, *Elaphe quadrivirgata* (Supplementary Fig. S9). Using cytological information of 143 genes available for *E*. *quadrivirgata*^28,29^, we successfully anchored 2,649 genes (10.5% of all predicted genes) corresponding to 117.3 Mb (8.3% of the genome assembly). Of the 47 venom-related (SV + NV) genes localized to specific chromosomes, 27 genes are located on MICs, indicating significant enrichment on MICs (57%), compared to all other genes anchored (32%, 837/2,602) (*p* = 0.0004 by Fisher’s exact test) (Table 2). This enrichment on MICs is even more evident when comparing only SV genes (89%, 18/19) with all other genes anchored (32%, 1,017/2,630) (*p* < 0.0001 by Fisher’s exact test). This enrichment in MICs is quite reasonable for the massive expansion and the rapid evolution in venom-related genes since both GC content and recombination rate are known to be higher in MICs than in MACs.

**Table 2:** Number of genes anchored to MICS and MACS.

Gene annotations	Chromosomal locations		Total
	MICS*	MACS**	
Venom-related genes	27	20	47
Other genes	837	1,765	2,602
Total	864	1,785	2,649

*p* = 0.0004 (Fisher’s exact test).

*Numbers of genes assigned on microchromosomes.

**Numbers of genes assigned on macrochromosomes.

## Discussion

Since several snake and anguimorpha lineages possess related venom systems, those lineages are often lumped together as a single clade, Toxicofera, including entirely non-venomous clades such as Iguania and Pythonidae^2,3^. Recent molecular phylogenetic studies support this taxonomic relationship^1,32^. Therefore, the earliest acquisition of venoms is likely to have occurred at least ~185 MYa, which corresponds to the estimated divergence time of the Toxicofera from other squamate clades^1^. Our phylogenetic analyses of venom-related genes in the habu genome revealed that the same SV copies are often shared by viperids and elapids (Supplementary Figs S2–S6). Using these sets of genes, the Toxicofera hypothesis should be examined in future studies. After neofunctionalization of the single copy to acquire toxic function, it is likely that natural selection upon the venom copies might have changed to adapt for prey capture utility, resulting in the accelerated evolution as we show here (Fig. 3).

Phylogenetic analyses also yielded important information about evolutionary history of specific gene families. For example, in the case of svMP, the clade including NaMP-like svMP (svMP11) and Jerdonitin-like svMP (svMP04) initially diverged from a ancestral svMP (Supplementary Fig. S3c), suggesting that NaMP-like svMP and Jerdonitin-like svMP may be close to a possible ancestral form of svMPs. This conjecture is corroborated by the distributions of *K*_A_/*K*_S_ plots for a pair of MPs (Fig. 3A), which can be divided into three clusters based on *K*_S_ values, high (0.6 to 0.9) for nvMPs; intermediate (0.3 to 0.5) for NaMP-like and jerdonitin-like proteins, and low (less than ~0.2) for svMPs. Since the rate of synonymous substitutions is time-constant, *K*_S_ values roughly correspond to the divergence time between copies. Since NaMP has been found in *elapid* snake venom^33^, NaMP-like svMP (*svSP11*) is hypothesized to be the earliest common ancestor of svMPs. It is presumed that svMPs have evolved from an NaMP-like ancestral svMP by gene duplication followed by domain loss and accelerated evolution^34^. Interestingly, an NaMP-like svMP gene, *svMP11*, was located on the same scaffold, habu1_scaffold_2862 (Acc no. BFFQ01002098), with three other SV MP genes, *svMP01*, *svMP02* and *svMP03*, forming a gene cluster (Supplementary Fig. S8a).

Although the geographic variation of snake venom have been known in a number of instances^35–40^, recent transcriptomic and proteomic analyses of several snake venoms have reconfirmed in detail that snake venom variation often occurs between individuals of not only interspecifically, but also intraspecifically, of which distributions are different geographic locations, diverse environment, and eating habits^7,8,41–50^, although some of the mechanisms of this variation remain unknown. In the present study, we clarified the genetic architecture of genes underlying evolution of the venom system. We found extensive duplication of venom genes (such as MP, SP, CTLP and PLA2 families of Category III). We observed various venom protein products caused by alternative splicing (e.g., MP and SP families). We also observed accelerated evolution of venom genes (MP, SP, CTLP and PLA2 families), resulting in rapid diversification of newly gained gene copies accompanied with neofunctionalization (Figs 2 and 3). The abundance of different gene copies within a gene family may contribute to expand the repertoire of effective weapons to prey capture. Gibbs and Chiucchi (2011) reported that differences in available food (mice, lizards or frogs) over a 26-month period, resulted in changes in the relative abundance of major proteins in venom such as D49-PLA2, PI-SVMP, and PIII-SVMP in Eastern Massasauga (*Sistrurus c*. *catenatus*)^45^. Therefore, rapid diversification of SV genes in the habu genome can be adaptive to provide genetic resources for the physiological response to variation and/or fluctuation in prey availability. Similarly, domain shuffling and the complex pattern of alternative splicing observed in SV genes can be adaptive by enhancing the variety of venom components able to respond to a wide variety of prey. Therefore, it is likely that evolutionary processes of SV genes are driven by natural selection to generate, maintain, and enhance the variety of venom components.

We showed here a significant enrichment of SV genes on MICs rather than on MACs (Table 2). Both GC content and recombination rate are known to be higher in MICs than in MACs^28,31^. Indeed, we observed higher GC content of scaffolds anchored on MICs (43.7%) than on MACs (37.7%). Although the higher recombination rate is likely to contribute to expansion of SV gene copies on MICs, the molecular mechanisms of accelerated evolution remained unresolved, awaiting further studies on the relationship of SV genes with *cis* and *trans* genomic contexts, such as transposable elements or microRNAs.

All of the results shown in this study have been obtained only after intensive analyses of genomic information. In other words, genome decoding is a powerful tool to understand molecular mechanisms involved in snake venom evolution. We expect that different species of venomous snakes will produce different mixtures of venom proteins employing different sets of genes; thus, further decoding of other snake genomes is essential for understanding the whole evolutionary process of snake venom systems. In this study, we focused on evolutionary process of venom-related genes; however, other systems, such as efficient venom delivery (fang) and autoprotective (endogenous inhibitors) are important in the evolution and physiology of venomous snakes. Our genome sequence data and gene models of HabAm1 are a valuable resource to clarify the genomic background of the venom delivery system and endogenous inhibitors. Furthermore, the co-evolution of these systems with venom protein genes must be clarified to understand the biology of venomous organisms.

## Material and Methods

### Biological materials

Adult *Protobothrops flavoviridis* were legally collected from Amami-Oshima Island, Kagoshima, Japan in 2011. Two adult females (Sample IDs: PFAG1 and PFAM1) were used to collect blood for genomic DNA extraction. PFAG1 was used for flow cytometry analysis and construction of whole genome shotgun libraries and mate-pair libraries. PFAM1 was also used for construction of MIC-enriched shotgun libraries. Two females (Sample IDs: PFAG1 and PFAC_A) were used for RNA extraction from multiple organs. In addition, one fetus (PFAC_B) was used for RNA extraction from fetal fibroblasts. In total, we prepared 20 specimens from 18 tissues for RNA-seq.

### Genomic DNA preparation

Genomic DNA was extracted from peripheral blood samples of PFAG1 using a QIAGEN column according to the manufacturer’s protocol. Quality and integrity of genomic DNA were examined using an Agilent 2100 Bioanalyzer (Agilent Technologies). Genomic DNA was quantified with a Qubit Fluorometer using Quant-iT assay kits (Invitrogen).

### Microchromosome (MIC)-enriched DNA

Since microchromosomes (MICs) are known to be gene-rich, we specifically prepared MIC-enriched genomic DNA for shotgun sequencing. Blood from PFAM1, diluted with RPMI medium, was separated by centrifugation (400 × g, 30 min) using lymphocyte separation solution (d = 1.077) (Nacalai tesq Co., Japan). Collected blood cells were washed with PBS, and embedded in agarose gel blocks (5 × 10^7^ cells/mL gel). Embedded cells were lysed with detergents and proteinase K using a CHEF Mammalian Genomic DNA Plug Kit (Bio-Rad Labs, Hercule, CA, USA). Agarose blocks containing genomic DNA were loaded directly into wells, and separated by PFGE using a CHEF-DRII apparatus (Bio-Rad) in 0.5% or 0.8% Mega Base Agarose (Bio-Rad) for 72 h in 1xTAE at 14 °C, 2 V/cm. *Schizosaccharomyces pombe* chromosomal DNA (CHEF DNA Size Marker (Bio-Rad)) was used as a size marker (3.5, 4.6 and 5.7 Mb). The agarose block corresponding to MIC DNA was removed and DNA was extracted from the agarose gel by treating it with thermostable agarase (Nippon Gene Co., Tokyo, Japan) at 60 °C.

### Flow cytometry

Peripheral blood cells were collected from *Protobothrops flavoviridis* (PFAG1) and *Eublepharis macularius* (Leopard gecko). Cells were stained with BD Cycletest Plus DNA Reagent Kit (BD Biosciences) according to the manufacturer’s protocol. Stained cells, as well as commercially available prestained chicken (*Gallus gallus*) erythrocyte nuclei (BD DNA QC Particles, BD Biosciences), were analyzed on a BD FACSCalibur (BD Biosciences). The genome size of *P*. *flavoviridis* was estimated by simple linear regression using the genome sizes of chicken (~1.2 Gb) and the Leopard gecko (~2.6 Gb). By this technique, the *P*. *flavoviridis* genome size was estimated to be approximately 1.8 Gb (Supplementary Fig. S1a).

### Genome sequencing

Whole genome and MIC-enriched shotgun libraries with an insert size of 600 bp were constructed for GS FLX genome analyzer using a GS FLX Titanium Rapid Library Preparation Kit (Roche) according to the manufacturers’ protocols. Whole genome shotgun libraries with insert sizes of 400 bp and 1 kb were also constructed for Illumina sequencers using a TruSeq DNA Sample Preparation Kit v2 (Illumina) according to the manufacturer’s protocols. Mate-pair libraries were constructed with four different insert sizes: 1 kb, 2 kb, 4 kb, 8 kb and 12 kb using an Illumina Mate Pair Library Prep Kit (Illumina) according to the manufacturer’s protocol. Quality and quantity of the libraries were examined with an Agilent Technologies 2100 Bioanalyzer (Agilent). Whole genome shotgun libraries were sequenced with various next-generation sequencing platforms, Roche 454 GS FLX+ (454 Life Sciences, Roche, Brandford, CT, USA), Illumina Miseq (Illumina, San Diego, CA, USA). Mate-pair libraries with different insert sizes were sequenced with an Illumina GA IIx. 135.95 Gb of sequence were obtained, resulting in approximately 96x coverage of the 1.41-Gb genome calculated by the *k*-mer analysis as described below. Sequencing reads have been deposited with accession numbers DRA006596-7 (shotgun sequencing with Roche454), DRA006598 (shotgun sequencing with MiSeq) and DRA006599 (mate-pair sequencing reads).

### *k*-mer analysis

Using the shotgun reads described below, *k*-mer analysis was conducted using Jellyfish (v2.0.0)^51^ and a custom Perl script. Total *k*-mers (*k* = 27) were 65,661,771,084. Peak coverage was found to be 43x (Supplementary Fig. S1b). The minor peak at low frequency (<10x depth) likely results from mismatches due to heterozygous SNPs. The genome size of 1.41 Gb (total length of used reads/peak coverage) closely matches the total size of assembled scaffolds (1,413,202,175 bp) (Table S1).

### Assembly and characterization

Sequencing quality was checked with a FASTX-Toolkit (http://hannonlab.cshl.edu/fastx_toolkit/). Paired-end Miseq reads were cleaned using PRINSEQ^52^. Cleaned paired-end reads were joined with fastq-join, included in the open source “ea-utils” toolkit (https://expressionanalysis.github.io/ea-utils/). Genome assembly was conducted using Platanus ver 1.2.1^53^. Then, scaffolding was done by mapping paired-end reads of 1-kb insert and mate-pair reads (2–12 kb) to contigs with Platanus. Gaps in scaffolds were then filled using GapCloser from SOAPdenovo2 package^54^. Redundancy of final scaffolds was removed with a custom perl script^55^. Genome sequences of *Protobothrops flavoviridis* have been deposited with accession numbers BFFQ01000001 - BFFQ01084502. Quality and completeness of the genome assembly were assessed by searching for the set of 233 core vertebrate genes using BUSCO^17^, implemented in gVolante^18^. Percentages of complete and partial coverages were 92.7% and 97.0%, respectively (Supplementary Table S3). We called the Habu genome assembly, HabAm1 (Habu Amami version 1), the quality of which deserves further genomic analyses, as discussed below. GC content of the *P*. *flavoviridis* genome was 39.5%, excluding gaps.

### Transcriptome analyses by Illumina

For transcriptomic analyses, 20 samples of 18 adult tissues and organs including one fetal tissue were used (Supplementary Table S4a). We extracted total RNA using a standard TRIzol protocol procedure (Thermo Fisher Scientific), and prepared cDNA libraries using an NEBNext® Ultra™ Directional RNA Library Prep Kit for Illumina (New England Biolabs). RNA quality was checked with an Agilent Technologies 2100 Bioanalyzer using an Agilent RNA 6000 Nano Kit. Sequencing was performed using an Illumina Hiseq2500. *De novo* assembly of whole RNA sequence reads was performed using a de Bruijn graph-based program, Trinity^56,57^. Assembled transcripts were annotated with BLASTX against UniProt. All Illumina reads are available from DRA under accession no. DRA006600.

### Transcriptomic analyses with PacBio reads

We prepared cDNA libraries from the venom gland for PacBio sequencing using the manufacturer’s protocol with a SMARTer Pico PCR cDNA Synthesis Kit (TAKARA Clontech) and SMRTbell Template Preparation Kit 1.0 (PacBio). We enriched longer cDNAs with a SageELF system (Sage Science, Inc). Sequencing was performed on a PacBio RS II, yielding a total of 179,143,509 reads with an average read length of 2,300 bp (Supplementary Table S4b). Most of these reads are long enough to be full-length transcripts and they were directly annotated with BLASTX against UniProt. PacBio reads are available from DRA under accession no. DRA006601.

### Gene modeling

We mapped assembled transcripts against the genome assembly, HabAm1 using BLAT^58^ and PASA^59^. We extracted exon/intron junction sequences to create a hint file for *ab initio* gene prediction. Gene models were predicted with AUGUSTUS^60^ using the hint file. We identified 25,134 protein-coding genes from HabAm1. By homology search using BLASTP against the NCBI NR database, we identified 20,540 genes with functional annotations.

### Molecular evolutionary analysis

Amino acid sequences were aligned using the MAFFT multiple alignment program (http://mafft.cbrc.jp/alignment/server/)^61^ and/or ClustalW (http://clustalw.ddbj.nig.ac.jp)^62^. Due to the highly complicated structure of many SV and NV genes, all alignments were manually curated. For pairwise comparisons of nucleotide sequences of SV genes as well as NV genes, numbers of nucleotide substitutions per synonymous site (*K*_S_) and per non-synonymous site (*K*_A_) for protein-coding regions were computed according to the Nei-Gojobori method^63^ using Sqdif Plot online (http://www.gen-info.osaka-u.ac.jp/~uhmin/study/sqdifPlot/index.html). All phylogenetic trees were reconstructed with the maximum likelihood method using IQ-TREE (http://www.iqtree.org)^64^. The optimal evolutionary model for each phylogenetic tree was selected using ModelFinder^65^ implemented in IQ-TREE (Supplementary Table S6).

### Chromosomal localization

Cytological information for 143 genes was available for *Elaphe quadrivirgata*^28,29^. We utilized the synteny of *Protobothrops* chromosomes with *Elaphe* chromosomes to anchor the genome scaffolds. We successfully anchored 2,639 genes (10.5% of all predicted genes) totaling 117.3 Mb (8.3% of the total draft genome).

### Data Availability

Genome and transcriptome sequence data can be accessed from NCBI and DDBJ. Raw genome sequence data can be accessed in BioSamples SAMD00115727 (DRA006596 - DRA006599). Accession numbers for scaffolds are BFFQ01000001 - BFFQ01084502 (84,502 entries). The accession number of the transcriptomic data in the NCBI Sequence Read Archive is DRA006600 (HiSeq) and DRA006601 (PacBio). We prepared a genome browser for the assembly, HabAm1 with gene models, using the JavaScript-based Genome Browser (JBrowse) 1.11.6^17^, which is available at: http://marinegenomics.oist.jp/habu/.

## Electronic supplementary material

Supplementary Information

Supplementary Table S1

Supplementary Table S2

Supplementary Table S3

Supplementary Table S4

Supplementary Table S5

Supplementary Table S6

Supplementary Table S7
