# Supplementary material for: The habu genome reveals accelerated evolution of venom protein genes
Source: Sci Rep. 2018 Jul 26;8:11300. doi: 10.1038/s41598-018-28749-4 (PMC6062510; doi:10.1038/s41598-018-28749-4)

SUPPLEMENTARY INFORMATION

The habu genome reveals accelerated evolution of venom protein genes.

Hiroki Shibata[a, b, 1], Takahito Chijiwa[c], Naoko Oda-Ueda[d], Hitomi Nakamura[d], Kazuaki Yamaguchi[c], Shousaku Hattori[e], Kazumi Matsubara[f], Yoichi Matsuda[g], Akifumi Yamashita[h], Akiko Isomoto[a, b], Kazuki Mori[b, i], Kosuke Tashiro[b], Satoru Kuhara[b], Shinichi Yamasak[j], Manabu Fujie[j], Hiroki Goto[j], Ryo Koyanagi[j], Takeshi Takeuchi[k], Yasuyuki Fukumaki[a], Motonori Ohno[c], Eiichi Shoguchi[k], Kanako Hisata[k], Noriyuki Satoh[k, 1] and Tomohisa Ogawa[h, 1]

[a]Division of Genomics, Medical Institute of Bioregulation, Kyushu University, Fukuoka 812-8582, Japan

[b]Graduate School of Systems Life Sciences, Department of Bioscience and Biotechnology, Kyushu University, Fukuoka 812-8581, Japan

[c]Department of Applied Life Science, Faculty of Bioscience and Biotechnology, Sojo University, Kumamoto 860-0082, Japan

[d]Department of Biochemistry, Faculty of Pharmaceutical Sciences, Sojo University, Kumamoto 860-0082, Japan

[e]Institute of Medical Science, University of Tokyo, Oshima-gun, Kagoshima 894-1531, Japan

[f]Department of Information and Biological Sciences, Graduate School of Natural Sciences, Nagoya City University, Nagoya, Aichi 467-0802, Japan

[g]Department of Applied Molecular Biosciences, Graduate School of Bioagricultural Sciences, Nagoya University, Nagoya, Aichi 464-8601, Japan

[h]Department of Biomolecular Science, Graduate School of Life Sciences, Tohoku University, Sendai, Miyagi 980-8577, Japan

[i]Computational Bio-Big Data Open Innovation Laboratory, National Institute of Advanced Industrial Science and Technology, Shinjuku-ku, Tokyo 169-0072, Japan.

[l]DNA Sequencing Section, Okinawa Institute of Science and Technology Graduate University, Onna, Okinawa 904-0495, Japan

[k]Marine Genomics Unit, Okinawa Institute of Science and Technology Graduate University, Onna, Okinawa 904-0495, Japan

[1]Correspondence and requests for materials should be addressed to H.S. (email: hshibata@gen.kyushu-u.ac.jp), N.S. (e-mail: norisky@oist.jp), and T.O. (email: tomohisa.ogawa.c3@tohoku.ac.jp)

Characterization of the habu venom genes

Followings are descriptions of each of the 18 families.

(a) Metalloproteinases (MP)

Metalloproteinases (MPs) are one of the major constituents of snake venoms and key toxins involved in venom-induced pathogenesis such as hemorrhage, fibrinolysis and apoptosis. Snake venom MPs (svMPs) are classified into four groups (P-I to P-IV) according to their domain architecture and size: P-I MPs possess a metalloproteinase domain only and are largely hemorrhagic; P-II contain metalloproteinase and disintegrin domains and are larger; P-III have metalloproteinase, disintegrin, and cysteine-rich domains; and P-IV have lectin-like domain(s) linked by disulfide bonds to a P-III structure[1]. The structural complexity of P-III MPs yields a great diversity of their functions. P-II, P-III, and P-IV groups belong to the 'disintegrin and metalloproteinase' (ADAM) family.

A thorough examination of *P. flavoviridis* gene models and transcriptomes identified at least 11 *svMP* genes (*svMP01* to *svMP11*) (Supplementary Fig. S3a; Table 1) and 55 transcripts that are expressed in the venom gland (Supplementary Fig. S3a; Table 1). *svMP01*, *svMP02*, *svMP03* and *svMP11* (Gene model ID: habu1_s2862_g10314a/b/c/d) were located tandem on the same scaffold (habu1_scaffold_2862, Acc no. BFFQ01002098) (Supplementary Fig. S8a). They encoded a vascular apoptosis-inducing protein HV1, flavorase, a VMP III-like protease, and NaMP-like protease, respectively (Supplementary Fig. S3a). Three genes, *svMP06* (Gene model ID:

habu1_s14911_g21429), *svMP07* (g21430), and *svMP08* (g21431), which encode H2 metalloproteinase, HR2a, and flavoridin, respectively, were also located on the same scaffold, habu1_scaffold_14911 (Acc no. BFFQ01007560) (Supplementary Fig. S3a; Supplementary Fig. S8a). Since the two scaffolds form a super-scaffold, it is highly likely that *svMPs* (*svMP01-03, 11* and *svMP06-08*) are located tandem in close proximity to each other (Supplementary Fig. S8a). In addition, *svMP04* genes (Gene model ID: habu1_s3258_g11210) and *svMP05* (g11211) encoded a jerdonitin-like protease, the major hemorrhage factor, HR1a, and a jerdonitin-like protease, respectively, and they were all located on habu1_scaffold_3258 (Acc no. BFFQ01002364) (Supplementary Fig. S3a). *svMP09* and *svMP10* (Gene model ID: habu1_s399953_g24864a/b) encoded HR1b and Mt-b/elegantin-like protease, respectively, and were located on habu1_scaffold_399953 (Acc no. BFFQ01081176) (Supplementary Fig. S3a). Numbers of exons have also become highly variable in this gene family, ranging from 13 to 17, of which exon 11 contains a catalytic zinc binding motif, HEXGHNLGXXHD (Supplementary Fig. S10).

Furthermore, among non-venom (nv) MPs, we identified 17 paralogous genes (*nvMP01* to *nvMP17*) of *ADAM* (a disintegrin and metalloproteinase) and 26 paralogous genes (*nvMP18* to *nvMP43*) of *ADAMTS* (ADAM with thrombospondin motifs) and 11 paralogous genes (*nvMP44* to *nvMP54*) of *MMP* (matrix metalloproteinase) (Supplementary Fig. S3b). ADAM and ADAMTS include trans-membrane and secreted proteins with functions in cell adhesion and proteolytic processing of the ectodomain of

cell-surface receptors and signaling proteins as key contributing molecules to various physiological functions including cell adhesion, fertilization, migration, proteolysis and signaling[2-5] in addition to other MPs such as MMP (*nvMP44* to *nvMP54*). Predicted genes encoding nvADAMs and nvADAMTS were located on different scaffolds than svMPs, except for nvMP57 located on habu1_s2862, which contains *svMP01*, *svMP02*, *svMP03* and *svMP11* (Supplementary Fig. 3b, Supplementary Fig. 8a).

Phylogenetic analysis shows that habu SV and NV metalloproteinase genes are distinctly clustered into SV and NV homologs such as ADAM, ADAMTS, and MMP from different venomous snakes and other reptile except for ADAMTS9 (Supplementary Figs. S3c, S3d and S3e). Phylogenetic tree of MP also shows that svMPs has been derived from the ancestor of ADAM clade including ADAM8, 9, 12, 15, 19, 21, 32, and 33 (Supplementary Fig. S3c). Moreover, it was found that the apparent clade including NaMP-like svMP (svMP11) has initially diverged from a common ancestor of *P. flavoviridis* svMPs (Supplementary Fig. S3d).

(b) Serine proteases (SP)

Serine proteases (SPs) are also major constituents of venom proteins. They act on various macromolecular substrates of blood coagulation, fibrinolytic, and kallikrein-kinin systems, and on platelets to cause hemostatic defects[6]. Although svSPs belong to the chymotrypsin subfamily (peptidase subfamily S1A), they show distinct substrate specificities toward macromolecules, compared the relatively non-specific activity of trypsin or chymotrypsin.

The *P. flavoviridis* genome contains at least 11 *svSP* genes (*svSP01* to *svSP11*) (Supplementary Figs. S4a and S11) with complete sequences. *svSP01* (*TLf1*) was the most abundant *svSP* transcript in the venom gland corresponds to an acidic protein, known as flavoxobin[7,8] and habutobin[9]. It encodes a weakly thrombin-like enzyme of 242 amino acids that specifically releases fibrinopeptide A from fibrinogen[7]. Although no information is available with regard to possible kallikrein-like activity, it was shown that flavoxobin acts as a heterologous C3-convertase that selectively releases human C3b and C3a[10]. *svSP02* (*TLf2*) is also major *svSP* transcript that encodes a basic thrombin-like enzyme (pI >10) (Supplementary Fig. S4a).

More recently, we found that TLf2 enhances the myonecrotic activity of Lys49-PLA2, although TLf2 was hydrolytically inactive due to replacement of its catalytic residue His57 with Arg (Ogawa et al., in preparation). From the *P. flavoviridis* venom, a kinin-releasing enzyme, flavorase, and thrombin-like enzyme possessing fibrinopeptide A- and B-releasing activity, flavoviridiobin, have been reported[11, 12]. However, since no sequence data are available, we could not identify them among transcripts and genome sequences of our analyses. From the sequence homologies of *P. flavoviridis* svSPs with other snake svSPs using phylogenetic tree analysis (Supplementary Fig. S4c), svSP03, svSP04, and svSP05 correspond to beta fibrinogenase, catroxase-like kinin-releasing enzyme, and flavorase, respectively, which have kinin-releasing activity similar to KN-BJ from *Bothrops jararaca* venom[13]. Several of the genes were located tandem in the certain scaffolds, that is, *svSP06* (Gene ID: habu1_s4106_g13431a), *svSP07* (g13431b) and *svSP10* (g13432) were located on scaffold_4106 (Acc no.

BFFQ01002946) (Supplementary Fig. S8b). *svSP04* (habu1_s6789_g17480), *svSP05* (g17481a), and *svSP08* (g17481b) were located on the scaffold_4106 (Acc no. BFFQ01002946), while *svSP09* (habu1_s7597_g18190c), svSP01 (g18190a), and svSP03 (g18190b) were located on the scaffold 7597 (Acc no. BFFQ01004842) (Supplementary Fig. S8b).

Based on the similarity of enzymatic activities and the conservation of primary and tertiary structures of SPs, it is believed that svSPs evolved from an ancestral kallikrein-like SP. *svSP* genes consist of 6 exons except for *svSP06*, which contains additional two exons (exons 7 and 8) including 3' non-coding region (Supplementary Fig. S11). *svSP* possess the same exon-intron junctions as coding regions of mammalian *kallikrein* (*KLK*)[14] and trypsin gene rather than other SPs such as elastase (Supplementary Fig. S11b). In addition to sv-related *SP* genes, we found at least 34 SP-family genes with non-venom related functions (Supplementary Fig. S4b).

Phylogenetic analysis shows that habu SV and NV serine proteinase genes are distinctly clustered with SV and NV homologs from different venomous snakes and other reptile. Furthermore, phylogenetic tree of SP also shows that svSPs derived from the trypsin-like SPs (Supplementary Fig. S4c).

(c) C-type lectin-like proteins (or snaclecs) / Galactose binding lectins (CTLP)

C-type lectins (CTLPs) are one of the major animal lectin families, members of which bind in Ca^{2+}-dependent fashion to carbohydrates. In general, they adopt multidomain structures and contain one or more highly conserved carbohydrate recognition domain

(CRD) consisting of 115–130 amino acids with a unique α/β topology. To date, the CTL domain superfamily has been classified into 17 groups[15-18] first discovered lectin activity (agglutinating activity of erythrocytes and leukocytes) in snake venoms, and the first isolation and characterization were reported by Gartner et al.[19].

To date, primary structures of 15 kinds of snake venom galactose-binding lectins (svGBL) have been determined, and all svGBLs are C-type lectins[20]. Furthermore, C-type lectin-like proteins (CTLP) lacking carbohydrate-binding activity have been isolated from snake venom and characterized as heterodimers, which were dimerized by unique domain swapping between alpha and beta chains, resulting in a conformational change in the central loop with a new concave surface[21,22]. Dimeric svCTLPs showed several pharmacological activities such as anticoagulant, platelet aggregations (agonistic) and their inhibition (antagonistic) via specific binding to coagulant factors IX and X, von Willebrand factor, and integrins on the platelet such as GPIa/IIa, GPIb, GPVI.

The present study identified 10 genes encoding svCTLPs (Supplementary Fig. S5a) and 39 genes for non-venomous (nv) CTLs (Supplementary Fig. S5b). Molecular phylogeny shows that the ten svCTLP genes are subdivided into three groups, lectin-type (*svCTLP08* and *svCTLP10*), B-chain type (*svCTLP05*, *06* and *07*), and A-chain type (*svCTLP01*, *02*, *03*, *04* and *09*) (Supplementary Fig. S5c). The genes encoding svCTLPs were composed of six exons with conserved exon-intron junctions except for *svCTLP08*, which lacks exons 1 - 3, resulting in four exons (Supplementary Fig. S5a). *svCTLP01*, *svCTLP02*, *svCTLP05* and *svCTLP09* encode factor XI/X binding protein alpha,

flavocetin A alpha, factor XI/X binding protein beta, and new factor XI/X binding protein alpha-like with 83% identity, respectively, although they were not assigned gene model ID numbers (Supplementary Fig. S5a). Interestingly, four genes, *svCTLP03* (Gene model ID: habu1_s10061_g19810_c), *svCTLP04* (g19810_a), *svCTLP06* (g19809), and *svCTLP07* (g19810_b), which encode stejaggregin-A like alpha, rhodocetin/EmEMS 16-like alpha, flavocetin A beta, and rhodocetin/EmEMS 16-like beta, respectively, were located tandemly on habu1_scaffold_10061 (Acc no. BFFQ01005768) (Supplementary Fig. S8c).

Non-venom CTL genes encoding C-type lectins, *nvCTLP01* (Gene model ID: habu1_s10061_g19804), *nvCTLP02* (g19805), *nvCTLP03* (g19806), *nvCTLP04* (g19807), and *nvCTLP05* (g19808) (Supplementary Fig. S5b), were located on the same scaffold, habu1_scaffold_10061 (Acc no. BFFQ01005768) (Supplementary Fig. S8c). On the other hand, *svCTLP08* (Gene model ID: habu1_s3168_g10977) and *svCTLP10* (g10975), which encoded venom C-type lectins, were located on habu1_scaffold_3168 (Acc no. BFFQ01002306). Non-venom CTL genes, *nvCTLP12* (Gene model ID: habu1_s3168_g10976), *nvCTLP13* (g10978), and *nvCTLP14*, and (g10979) (Supplementary Fig. S5b) were also located on habu1_scaffold_3168 (Acc no. BFFQ01002306) (Supplementary Fig. S8c).

(d) Phospholipases A_2 (PLA2)

Phospholipase A_2 (PLA_2, EC 3.1.1.4) catalyzes hydrolysis of 2-acyl ester bonds of 3-*sn*-phosphoglycerides in the presence of Ca^{2+}, to liberate 3-*sn*-lysophosphoglycerides

and fatty acids[23]. Snake venoms PLA_2s are major toxins and are classified into groups I and II based on disulfide bonds[24]. Group-I PLA_2s are found in elapid venoms while group II are in viperid venoms. Group-II PLA_2s are further divided into two subgroups, [Asp49] PLA_2 forms and [Lys49] PLA_2 forms[25]. It has been reported that they share the same scaffold[26-28]. Numerous studies have shown the presence of multiple PLA_2 genes in habu venoms[29-34].

The *P. flavoviridis* genome contains ten types of group II venom PLA_2 genes (*svPLA201a, b* to *svPLA209*) (Supplementary Fig. 6a). In addition, we also confirmed that four PLA_2 transcripts, including a hemolytic [Asp49] PLA_2 (*svPLA201*) [Accession #: D10070.1, D10720.1, D10722.1, AB778558.1, AB072174.1, AB778559.1], edema-inducing basic [Asp49]PLA2 (*svPLA202*), a weak neurotoxin PLA-N (*svPLA203*) [Accession #: AB848131], and [Lys49]PLA2 myotoxins (*svPLA204* to *svPLA206*)) (BPI, BPII and BPIII) [Accession #: D10718.1, D10719.1, AB470470.1], were expressed in the venom gland (Supplementary Fig. S6a).

In addition, the *P. flavoviridis* genome contains at least 31 genes encoding non-venom PLA2s (Supplementary Fig. S6b). These include type I PLA2s such as *nvPLA201* and *nvPLA202* (habu1_s9792_g19655 and habu1_s9792_g19656, respectively) and type II PLA2, *nvPLA203* (habu1_s4562_g14470) (Supplementary Fig. S6b). Molecular phylogeny of PLA2 showes that svPLA2 genes have been evolved from ansestoral gene of GIIE gene, and then diversified into multiple gene types with several toxic activities such as myotoxic, hemolytic, edema-inducing and neurotoxic (Supplementary Fig. S6c).

(e) Three-finger toxin (3FTX)

Three-finger toxins (3FTXs) are non-enzymatic neurotoxins composed of about 60-70 amino acid residues. They are major components in the venoms of elapids (cobras, kraits and mambas), hydrophiids (sea snakes) and colubrids, but extremely minor components in viperid snakes[42,43]. Although the gross structures of 3FTXs are conserved, they have diverse pharmacologies such as L-type calcium channel inhibition, acetylcholinesterase inhibition, and muscarinic and nicotinic acetylcholine receptor antagonism[44,45]. 3FTXs were considered specific to elapid venoms until they were shown to be present in *Sistrurus catenatus edwardsii* venom (0.83% abundance)[37,46] and a venom gland transcriptome of *P. flavoviridis*[21].

We identified four genes encoding 3FTXs in the *P. flavoviridis* genome (*3FTX-01, 3FTX-02, 3FTX-03* and *3FTX-04;* Gene models habu1_s4579_g14476, _g14477, _g14478, _g14475, respectively) (Supplementary Fig. S12a). These are located tandemly on the same scaffold, habu1_scaffold_4579 (Acc no. BFFQ01003259). From RNA-seq data of *P. flavoviridis* tissues, these *3FTX* genes generate single transcripts, respectively (Supplementary Fig. S12a). *3FTX-01* and *3FTX-02* were expressed in venom gland. Three *3FTXs, 3FTX-01*, *02* and *04*, have highly conserved Cys residues.

Furthermore, two non-venom 3FTX genes, *3FTX-05* (habu1_s138_g00722) and *3FTX-06* (habu1_s138_g00721), encoding UPAR-Ly6 and CD59, respectively, were identified on the same scaffold, habu1_scaffold_138 (Acc no. BFFQ01000122) (Supplementary Fig. S12a). Seven transcript variants were generated from *3FTX-05*,

although other *3FTX* genes generate single transcript. Molecular phylogeny shows that toxic 3FTX proteins form a clade different from non-toxic protein clade (Supplementary Fig. S12b).

(f) Aminopeptidase (APaseN)

Aminopeptidases (EC 3.4.11) remove one or more specific N-terminal residues from target proteins or peptides. For example, aminopeptidase L (APL: leucyl aminopeptidase) removes an N-terminal leucine residue. Aminopeptidase A (APA) and aminopeptidase B (APB) remove an acidic N-terminal residue and a basic N-terminal residue, respectively. Aminopeptidase N (APN) removes neutral N-terminal residues, typically alanine. To date, several aminopeptidase activities have been detected in venoms from elapids and vipers, and the cDNA sequence of APA from *Gloydius blomhoffii brevicaudus* snake has been determined[47].

In this study, we identified two *svAPases* genes and ten *nvAPases* genes (Supplementary Fig. S13a). *svAPases* genes include *APase01* (habu1_s1390_g05001) and *APase 03* (habu1_s3769_g12636), which encode APN and APA, respectively. They reside in independent scaffolds. On the other hand, ten *nvAPases* genes include aminopeptidase-like (*APNPEPL*) (habu1_s5205_g15384), *APB* (habu1_s89848_g19021), *APO* (habu1_s4166_g13605), *APQ* (habu1_s3446_g11762), *APD* (habu1_s133_g00676), two *APMs* (habu1_s7599_g18195 and habu1_s9114_g19166), and three *XPAPs* (habu1_s4753_g14695, habu1_s4159_g13556, and habu1_s1233_g04248) (Supplementary Fig. S13a).

Molecular phylogeny shows that APN and APA form a clade, and this SV-related clade has affinity with a clade including APases O, B and Q (Supplementary Fig. S13b).

(g) Cysteine-rich secretory proteins (CRISP)

Cysteine-rich secretory proteins (CRISP) are glycoproteins implicated in the mammalian male reproductive functions spanning haploid germ cell development, epididymal maturation, capacitation, motility, and the actual processes of fertilization[48]. The first discovered CRISP, acidic epididymis glycoprotein (AEG, also known as protein D/E or CRISP-1), is an androgen-responsible secretory protein involved in gamete fusion. Two other mammalian CRISPs, testis-specific CRISP-2 (TPX-1) and salivary gland-specific CRISP-3 (28 kDa specific granule protein, SGP28) have been isolated and characterized.

CRISP-family proteins belonging to CRISP-3 have also been found in various snake venoms[49-52] and the Mexican beaded lizard[53-55]. CRISPs contain conserved C-terminal helical bundle subdomains termed ion channel regulatory (ICR) domains. ICR domains show homology and similar disulfide-bonding patterns to peptide toxins, BgK and ShK, which are potent inhibitors of K channels isolated from the sea anemones, *Bunodosoma granulifera*[56, 57] and *Stichodactyla helianthus*[58, 59], respectively. For example, triflin, ablomin, latisemin, ophanin and piscivorin block smooth muscle contruction caused by inhibition of L-type Ca^{2+} channels. Naturin from *Naja atra* acts as a blocker of BK_{Ca} channels[60]. On the other hand, unique CRISP family proteins, pseudechetoxin (PsTx) and pseudecin, which block olfactory and retinal cyclic nucleotide-gated ion (CNC)

channels, and crovirin, which shows anti-parasitic activity against infective trypanosoma forms have been identified[61]. Thus, venom CRISPs show specific activities against a variety of ion channels and possess various physiological activities. To date, cDNAs (transcriptome data) for triflin (AF384219.1, AB848115.1, AB985232.1) and CRISPs with EGF-like domains (AB851959.1) have been identified in *P. flavoviridis* snake venom[50,32].

The present study identified four CRISP-related genes, *CRISP01* to *CRISP04*, in the *P. flavoviridis* genome (Supplementary Fig. S14a). The genes encoding triflin (*CRISP01*; Gene IDs: habu1_s22025_g22470a) and a novel triflin-like peptide (*CRISP02*; Gene IDs: habu1_s22025_g22470b) were located in the same scaffold, habu1_scaffold_22025 (Acc no. BFFQ01031149) (Supplementary Fig. S14a). On the other hand, genes encoding two types of CRISP with an EGF-like domain, *CRISP03* (Gene ID: habu1_s1243_g04322) and *CRISP04* (Gene ID: habu1_s264_g01336), were located on habu1_scaffold_1243 (Acc no. BFFQ01000866) and habu1_scaffold_264 (Acc no. BFFQ01000220), respectively (Supplementary Fig. S14a). Interestingly, although both triflin and triflin-like genes contain eight exons (ex1 to ex8 for triflin, and ex1a, ex1b, ex2 to ex7 for triflin-like) and their N-terminal regions including signal sequences and pathogenesis-related CRISP domains corresponding to exons 2 to 5 were conserved, the number and structures of exons encoding C-terminal regions, including ICR domains were quite different (Supplementary Figs. S14c and S14d). These results suggest that *CRISP/triflin* and *triflin-like* genes arose through the gene duplication and exon shuffling. On the other hand, genes encoding CRISPs with EGF-like domains

showed the same exon-intron architecture and homologies without a C-terminal extension of *CRISP04*. Molecular phylogeny supports these notions (Supplementary Fig. S14b)

(h) SPRY/Vespryns (Vespryin)

The vespryn family is a relatively recently discovered as a snake venom protein family containing consensus PRY-SPRY domains. The SPRY domain is an interaction module consisting of ~120 amino acids, which is implicated in the biological pathways including innate and adaptive immunity. The SPRY domain was first identified in SPlA (spore lysis A) kinase from *Dictyostelium discoideum*, and in mammalian ryanodine receptors[62, 63]. Ohanin, which was isolated from the king cobra, *Ophiophagus hannah* venom, shows dose-dependent hypolocomotive and hyperalgesic effects in mice[64, 65]. The present study identified a gene encoding vespryn (*Ves01*) and another 11 genes encoding SPRY domain-containing proteins (*Ves02* to *Ves12*) (Supplementary Fig. S15a). *Vespryn* gene includes 5 exons and 4 introns, while butyrophilin2A1 (btn2A1)-encoding gene (*Ves02*) includes 10 exons. Butyrophilins (BTN) are members of a protein family that belong to immunoglobulin superfamily that have immunomodulatory functions. Both vespryn and butyrophilin genes are located on the same scaffold, habu1_scaffold_402940 (Acc no. BFFQ01082524) (Supplementary Fig. S15a), and molecular phylogeny shows affinity or similarity of these two gene products (Supplementary Fig. S15b), indicating that vespryn is more likely to be derived from ancestor gene of butyrophilin.

(i) 5'-Nucleotidases (5Nase)

5'-nucleotidase (5Nase, EC: 3.1.3.5) dephosphorylates 5'-mononucleotides to release purine and pyrimidine nucleosides. Viperidae and crotalidae venoms display more 5'-nucleotidase activity than elapidae venoms[66]. 5Nase inhibits platelet aggregation due to the liberation of adenosine. Venom 5Nase act synergistically *in vivo* with other toxins such as ADPases, hemorrhagic proteases, fibrinogenases and disintegrins to exert a more pronounced anti-coagulant effect[67].

The present study identified one sv5Nase gene, *sv5Nase1* (habu1_s6028_g16570) (Supplementary Fig. S16a). In addition, the *P. flavoviridis* genome contained at least 10 nv5Nase (habu1_s1426_g05181 for *5Nase02* to habu1_s2849_g10145 for *5Nase11*) (Supplementary Fig. S16a). Molecular phylogeny shows that *sv5Nase01* form a clade with other SV genes while the other nv5Nase genes cluster with other reptile NV 5Nase genes (Supplementary Fig. S16b), indicating the affinity or similarity of the SV and NV gene products in the 5Nase01 clade. An ancestral gene appears to have diverged first into NV forms, and then one of them has been functionalized as a toxin (Supplementary Fig. S16b).

(j) Dipeptidyl Peptidase IV (DPP IV)

Dipeptidyl peptidases (DPP) (EC 3.4.14.-) cleave dipeptides from the N-termini of polypeptides, and are classified into nine distinct types (DPP I-IV, VI-X). Mammalian DPP-IV is a highly glycosylated serine protease and a type II membrane protein that

selectively cleaves N-terminal dipeptides, Xxx-Pro (or Ala) from polypeptides. DPP-IV has been detected on the surface of immune cells as being CD26, a cell-surface differentiation marker in the T cell linage[68], and also detected in serum[69] and seminal plasma[70] as a soluble form. More recently, it has been reported that DPP-IV plays as a processing enzyme for liberation of biologically active peptides in the skin of *Xenopus laevis*[71] and snake venoms[72]. It was proposed that svDPP-IV contribute to hypotension by destroying vasoconstrictive peptides such as Peptide YY, neuropeptide Y and substance P, and shows synergistic effect with BPPs-ACE inhibitory peptide[73].

The present study identified 8 genes encoding dipeptidyl peptidases, *DPP01* to *DPP08* (Supplementary Fig. S17a), one of which was venom svDPP-IV (*DPP05*, habu1_s1020_g03622). Molecular phylogeny supports the evolutionary history of svDPP-IV and other NV DPPs that diversified into 8 genes (Supplementary Fig. S17b).

(k) Hyaluronidases (Hyal)

Hyaluronidases (Hyals) (EC: 3.2.1.35) are enzymes that cleave predominantly the glycosaminoglycans, hyaluronan (HA) and less efficiently, chondroitin (Ch) and chondroitin sulphate (ChS) in the extracellular matrix (ECM) of animals[74]. HA participate in physiological processes such as embryogenesis, cell migration, wound healing, tissue turn over, and malignancies[75].

Hyaluronidase is also common component of snake venoms known as a venom spreading factor to degrade HA in ECM[76]. To date, the full-length cDNA sequences encoding snake venom Hyal (svHyal) have been reported from *Echis ocellatus*

(EOC00242; DQ840249), *Echis pyramidum leakeyi* (*Epl*Hy-1: DQ840253), *Bitis arientan*s (*Ba*Hy-1: DQ840256, *Ba*Hy-2: DQ840257), *Cerastes cerastes cerastes* (*Ccc*Hy-1: DQ840250, *Ccc*Hy-3: DQ840251, *Ccc*Hy-4: DQ840257)[77] and, *Bothrops pauloensis*[78]. They showed different primary structures compared with other Hyals.

In this study, we identified six genes encoding *P. flavoviridis* Hyals, *Hyal01* to *Hyal06* (Supplementary Fig. S2a). *Hyal01* (habu1_s7188_g17820) encodes a svHyal, which was composed of six exons. On the other hand, other Hyal genes, *Hyal02* (*HYAL1*), *Hyal03* (*HYAL2*), *Hyal04* (*HYAL2/4*), *Hyal05* (*HYAL3*) and *Hyal06* (*HYAL4*), showed quite different gene structures with different exon numbers (four to seven), although core exons (exons 4 and 5 in *svHyal*) were conserved in all genes. Molecular phylogeny supports such a relationship between *P. flavoviridis* hyaluronidase family members (Supplementary Fig. S2a).

(l) Nerve growth factor (NGF)

Nerve growth factor (NGF) is one of polypeptide hormones belonging to the neurotrophin family that is necessary for neuronal differentiation, survival, and maintenance. The neurotrophin family also includes brain-derived neurotrophic factor (BDNF)[79], neurotrophin-3 (NT-3)[80], and neurotrophin-4/5 (NT-4/5)[81], which interact with cell surface receptors, p75 and Trk subfamily receptors, and stimulate tyrosine phosphorylation of Trk receptors[82]. NGFs are present in the venoms of elapids and viperids[83, 84]. Cobra NGFs show lower biological activities than mouse NGF[85]. More

recently, Sunagar et al reported that a duplication of NGF genes occurred in *Elaphidae* snake, and discussed their putative roles in venom and their unique molecular evolution under the positive-selection[86]. Furthermore, Hargreaves *et al.* reported the transcriptomic analysis for body tissues and salivary and venom glands from five species of venomous and nonvenomous reptiles, and discussed their molecular evolution based on the expression profiles[87]. It suggested that snake venom does not evolve through the hypothesized process of duplication and recruitment of genes encoding body proteins, and that many proposed venom toxin genes have been restricted to the venom gland following duplication, not recruited. In the case for NGF, the nontoxic form of NGF is expressed in a diversity of tissues, including the salivary glands of nonvenomous reptiles. The putatively toxic NGF has therefore also been restricted to the venom gland following duplication[87].

In the *P. flavoviridis* genome, we identified a single gene encoding svNGF located on habu1_scaffold_3536 (*Neu01*, Gene ID: habu1_s3536_g11975) (Supplementary Fig. S2b), which consists of four exons (Ex1 to 4) as in chicken and mammalian NGFs. Interestingly, the expression profiles of *P. flavoviridis* NGF is specific to venom gland although several transcripts with different 5'exons were present (Supplementary Table S7). This alternative usage of 5' exons containing the 5'-untranslated region has been reported in the chicken NGF gene[88].

Furthermore, neurotrophin family genes encoding, *Neu02, 03 and 04*, BDNF (Gene ID: habu1_s3803_g12736), NT-3 (habu1_s105_g00548), and NT-4 (habu1_s6123_g16797), respectively, were identified in the genome (Supplementary Fig. S2b). Molecular

phylogeny indicates that *svNGF* and other three *BDNF*, *NT-3* and *NT-4* diverged first in their history (Supplementary Fig. S2b), suggesting that the neurotrophin family including NGF is a typical example of SV genes generated by 2R-WGD.

(m) Vascular endothelial growth factor-like proteins (VEGF)

Vascular endothelial growth factors (VEGFs) are key regulators of vascular development during embryogenesis (vasculogenesis), blood-vessel formation (angiogenesis), skeletal growth, and reproductive functions[89]. In mammals, five VEGFs (VEGF-A, -B, -C, -D, and –E (viral VEGF)) have been identified so far. These ligands bind to three receptor tyrosine kinases, VEGF receptor-1 (VEGFR1, Flt-1, *fms*-like tyrosine kinase-1), -2 (VEGFR2, KDR, kinase insert domain-containing receptor) and -3 (VEGFR3, Flt-4), as well as to co-receptors such as heparan sulphate glycans. VEGFs have been found in snake venoms, and they showed strict receptor specificities compared with mammalian VEGF. For example, Vammin and VR-1 from the venoms of *Vipera a. ammodytes* and *Daboia r. russelli*, respectively, bind only KDR with high affinity but not to other VEGF receptors[90]. *Tf*-svVEGF from *P.flavoviridis* venom have been shown to bind Flt-1 in preference to KDR, unlike vammin and VR-1[91]. The gene structures of tissue- and venom-types VEGFs from *P. flavoviridis* have been reported[92]. We identified three VEGF genes in the *P. flavoviridis* genome (Supplementary Fig. S18a). One is *svVEGF01* (habu1_s565_g02679) encoding svVEGF-F and the others are *nvVEGF01* and *nvVEGF02*, encoding *VEGF-A* (habu1_s6836_g17529) and *VEGF-C* (habu1_s9381_g19343), respectively. Molecular phylogeny indicates that *svVEGF-F* is

within toxin-related protein group (Supplementary Fig. S18b).

(n) L-Amino acid oxidases (LAAO)

L-Amino acid oxidases (LAAO: EC 1.4.3.2) are flavoenzymes that catalyze the oxidative deamination of L-amino acids to produce α-keto acids with the release of NH_3 and H_2O_2. LAAOs are abundant in some snake venoms and are cytotoxic due to the liberated H_2O_2, which also induces or inhibits platelet aggregation[93]. It was reported that solble guanylate cyclase (sGC) is activated by H_2O_2 in the presence of superoxide dismutase (SOD), and that H_2O_2 activates nitric oxide synthase (NOS). Thus, LAAOs show the synergic effects on hypertension with other toxins such as BPPs via H_2O_2 production[73].

The *P. flavoviridis* genome contains three genes for L-amino acid oxidase, *LAAO_01*, *LAAO_02*, and *LAAO_03* (Gene ID: habu1_s402940_g24950, *g24949,* and *g24947,* respectively)*,* all of them being located in the same scaffold (Supplementary Fig. S2c). The results indicate that these LAAO genes diversified by gene duplication. *LAAO_01* encodes the venom type L-amino acid oxidase composed of seven exons. Molecular phylogeny indicates that *LAAO_01* encodes the venom enzyme, but *LAAO_02*, and *LAAO_03* encode non-venom type (Supplementary Fig. S2c).

(o) Phosphodiesterases (PDE)

Phosphodiesterases (PDEs: E.C. 3.1.4.1) catalyze the hydrolysis of phosphodiester bonds of cyclic adenosine monophosphate (cAMP) and cyclic guanosine

monophosphate (cGMP). PDEs have been isolated and characterized from numerous snake venoms. They are generally high molecular mass proteins (> 90 kDa) with single polypeptide chains. *P. flavoviridis* venom PDEs are reported to be metalloglycoproteins[94]. More recently, Trummal et al. reported the structure and characterization of PDE from *Vipera lebetina* venom[95]. It inhibits ADP- and collagen-induced platelet aggregation in a dose-dependent manner. Mamillapalli *et al* reported that the activity of svPDE is enhanced by lysophospholipids, which are liberated by PLA2 during venomous action[96]. Thus, svPDE and svPLA2 show the synegistic effect.

In this study, we identified two PDE genes, *PDE01* (Gene ID: habu1_s149_g00804) and *PDE02* (habu1_s149_g008059), which are tandemly located on habu1_scaffold_149 (Supplementary Fig. S19a). Molecular phylogeny shows that *Pr. flavoviridis PDE01* is the venom enzyme and PDE02 is the tissue form (Supplementary Fig. S19b). PDE01 and PDE-02 include 889 and 904 amino acids, respectively, and both conserve catalytic amino acid residues. Although both genes are encoded by 25 exons with the same exon-intron junctions, they showed different expression profiles. PDE01 is expressed only in the venom gland. The cAMP-specific 0’, 5’-cyclic phosphodiesterase gene was also identified on the same scaffold (Gene ID: habu1_s149_g00834) (Supplementary Fig. S19a). However, it showed no sequence homology with *PDE01* and *PDE02*.

(p) Phospholipases B (PLB)

Phospholipases B (PLB: EC 3.1.1.5) are enzymes with combined PLA_1 and PLA_2 activities that cleaves acyl chains from both *sn*-1 and *sn*-2 positions of phospholipids. PLB also acts on lysolecithin, which is formed by PLA_2, then PLBs are known as lysophospholipases. PLBs are present in most snake venoms, but generally at very low levels[97].

In this study, we identified five PLB genes, *PLB01* (Gene ID: habu1_s1233_g04284), *PLB02* (habu1_s1964_g07200), *PLB03*, (habu1_s313_g01520), PLB04 (habu1_s3352_g11424), and PLB05 (habu1_s303_g01427) in the *P. flavoviridis* genome, (Supplementary Fig. S20a). *PLB01* showed high sequence identity (99-85%) with phospholipases B isolated from other snakes. On the other hand, *PLB02* is phospholipase B-like 2 and *PLB03* is a membrane-associated PLB1. PLB05 and PLB06 are 60 kDa lysophospholipase and lysophospholipase-like protein, respectively. *PLB01* and *PLB02* showed low but distinct sequence similarity (30.2%) each other, while PLB03, PLB04 and PLB05 showed no similarity. From transcriptome data, *PLB01* showed venom-specific expression, while *PLB02, PLB04 and PLB05* are ubiquitous housekeeping genes, and *PLB03* showed small intestine-specific expression. Molecular phylogeny supports the evolutionary history of SV and other NV genes (Supplementary Fig. S20b).

(q) Bradykinin-potentiating peptide (BPP) and C-type natriuretic peptide (CNP)

Bradykinin-potentiating peptides (BPPs) are known as painful stimulation factors following snakebites through the inhibition of enzymatic digestion of bradykinin. BPPs

are also well known as inhibitors of angiotensin-converting enzyme (ACE), and were the basis for development of antihypertensive drugs such as captopril and enalapril[98]. BPPs activate argininosuccinate synthetase, resulting in increased nitric oxide production, which reduces arterial blood pressure[99]. Typically, BPPs contain 5 to 13 amino acid residues with a pyroglutamyl residue (pGlu) at the N-terminus and a proline residue at the C-terminus a high proline content and/or the tripeptide sequence Ile–Pro–Pro[100].

Natriuretic peptide (NP) plays a fundamental role in cardiovascular homeostasis by modulating fluid and electrolyte balance and vascular tone, and exhibit hypotensive and vasodepressor activity by activating natriuretic receptors. Previously, we have cloned cDNAs (NCBI AB749764.1) encoding both BPPs and C-type naturetic peptide (CNP) from *P. flavoviridis* and other snakes[101, 102], suggesting that CNP coded on the same gene as BPP. Recently, Aird *et al.* reported the transcriptomes and proteomes of *P. flavoviridis* and *Ovophis okinavensis* venom, showed only two BPP-related peptides, QSKPGRSPPISP, which corresponds to BPP1_3, and QGRPRSEVPP[101].

In the *P. flavoviridis* genome, we identified two genes. *CNP01* encodes a BPP-CNP precursor protein, which was located at two scaffolds, habu1_scaffold_258676: 1...337 and habu1_scaffold_4348: 15402 ... 16141 (Gene model ID: habu1_s258676_g24318 and habu1_s4348_g14020, respectively) (Supplementary Fig. S21a). *CNP02* encodes B-type natriuretic peptide (BNP) (habu1_s20540_g224311) (Supplementary Fig. S21a). The former is venom peptides, while the latter is of another tissue origin. Molecular phylogeny supports the evolutionary history of SV and other NV genes (Supplementary

Fig. S21b).

(r) Glutaminyl peptide cyclotransferases (GPCase)

Glutaminyl peptide cyclases (GPCase, QC; glutaminyl cyclotransferase, EC 2.3.2.5) catalyze N-terminal pyroglutamate (pGlu) formation on proteins and peptides. This N-terminal modification confers resistance to aminopeptidase degradation. Among various snake venom components, glutaminyl cyclase (svQC) is one of the least understood protein family. Wang et al. reported the presence of vQC activity in a wide spectrum of venom species and their structures and characterization including cDNA sequences from seven species[103]. svQC present in each venom with the low content and only a single form, suggesting its house-keeping role for posttranslational modification of the venom proteins. For example, svQC cyclizes the N-terminal Gln residue of snake venom bioactive peptides and proteins including BPPs[100,104] and crotoxin subunits[105], resulting the stability and prolongation of peptides in prey plasma by protecting against the degradation.

We identified two genes, *svGPCase01* (habu1_s510_g02384) and *nvGPCase02* (habu1_s3067_g10859), encoding glutaminyl peptide cyclases in *P. flavoviridis* genome (Supplementary Fig. S22a). Both *svGPCase01* and *nvGPCase02* were composed of seven exons with the conserved exon-intron junctions. Recently, Aird et al. reported four QC transcripts for two pairs of toxins (AB848133, AB848134, AB851933, AB851934)[106], the correspondence of those to *svGPCase01* and *nvGPCase02* remains to be elucidated. Molecular phylogeny shows that *svGPCase01* forms a clade with other

SV members while *nvGPCase02* is grouped with other NV members (Supplementary Fig. S22b). This indicates that the product of *svGPCase01* is sure to act as toxin or toxin stabilizing factor in *P. flavoviridis* venom although QC activity has not yet been demonstrated in the venom.

References

1. Fox, J. W, & Serrano, S. M. T. Structural considerations of the snake venom metalloproteinases, key members of the M12 reprolysin family of metalloproteinases. Toxicon 2005; 45, 969–985.
2. Worlfsberg, T. G. et al. ADAM, a widely distributed and developmentally regulated gene family encoding membrane proteins with a disintegrin and metalloprotease domain. Devlop Biol. 1995; 169, 378-383.
3. Edwards, D. R., Handsley, M. M., Pennington, C. J. The ADAM metalloproteinases. Mol. Asp. Med. 2009; 29, 258–289.
4. Reiss K, Saftig P. The “A Disintegrin and Metalloprotease” (ADAM) family of sheddases: Physiological and cellular functions. Semin Cell Dev Biol. 2009; 20: 126–37.
5. Takeda S. ADAM and ADAMTS family proteins and snake venom metalloproteinases: A structural overview. Toxins. 2016; 8: 155.
6. Seeger WH, Ouyang C. Snake venoms and blood coagulation, In: Lee CY editor. Snake venoms. New York: Springer; 1979. p. 684-750.

7. Shieh TC, Kawabata S, Kihara H, Ohno M, Iwanaga S. Purification and characterization of a coagulant enzyme from *Trimeresurus flavoviridis* venom. J Biochem. 1985; 98: 713–61.

8. Deshimaru M, Ogawa T, Nakashima K, Nobuhisa I, Chijiwa T, Shimohigashi Y, et al. Accelerated evolution of crotalinae snake venom gland serine proteases. FEBS Lett. 1996; 397: 83-8.

9. Sunagawa M, Nakamura M, Kosugi T. Cloning of habutobin cDNA and antithrombotic activity of recombinant protein. Biochem Biophys Res Commun. 2007; 362: 899-904.

10. Yamamoto C, Tsuru D, Oda-Ueda N, Ohno M, Hattori S, Kim ST. Flavoxobin, a serine protease from *Trimeresurus flavoviridis* (habu snake) venom, independently cleaves Arg726-Ser727 of human C3 and acts as a novel, heterologous C3 convertase. Immunology. 2002; 107: 111-7.

11. Komori Y, Tatematsu R, Tanida S, Nikai T. Thrombin-like enzyme, flavovilase, with kinin-releasing activity from *Trimeresurus flavoviridis* (habu) venom. J Nat Toxins. 2001; 10: 239-48.

12. Tatematsu R, Komori Y, Nikai T. A new thrombin-like enzyme, flavoviridiobin from the venom of *Trimeresurus flavoviridis* (habu). J Nat Toxins. 2000; 9: 327-39.

13. Serrano SM, Hagiwara Y, Murayama N, Higuchi S, Mentele R, Sampaio CA, et al. Purification and characterization of a kinin-releasing and fibrinogen-clotting serine proteinase (KN-BJ) from the venom of *Bothrops jararaca*, and molecular

cloning and sequence analysis of its cDNA. Eur J Biochem. 1998; 251: 845-53.

14. van Leeuwen BH, Evans BA, Tregear G, Richards RI. Mouse glandular kallikrein gene identification, structure, and expression of the renal kallikrein gene. J Biol Chem. 1986; 261: 5529-35.

15. Drickamer K. Evolution of Ca(2+)-dependent animal lectins. Prog Nucl Acid Res Mol Biol. 1993; 45: 207–32.

16. Drickamer K, Fadden AJ. Genomic analysis of C-type lectins. Biochem Soc Symp. 2002; 69: 59–72.

17. Zelensky AN, Gready JE. The C-type lectin-like domain superfamily. FEBS J. 2005; 272: 6179–217.

18. Flexner S, Noguchi H. The constitution of snake venom and snake sera. J Pathol. 1903; 8: 379-410.

19. Gartner TK, Stocker K, Williams DC. Thrombolectin: a lectin isolated from *Bothrops atrox* venom. FEBS Lett. 1980; 117: 13–6.

20. Sartim MA, Sampaio SV. Snake venom galactoside-binding lectins: a structural and functional overview. J Venom Anim Toxins Incl Trop Dis. 2015; 21: 35.

21. Ogawa T, Chijiwa T, Oda-Ueda N, Ohno M. Molecular diversity and accelerated evolution of C-type lectin-like proteins from snake venom. Toxicon. 2005; 45: 1-14.

22. Morita T. Structures and functions of snake venom CLPs (C-type lectin-like proteins) with anticoagulant-, procoagulant-, and platelet-modulating activities. Toxicon 2005; 45: 1099-114.

23. Dijkstra BW, Drenth J, Kalk KH. Active site and catalytic mechanism of phospholipase A2. Nature 1981; 289: 604-6.

24. Dufton MJ, Hider RC. Classification of phospholipases A_2 according to sequence. Evolutionary and pharmacological implications. Eur J Biochem. 1983; 137: 545-51.

25. Maraganore JM, Merutka G, Cho W, Welches W, Kézdy FJ, Heinrikson RL. A new class of phospholipases A2 with lysine in place of aspartate 49. Functional consequences for calcium and substrate binding. J Biol Chem. 1984; 259: 13839-43.

26. Renetseder R, Brunie S, Dijkstra BW, Drenth J, Sigler PB. A comparison of the crystal structures of phospholipase A_2 from bovine pancreas and *Crotalus atrox* venom. J Biol Chem. 1985; 260: 11627-34.

27. Holland DR, Clancy LL, Muchmore SW, Ryde TJ, Einspahr HM, Finzel BC, et al. The crystal structure of a lysine 49 phospholipase A2 from the venom of the cottonmouth snake at 2.0-Å resolution. J Biol Chem. 1990; 265: 17649-56.

28. Suzuki A, Matsueda E, Yamane T, Ashida T, Kihara H, Ohno M. Crystal structure analysis of phospholipase A2 from *Trimeresurus flavoviridis* (Habu snake) venom at 1.5 Å resolution. J Biochem. 1995; 117: 730-40.

29. Ikeda N, Chijiwa T, Matsubara K, Oda-Ueda N, Hattori S. Matsuda Y, Ohno M. Unique structural characteristics and evolution of a cluster of venom phospholipase A_2 isozyme genes of *Protobothrops flavoviridis* snake. Gene. 2010; 461: 15-25.

30. Nakashima K, Ogawa T, Oda N, Hattori M, Sakaki Y, Kihara H, Ohno M. Accelerated evolution of *Trimeresurus flavoviridis* venom gland phospholipase A_2 isozymes. Proc Natl Acad Sci U S A. 1993; 90: 5964-8.

31. Nakashima, K. et al. Accelerated evolution in the protein-coding regions is universal in crotalinae snake venom gland phospholipase A_2 isozyme genes. *Proc. Natl. Acad. Sci. USA.* **92**, 5605-5609 (1995)

32. Ohno, M. et al. Molecular evolution of snake toxins: Is the functional diversity of snake toxins associated with a mechanism of accelerated evolution? *Prog. Nucleic Acid Res. Mol. Biol.* **59**, 307-364 (1998).

33. Ogawa, T. et al. Unusually high conservation of untranslated sequences in cDNAs for *Trimeresurus flavoviridis* phospholipase-A2 isozymes. *Proc. Natl. Acad. Sci. USA.* **89**, 8557-8561 (1992).

34. Ogawa, T., Kitajima, M., Nakashima, K., Sakaki, Y. & Ohno, M. Molecular evolution of group II phospholipases A2. J. Mol. Evol. **41**, 867-877 (1995).

35. Nair DG, Fry BG, Alewood P, Kumar PP, Kini RM. Antimicrobial activity of omwaprin, a new member of the waprin family of snake venom proteins. Biochem J. 2007; 402: 93-104.

36. Fry, B. G. et al. Evolution of an arsenal: structural and functional diversification of the venom system in the advanced snakes (Caenophidia). *Mol. Cell Proteomics.* **7**, 215-46 (2008).

37. St Pierre Let al. Common evolution of waprin and kunitz-like toxin families in Australian venomous snakes. Cell Mol Life Sci. 2008; 65: 4039-54.

38. Pahari S, Mackessy SP, Kini MR. The venom gland transcriptome of the Desert Massasauga Rattlesnake (Sistrurus catenatus edwardsii): towards an understanding of venom composition among advanced snakes (Superfamily Colubroidea). BMC Mol Biol. 2007; 8: 115.

39. Doley R, Pahari S, Reza M. A., Mackessy SP, Kini RM. The gene structure and evolution of ku-wap-fusin (Kunitz Waprin Fusion Protein), a novel evolutionary intermediate of the Kunitz serine protease inhibitors and Waprins from *Sistrurus catenatus* (Massasauga Rattlesnake) venom glands. The Open Evolution J. 2010; 4: 31-41.

40. Mourao CBF, Schwartz EF. Protease inhibitors from marine venomous animals and their counterparts in terrestrial venomous animals. Mar Drugs. 2013; 11: 2069-112.

41. Bohlen CJ, Chesler AT, Sharif-Naeini R, Medzihradszky KF, Zhou S, King D, et al. A heteromeric Texas coral snake toxin targets acid-sensing ion channels to produce pain. Nature. 2011; 479: 410–4.

42. Utkin YN. Three-finger toxins, a deadly weapon of elapid venom--milestones of discovery. Toxicon. 2013; 62: 50-5.

43. Sunagar K, Jackson TN, Undheim EA, Ali SA, Antunes A, Fry BG. Three-fingered RAVERs: Rapid accumulation of variations in exposed residues of snake venom toxins. Toxins. 2013; 5: 2172-208.

44. Ménez A. Functional architectures of animal toxins: a clue to drug design? Toxicon. 1998; 36: 1557–72.

45. Tsetlin V. Snake venom α-neurotoxins and other 'three-finger' proteins. Eur J Biochem. 1999; 264: 281–6.

46. Junqueira-de-Azevedo IL, Ching AT, Carvalho E, Faria F, Nishiyama MY Jr, Ho PL, Diniz MR. *Lachesis muta* (Viperidae) cDNAs reveal diverging pit viper molecules and scaffolds typical of cobra (Elapidae) venoms: implications for snake toxin repertoire evolution. Genetics. 2006; 173: 877-89.

47. Ogawa Y, Murayama N, Fujita Y, Yanoshita R. Characterization and cDNA cloning of aminopeptidase A from the venom of *Gloydius blomhoffi brevicaudus*. Toxicon. 2007; 49: 1172–81.

48. Burnett LA, Washburn CA, Sugiyama H, Xiang X, Olson JH, Al-Anzi B, Bieber AL, Chandler DE. Allurin, an amphibian sperm chemoattractant having implications for mammalian sperm physiology. Int Rev Cell Mol Biol. 2012; 295: 1–61.

49. Yamazaki Y, Brown RL, Morita T. Purification and cloning of toxins from elapid venoms that target cyclic nucleotide-gated ion channels. Biochemistry. 2002a; 41: 11331–7.

50. Yamazaki Y, Koike H, Sugiyama Y, Motoyoshi K, Wada T, Hishinuma S, et al. Cloning and characterization of novel snake venom proteins that block smooth muscle contraction. Eur J Biochem. 2002b; 269: 2708–15.

51. Yamazaki Y, Hyodo F, Morita T. Wide distribution of cysteine-rich secretory proteins in snake venoms: isolation and cloning of novel snake venom cysteine-rich secretory proteins. Arch Biochem Biophys. 2003; 412: 133–41.

52. Yamazaki Y, Morita T. Structure and function of snake venom cysteine-rich secretory proteins. Toxicon. 2004; 44: 227-31.

53. Mochca-Morales J, Martin BM, Possani LD. Isolation and characterization of helothermine, a novel toxin from *Heloderma horridum horridum* (Mexican beaded lizard) venom. Toxicon. 1990; 28: 299–309.

54. Morrissette J, Kratzschmar J, Haendler B, El-Hayek R, Mochca-Morales J, Martin BM, et al. Primary structure and properties of helothermine, a peptide toxin that blocks ryanodine receptors. Biophys J. 1995; 68: 2280–8.

55. Nobile M, Noceti F, Prestipino G, Possani LD. Helothermine, a lizard venom toxin, inhibits calcium current in cerebellar granules. Exp Brain Res. 1996; 110: 15–20.

56. Cotton J, Crest M, Bouet F, Alessandri N, Gola M, Forest E, et al. A potassium-channel toxin from the sea anemone *Bunodosoma granulifer*a, an inhibitor for Kv1 channels. Revision of the amino acid sequence, disulfide-bridge assignment, chemical synthesis, and biological activity. Eur J Biochem. 1997; 244: 192–202.

57. Dauplais M, Lecoq A, Song J, Cotton J, Jamin N, Gilquin B, et al. On the Convergent Evolution of Animal Toxins Conservation of a diad of functional residues in potassium channel-blocking toxins with unrelated structures. J Biol Chem. 1997; 272: 4302–9.

58. Castañeda O, Sotolongo V, Amor AM, Stöcklin R, Anderson AJ, Harvey AL, et al. Characterization of a potassium channel toxin from the Caribbean Sea anemone *Stichodactyla helianthus*. Toxicon 1995; 33: 603–13.

59. Tudor JE, Pallaghy PK, Pennington MW, Norton RS. Solution structure of ShK toxin, a novel potassium channel inhibitor from a sea anemone. Nat Struct Biol. 1996; 3: 317–20.

60. Wang F, Li H, Liu M, Song H, Han H, Wang Q, et al. Structural and functional analysis of natrin, a venom protein that targets various ion channels. Biochem Biophys Res Commun. 2006; 315: 443–8.

61. Adade CM, Carvalho ALO, Tomaz MA, Costa TFR, Godinho JL, Melo PA, et al. Crovirin. A snake venom cysteine-rich secretory protein (CRISP) with promising activity against tryptozoma. PLoS Negl Trop Dis. 2014; 8: e3252.

62. Ponting C, Schultz J, Bork P. SPRY domains in ryanodine receptors (Ca^{2+}-release channels). Trends Biochem Sci. 1997; 22: 193–4.

63. Woo JS, Imm JH, Min CK, Kim KJ, Cha SS, Oh BH. Structural and functional insights into the B30.2/SPRY domain. EMBO J. 2006; 25: 1353-63.

64. Pung YF, Wong PTH, Kumar PP, Hodgson WC, Kini RM. Ohanin, a novel protein from king cobra venom, induces hypolocomotion and hyperalgesia in mice. J Biol Chem. 2005; 280: 13137–47.

65. Pung YF, Kumar SV, Rajagopalan N, Fry BG, Kumar PP, Kini RM. Ohanin, a novel protein from king cobra venom: its cDNA and genomic organization. Gene 2006; 371: 246-56.

66. Aird SD. Taxonomic distribution and quantitative analysis of free purine and pyrimidine nucleosides in snake venoms. Comp Biochem Physiol B Biochem Mol Biol. 2005; 140: 109–26.

67. Dhananjaya BL, D'Souza CJM. The pharmacological role of nucleotidases in snake venoms. Cell Biochem Funct 2010; 28: 171–7.

68. Gorrell MD, Gysbers V, McCaughan GW. CD26: a multifunctional integral membrane and secreted protein of activated lymphocytes. Scand J Immunol. 2001; 54: 249–64.

69. Maes M, Scharpé S, Demeester I, Goossens P, Wauters A, Neels H, et al. Components of biological variation in prolyl endopeptidase and dipeptidyl-peptidase-iv activity in plasma of healthy subjects. Clin Chem. 1994; 40: 1686-91.

70. Ohkubo I, Huang K, Ochiai Y, Takagaki M, Kani K. Dipeptidyl peptidase IV from porcine seminal plasma: purification, characterization, and N-terminal amino acid sequence. J Biochem. 1994; 116: 1182-6.

71. Mollay C, Vilas U, Hutticher A, Kreil G. Isolation of a dipeptidyl aminopeptidase, a putative processing enzyme, from skin secretion of *Xenopus laevis*. Eur J Biochem. 1986; 160: 31–5.

72. Ogawa Y, Mamura Y, Murayama N, Yanoshita R. Characterization and cDNA cloning of dipeptidyl peptidase IV from the venom of *Gloydius blomhoffi brevicaudus*. Comp Biochem Physiol B Biochem Mol Biol. 2006; 145: 35-42.

73. Aird SD. Ophidian envenomation strategies and the role of purines. Toxicon 2002; 40: 335-393.

74. Stern R, Jedrzejas MJ. Hyaluronidases: their genomics, structures, and mechanisms of action. Chem Rev. 2006; 106: 818–39.

75. Toole BP. Hyaluronan: from extracellular glue to pericellular cue. Nat Rev Cancer. 2004; 4: 528-39.

76. Kemparaju K, Girish KS. Snake venom hyaluronidase: a therapeutic target. Cell Biochem Function. 2006; 24: 7–12.

77. Harrison RA, Ibison F, Wilbraham D, Wagstaff SC. Identification of cDNAs encoding viper venom hyaluronidases: cross-generic sequence conservation of full-length and unusually short variant transcripts. Gene 2007; 392: 22-33.

78. Castanheira LE, Rodrigues RS, Boldrini-França J, Fonseca FPP, Henrique-Silva F, Homsi-Brandeburgo MI, Rodrigues VM. Molecular cloning of a hyaluronidase from *Bothrops pauloensis* venom gland. J Venom Anim Toxins Incl Trop Dis. 2014; 20: 25.

79. Hohn A, Leibrock J, Bailey K, Barde Y-A. Identification and characterization of a novel member of the nerve growth factor/brain-derived neurotrophic factor family. Nature. 1990; 344: 339-41.

80. Maisonpierre PC, Belluscio L, Squinto S, Ip NY, Furth ME, Lindsay RM, Yancopoulos GD. Neurotrophin-3: a neurotrophic factor related to NGF and BDNF. Science. 1990; 247: 1446-51.

81. Ibanez CF, Ernfors P, Timmusk T, Ip NY, Arenas E, Yancopoulos GD, Persson

H. Neurotrophin-4 is a target-derived neurotrophic factor for neurons of the trigeminal ganglion. Development 1993; 117: 1345-53.

82. Wong V, Arriaga R, Ip NY, Lindsay RM. The neurotrophins BDNF, NT-3 and NT-4/5, but not NGF, up-regulate the cholinergic phenotype of developing motor neurons. Eur J Neurosci. 1993; 5: 466-74.

83. Kostiza T, Meier J. Nerve growth factors from snake venoms: Chemical properties, mode of action and biological significance. Toxicon 1996; 34: 787–806.

84. Trummal K, Tõnismägi K, Paalme V, Järvekülg L, Siigur J, Siigur E. Molecular diversity of snake venom nerve growth factors. Toxicon 2011; 58: 363–8.

85. Hayashi K, Inoue S, Ikeda K. Purification and characterization of nerve growth factors (NGFs) from the snake venoms. In: Singh BR and Tu AT editors. Natural Toxins 2: Structure, Mechanism of Action, and Detection. 1996; 391: p. 403-16.

86. Sunagar K, Fry BG, Jackson TNW, Casewell NR, Undheim EAB, Vidal N, Ali SA, King GF, Vasudevan K, Vasconcelos V, Antunes A. Molecular Evolution of Vertebrate Neurotrophins: Co-Option of the Highly Conserved Nerve Growth Factor Gene into the Advanced Snake Venom Arsenalf. Plos ONE 2013; 8: e81827

87. Hargreaves AD, Swain MT, Hegarty MJ, Logan DW, Mulley JF. Restriction and Recruitment—Gene Duplication and the Origin and Evolution of Snake Venom Toxins. Genome Biol. Evol. 2014; 6: 2088–2095. doi:10.1093/gbe/evu166

88. Bertaux O, Toselli-Mollereau E, Auffray C, Devignes M-D. Alternative usage of 5' exons in the chicken nerve growth factor gene: refined characterization of a weakly expressed gene. Gene 2004; 334: 83-97.

89. Ferrara N, Gerber HP, LeCouter J. The biology of VEGF and its receptors. Nat Med. 2003; 9: 669-76.

90. Yamazaki Y, Takani K, Atoda H, Morita T. Snake venom vascular endothelial growth factors (VEGFs) exhibit potent activity through their specific recognition of KDR (VEGF receptor 2). J Biol Chem. 2003; 278: 51985-8.

91. Takahashi H, Hattori S, Iwamatsu A, Takizawa H, Shibuya M. A novel snake venom vascular endothelial growth factor (VEGF) predominantly induces vascular permeability through preferential signaling via VEGF receptor-1. J Biol Chem. 2005; 279: 46304-14.

92. Yamazaki Y, Matsunaga Y, Tokunaga Y, Obayashi S, Saito M, Morita T. Snake venom vascular endothelial growth factors (VEGF-Fs) exclusively vary their structures and functions among species. *J Biol Chem.* 2009; 284: 9885–91.

93. Guo C, Liu S, Yao Y, Zhang Q, Sun MZ. Past decade study of snake venom L-amino acid oxidase. Toxicon 2012; 60: 302-11.

94. Kini RM, Gowda TV. Rapid method for separation and purification of four isoenzymes of phosphodiesterase from *Trimeresurus flavoviridis* (Habu snake) venom. J Chromatogr. 1984; 291: 299-305.

95. Trummal K, Aaspõllu A, Tõnismägi K, Samel M, Subbi J, Siigur J, Siigur E Phosphodiesterase from *Vipera lebetina* venom structure and characterization. Biochimie. 2014; 106, 48-55.

96. Mamillapalli R, Haimovitz R, Ohad M, Shinitzky M. Enhancement and inhibition of snake venom phosphodiesterase activity by lysophospholipids. FEBS Letters 1998; 436: 256-258.

97. Doery HM, Pearson JE. Phospholipase B in snake venoms and bee venom. Biochem J. 1964; 92: 599-602.

98. Smith CG, Vane JR. The discovery of captopril. FASEB J. 2003; 17: 788–9.

99. Camargo AC, Ianzer D, Guerreiro JR, Serrano SM. Bradykinin-potentiating peptides: beyond captopril. Toxicon 2012; 59: 516-23.

100. Cotton J, Hayashi MA, Cuniasse P, Vazeux G, Ianzer D, De Camargo AC, Dive V. Selective inhibition of the C-domain of angiotensin I converting enzyme by bradykinin potentiating peptides. Biochemistry 2002; 41: 6065–71.

101. Higuchi S, Murayama N, Saguchi K, Ohi H, Fujita Y, Camargo AC, et al. Bradykinin-potentiating peptides and C-type natriuretic peptides from snake venom. Immunopharmacol. 1999; 44: 129-35.

102. Murayama N, Hayashi MA, Ohi H, Ferreira LA, Fernandes BL, Yamane T, Camargo ACM. Cloning and sequence analysis of a *Bothrops jararaca* cDNA encoding a precursor of seven bradykinin-potentiating peptides and a C-type natriuretic peptide. Proc Natl Acad Sci U S A. 1997; 94: 1189–93.

103. Wang YM, Huang KF, Tsai IH. Snake venom glutaminyl cyclases: Purification,

cloning, kinetic study, recombinant expression, and comparison with the human enzyme. Toxicon 2014; 86: 40-50.

104. Calvete JJ, Fasoli E, Sanz L, Boschetti E, Righetti PG. Exploring the venom proteome of the western diamondback rattlesnake, *Crotalus atrox*, via snake venomics and combinatorial peptide ligand library approaches. J Proteome Res 2009; 8: 3055–3067.

105. Aird SD, Yates JR III, Martino PA, Shabanowitz J, Hunt DF, Kaiser II: The amino acid sequence of the acidic subunit B-chain of crotoxin. Biochim Biophys Acta 1990; 1040: 217–224.

106. Aird SD, Watanabe Y, Villar-Briones A, Roy MC, Terada K, Mikheyev AS. Quantitative high-throughput profiling of snake venom gland transcriptomes and proteomes (*Ovophis okinavensis* and *Protobothrops flavoviridis*). BMC Genomics. 2013; 14: 790.

Supplementary Table S1. Summary of Roche 454 reads and Miseq and Hiseq reads of the *Protobothrops flavoviridis* genome.

Supplementary Table S2. Summary statistics of the Habu genome assembly, HabAm1.

Supplementary Table S3. Comparison of genome sequence assemblies among four snakes.

Supplementary Table S4. RNA-seq analysis of tissues and organs of *Protobothrops flavoviridis.*

Supplementary Table S5. Keywords used to identify venom-related proteins.

Supplementary Table S6. Best-fit model for estimating the phylogenetic trees.

Supplementary Table S7. Expression profiles of neutrophin family genes in *Protobothrops flavoviridis*.

Supplementary Figures

Supplementary Figure S1. Genome size estimation based on flow cytometry and *k*-mer analysis. **a,** Flow cytometry analysis of *Gallus gallus* (blue), *Protobothrops flavoviridis* (green), and *Eublepharis macularius* (red). The vertical axis indicates the number of nuclei counted. The horizontal axis indicates fluorescence intensity using an FL2-H filter. Simple linear regression using the two known genome sizes gave a rough estimate of the *P. flavoviridis* genome size at 1.8 Gb. **b,** *k*-mer distribution of whole genome shotgun sequencing reads of *P. flavoviridis* ($k = 27$). The vertical axis indicates the number of *k*-mers. The horizontal axis indicates *k*-mer coverage. Total number of *k*-mers ($k = 27$) was 65,661,771,084. Peak coverage was 43x. The left peak at low frequency (< 10x depth) likely results from mismatches due to heterozygous SNPs. The genome size was estimated to be 1.41 Gb (total length of used reads / peak coverage).

Supplementary Figure S2. Summary and molecular phylogeny of SV and NV genes of the hyaluronidase (Hyal) family (a), the Nerve growth factor (NGF) family (b), and the L-amino acid oxidase (LAAO) family (c). Multiple sequence alignments were performed using Clustalw. Phylogenetic trees were constructed with the maximum likelihood method using IQ-TREE (http://www.iqtree.org) based on aligned amino acid sequences. Numbers on branches are bootstrap values with 1000x resampling.

Ac: *Anolis carolinensis*, Acc: *Agkistrodon contortrix contortrix*, Af: *Azemiops feae*, Am: *Alligator mississippiensis*, Ap: *Agkistrodon piscivorus*, Ar: *Aspidites ramsayi*, As:

Alligator sinensis, Ba: *Bitis arietans*, Bb: *Brachymeles bonitae*, Bcc: *Boa constrictor constrictor*, Bco: *Boa constrictor occidentalis*, Ben: *Boa constrictor nebulosa*, Bem: *Bellatorias major*, Bf: *Bungarus fasciatus*, Bia: *Bitis arietans*, Bj: *Bothrops jararacussu*, Bm: *Bungarus multicinctus*, Boa: *Bothrops asper*, Bp: *Bothrops pauloensis*, Bt: *Brachymeles tiboliorum*, Ca: *Crotalus adamanteus*, Car: *Calabaria reinhardtii*, Cc: *Cerastes cerastes*, Cdc: *Candoia superciliosa crombiei*, Cdt: *Crotalus durissus terrificus*, Ce: *Chilabothrus exsul*, Cg: *Chilabothrus granti*, Ch: *Crotalus horridus*, Cha: *Chilabothrus angulifer*, Chm: *Chelonia mydas*, Chss: *Chilabothrus strigilatus strigilatus*, Cm: *Chelonia mydas*, Cn: Cryptophis nigrescens, Coa: *Corallus annulatus*, Coc: *Corallus cookii*, Coh: *Corallus hortulanus*, Cor: *Corallus ruschenbergerii*, Cr: *Calloselasma rhodostoma*, Css: *Candoia superciliosa superciliosa*, Ct: *Cophosaurus texanus*, Cu: *Charina umbratica*, Cym: *Cyclophiops major*, Dc: *Drysdalia coronoides*, Dd: *Dipsosaurus dorsalis*, Dip: *Ditypophis* sp. AC-2011, Dr: *Daboia russelii*, Dv: *Demansia vestigiata*, Ea: *Epicrates alvarezi*, Ec: *Epicrates cenchria*, Em: *Eryx muelleri*, En: *Eunectes notaeus*, Eo: *Echis ocellatus*, Epl: *Echis pyramidum leakeyi*, Et: *Eryx tataricus*, Fp: *Feylinia polylepis*, Gb: *Gloydius blomhoffii*, Gg: *Gallus gallus*, Gh: *Gloydius halys*, Gi: *Gloydius intermedius*, Gj: *Gekko japonicus*, Gw: *Gambelia wislizenii*, Hs: *Hoplocephalus stephensii*, Lc: *Lepidophyma cuicateca*, Lm: *Lachesis muta*, Lv: *Lacerta viridis*, Mg: *Meleagris gallopavo*, Ml: *Macrovipera lebetina*, Nk: *Naja kaouthia*, Ns: *Naja sputatrix*, Nss: *Notechis scutatus scutatus*, Oh: *Ophiophagus hannah*, Om: *Oxyuranus microlepidotus*, Oo: *Ovophis okinavensis*, Oss: *Oxyuranus scutellatus scutellatus*, Pa: *Pseudechis australis*, Pb: *Python bivittatus*, Pc: *Phrynosoma*

coronatum, Pe: *Protobothrops elegans*, Pf: *Protobothrops flavoviridis*, Ph: *Phrynosoma hernandesi*, Phm: *Phalotris mertensi*, Phrm: *Phrynosoma mcallii*, Pm: *Protobothrops mucrosquamatus*, Pn: *Pseudonaja textilis*, Pp: *Pseudechis porphyriacus*, Psa: *Psammodromus algirus*, Pv: *Pogona vitticeps*, Pyc: *Python curtus*, Sb: *Sceloporus bicanthalis*, Sca: *Struthio camelus australis*, Sct: *Sistrurus tergeminus*, See: *Sistrurus catenatus edwardsi*, Sib: *Simalia boeleni*, Sjb: *Sinomicrurus japonicus boettgeri*, Sjj: *Sinomicrurus japonicus japonicus*, Sjt: *Sinomicrurus japonicus takarai*, Smi: *Sinomicrurus macclellandi iwasakii*, Tc: *Tropidechis carinatus*, Tcu: *Tropidophis curtus*, Tel: *Tropidophis greenwayi lanthanus,* Tg: *Trimeresurus gracilis*, Th: *Takydromus hsuehshanensis*, Ts: *Thamnophis sirtalis*, Tt: *Tropidophis taczanowskyi*, Uo: *Urosaurus ornatus*, Vu: *Vipera ursinii*, Wa: *Walterinnesia aegyptia*, Xv: *Xantusia vigilis*.

Supplementary Figure S3. Summary and molecular phylogeny of the Metalloproteinase (MP) family. SV genes (a), NV genes (b) and a molecular phylogeny of SV and NV genes from the metalloproteinase family (c-e). Phylogenetic trees were constructed with the maximum likelihood method using IQ-TREE (http://www.iqtree.org) based on aligned amino acid sequences. Numbers on branches are bootstrap values with 1000x resampling.

Aam: *Apteryx australis mantelli*, Ac: *Anolis carolinensis*, Acc: *Aquila chrysaetos canadensis*, Acl: *Agkistrodon contortrix laticinctus*, Af: *Aptenodytes forsteri*, Am: *Alligator mississippiensis*, Ap: *Agkistrodon piscivorus*, Apl: *Agkistrodon piscivorus*

leucostoma, As: *Alligator sinensis*, Ap: *Anas platyrhynchos*, Ba: *Bothrops atrox*, Bj: *Bothrops jararaca*, Brs: *Buceros rhinoceros silvestris*, Ca: *Crotalus adamanteus*, Cad: *Crotalus adamanteus*, Cdd: *Crotalus durissus durissus*, Cdc: *Crotalus durissus collilineatus*, Cg: *Cricetulus griseus*, Ch: *Crotalus horridus*, Cj: *Coturnix japonica*, Cm: *Chelonia mydas*, Cp: *Crocodylus porosus*, Cpb: *Chrysemys picta*, Cvv: *Crotalus viridis viridis*, Da: *Deinagkistrodon acutus*, Ec: *Echis coloratus*, Gh: *Gloydius halys*, Gi: *Gloydius intermedius*, Gj: *Gekko japonicus*, Gs: *Gloydius saxatilis*, Ml: *Macrovipera lebetina*, Oh: *Ophiophagus hannah*, Oo: *Ovophis okinavensis*, Pb: *Python bivittatus*, Pc: *Phalacrocorax carbo*, Pf: *Protobothrops flavoviridis*, Pj: *Protobothrops jerdonii*, Pm: *Protobothrops mucrosquamatus*, Ps: *Pelodiscus sinensis*, Sct: *Sistrurus tergeminus*, Sce: *Sistrurus catenatus edwardsi*, Smb: *Sistrurus miliarius barbouri*, Ta: *Trimeresurus albolabris*, Tg: *Trimeresurus gracilis*, Ths: *Thamnophis sirtalis*, Ts: *Thamnophis sirtalis*, Vaa: *Vipera ammodytes ammodytes*.

Supplementary Figure S4. Summary and molecular phylogeny of the Serine protease (SP) family.

SV genes (a), NV genes (b) and a molecular phylogeny of SV and NV genes of the serine protease family (c-d). Multiple sequence alignments were performed with ClustalW. Phylogenetic trees were constructed with the maximum likelihood method using IQ-TREE (http://www.iqtree.org) based on the aligned amino acid sequences. Numbers on branches are bootstrap values with 1000x resampling.

Ac: *Anolis carolinensis*, As: *Alligator sinensis*, Ap: *Anas platyrhynchos*, Bf: *Bungarus fasciatus*, Ca: *Crotalus adamanteus*, Ch: *Crotalus horridus*, Cm: *Chelonia mydas*, Ec: *Echis coloratus*, Gj: *Gekko japonicus*, Mf: *Micrurus fulviu*s, Ml: *Macrovipera lebetina*, Mr: *Macropisthodon rudis*, Mt: *Micrurus tener,* Ns: *Notechis scutatus*, Oh: *Ophiophagus hannah*, Oo: *Ovophis okinavensis*, Pa: *Pseudechis australis*, Pb: *Python bivittatus*, Pe: *Protobothrops elegans*, Pf: *Protobothrops flavoviridis*, Pm: *Protobothrops mucrosquamatus*, Pp: *Pseudechis porphyriacus*, Smb: *Sistrurus miliarius barbouri*, Ts: *Thamnophis sirtalis*.

Supplementary Figure S5. Summary and molecular phylogeny of the C-type lection-like protein (CTLP) family. SV genes (a), NV genes (b) and a molecular phylogeny of ten SV genes from the C-type lectin-like protein family (c). Multiple sequence alignments were performed with ClustalW. Phylogenetic trees were constructed with the maximum likelihood method using IQ-TREE (http://www.iqtree.org) based on the aligned amino acid sequences. Numbers on branches are bootstrap values with 1000 resampling.

Ac: *Anolis carolinensis*, Acc: *Agkistrodon contortrix contortrix*, Ap: *Agkistrodon piscivorus*, Ba: *Bitis arietans*, Bf: *Bungarus flaviceps*, Bj: *Bothrops jararaca*, Ca: *Crotalus adamanteus*, Ch: *Crotalus horridus*, Cn: *Cryptophis nigrescens*, Coh: *Crotalus oreganus helleri*, Cr: *Calloselasma rhodostoma*, Da: *Deinagkistrodon acutus*, Dv: *Demansia vestigiata*, Ecs: *Echis carinatus sochureki*, Gb: *Gloydius brevicaudus*, Gh: *Gloydius halys*, Lm: *Lachesis muta*, Mc: *Micrurus corallinus*, Ml: *Macrovipera*

lebetina, Mt: *Micrurus tener*, Ns: *Notechis scutatus*, Oh: *Ophiophagus hannah*, Pa: *Pseudechis australis*, Pb: *Python bivittatus*, Pf: *Protobothrops flavoviridis*, Pj: *Protobothrops jerdonii*, Pm: *Protobothrops mucrosquamatus*, Pn: *Parasuta nigriceps*, Pp: *Pseudechis porphyriacus*, Ps: *Pelodiscus sinensis*, Pt: *Pseudonaja textilis*, Sce: *Sistrurus catenatus edwardsi*, Ta: *Trimeresurus albolabris*, Ts: *Thamnophis sirtalis*

Supplementary Figure S6. Summary and molecular phylogeny of the Phospholipase A_2 (PLA2) family. SV genes (a), NV genes (b) and a molecular phylogeny of SV and NV genes of the phospholipase A_2 family (c). Multiple sequence alignments were performed using ClustalW. Phylogenetic trees were constructed by maximum likelihood method using IQ-TREE (http://www.iqtree.org) based on aligned amino acid sequences. Numbers on branches are bootstrap values with 1000x resampling.

Ac: *Anolis carolinensis*, Acc: *Agkistrodon contortrix contortrix*, Ap: *Agkistrodon piscivorus*, Ba: *Bitis arietans*, Bas: *Bothrops asper*, Bd: *Bothrops diporus*, Bj: *Bothrops jararaca*, Bl: *Bothrops leucurus*, Bm: *Bothrops moojeni*, Bs: *Bothriechis schlegelii*, Ca: *Crotalus adamanteus*, Cc: *Crotalus cerberus*, Cdt: *Crotalus durissus terrificus*, Cg: *Cerrophidion godmani*, Cmn: *Crotalus molossus nigrescens*, Coa:*Crotalus oreganus abyssus*, Coc: *Crotalus oreganus concolor*, Cpb: *Chrysemys picta bellii*, Cr: *Calloselasma rhodostoma*, Css: *Crotalus scutulatus scutulatus*, Gb: *Gloydius brevicaudus*, Gh: *Gloydius halys*, Gi: *Gloydius intermedius*, Gu: *Gloydius ussuriensis*, Lm: *Lachesis muta*, Oh: *Ophiophagus hannah*, Om: *Ovophis makazayazaya*, Oo:

Ovophis okinavensis, Pb: *Python bivittatus*, Pe: *Protobothrops elegans*, Pf: *Protobothrops flavoviridis*, Pm: *Protobothrops mucrosquamatus*, Scc: *Sistrurus catenatus catenatus*, Sct: *Sistrurus tergeminus*, Sm: *Sistrurus miliarius* , Smb: *Sistrurus miliarius barbouri*, Sms: *Sistrurus miliarius streckeri*, Ta: *Trimeresurus albolabris*, Tg: *Trimeresurus gracilis*, Ts: *Thamnophis sirtalis*.

Supplementary Figure S7. Three possible evolutionary scenarios. Thee possible evolutionary scenarios to explain the relationship between 2R-WGD and duplication of four ohnologs (A, B, C and D) into genes that encode venom proteins (red bars) and those that encode non-venom proteins (blue bars). 1R and 2R indicates first and second round WGD, respectively. (ABCD), (AB) and (CD) represent ancestral genes. (a) a molecular phylogeny showing the diversification occurred randomly in all ohnologs. (b) a molecular phylogeny showing the diversification occurred in one of the two ohnologs that were produced after the second round of WGD. (c) a molecular phylogeny showing only one ohnolog (in this case, ohnolog A) was duplicated to give rise to venom protein genes while in the three others, duplication did not involve the development of venom protein genes.

Supplementary Figure S8. Tandem duplications of SV genes. Seven examples of tandem duplications of MP (a), SP (b), and CTLP (c) genes. SV copies and NV copies are shown in red and grey arrows, respectively. Sizes of gene clusters are shown on the right side.

Supplementary Figure S9. Five examples of FISH mapping of cDNA clones to metaphase spreads of *Protobothrops flavoviridis* and *Elaphe quadrivirgata*. Arrowheads indicate hybridization signals. TEX14 (b, h), CBX2 (c, i) and AR (d, j) genes were mapped to chromosomes 1p, 2q, and a pair of microchromosomes, respectively, in *P. flavoviridis* (b, c, d) and *E. quadrivirgata* (h, i, j). Hoechst-stained G-banded pattern of the same metaphase chromosomes as in b and h is shown in a and g, respectively. While GAD2 genes mapped to Zq in *P. flavoviridis* (e) and Zp in *E. quadrivirgata* (k), EIF1 genes to Zp in *P. flavoviridis* (f) and Zq in *E. quadrivirgata* (l). Arm ratios of Z chromosomes were different between the two species due to a small pericentric inversion that occurred in Z chromosomes in the *Elaphe* lineage.

Supplementary Figure S10. Schematic diagram of the gene structures of the snake venom metalloproteinases (svMP), HV1, flavorase, VMP-III-like, jerdonitin-like, HR1a, H2-protease, HR2a, flavoviridin, HR1b, elegantin and NaMP-like. Red box shows the eight core exons (4 to 11) encoding metalloproteinase domains. HHH indicate catalytic sites for MP.

Supplementary Figure S11. Schematic structure of the snake venom serine protease (svSP) family genes. Flavoxobin (svSP01, TLf1), TLf2 (svSP02), TLf3 (svSP03), and svSP04 to svSP11 (a), and a gene structure comparison among representative serine proteases (b) are shown. These svSP genes essentially consist of 6

exons, the first of which contains only the 5'non-coding region, except for svSP06 having additional two exons Ex7 and Ex8, and svSP05 possessing a sub-exon, Ex3b. H, D, and S in the boxes indicate the catalytic triad. Arrowheads show the initiation codons.

Supplementary Figure S12. Summary and a phylogenetic tree of the three-finger toxin (3FTX) family. SV and NV genes (a) and a molecular phylogeny (b) of the 3FTX family. Multiple sequence alignments were performed using ClustalW. Phylogenetic trees were constructed with the maximum likelihood method using IQ-TREE (http://www.iqtree.org) based on aligned amino acid sequences. Numbers on branches are bootstrap values with 1000x resampling.

Af: *Azemiops feae*, Ahp: *Ahaetulla prasina*, Am: *Alligator mississippiensis*, Bc: *Bungarus candidus*, Bf: *Bungarus flaviceps*, Bi: *Boiga irregularis*, Bm: *Bungarus multicinctus*, Cm: *Chelonia mydas*, Cpb: *Chrysemys picta bellii*, Dt: *Dispholidus typus*, Lm: *Leioheterodon madagascariensis*, Mt: *Micrurus tener*, Nn: *Naja naja*, Oh: *Ophiophagus hannah*, Oo: *Ovophis okinavensis*, Pe: *Protobothrops elegans*, Pf: *Protobothrops flavoviridis*, Pm: *Protobothrops mucrosquamatus*, Psm: *Psammophis mossambicus*, Sce: *Sistrurus catenatus edwardsi*, Tb: *Trimorphodon biscutatus*, Td: *Telescopus dhara*, Tj: *Thrasops jacksonii*, Ths: *Thamnophis sirtalis*.

Supplementary Figure S13. Summary and a phylogenetic tree of the aminopeptidase family. SV and NV genes (a) and a molecular phylogeny (b) of the

aminopeptidase family. Multiple sequence alignments were performed using ClustalW. Phylogenetic trees were constructed with the maximum likelihood method using IQ-TREE (http://www.iqtree.org) based on aligned amino acid sequences. Numbers on branches are bootstrap values with 1000x resampling.

Ac: *Anolis carolinensis*, Br: *Bitis rhinoceros*, Gb: *Gloydius brevicaudus*, Oh: *Ophiophagus hannah*, Oo: *Ovophis okinavensis*, Pb: *Python bivittatus*, Pf: *Protobothrops flavoviridis*, Pm: *Protobothrops mucrosquamatus*, Ts: *Thamnophis sirtalis*.

Supplementary Figure S14. Summary and a phylogenetic tree and structure of the Cysteine-rich secretory protein (CRISP) family. SV and NV genes (a) and a molecular phylogeny (b) of the CRISP family. Comparison of amino-acid sequences between CRISP01 and 02 (c), and CRISP03 and 04 (d). Multiple sequence alignments were performed using ClustalW. Phylogenetic trees were constructed with the maximum likelihood method using IQ-TREE (http://www.iqtree.org) based on aligned amino acid sequences. Numbers on branches are bootstrap values with 1000x resampling.

Ab: *Agkistrodon blomhoffi*, Ac: *Anolis carolinensis*, Af: *Azemiops feae*, Am: *Alligator mississippiensis*, An: *Atheris nitschei*, App: *Agkistrodon piscivorus piscivorus*, As: *Austrelaps superbus*, Bc: *Bungarus candidus*, Bs: *Bothriechis schlegelii*, Ca: *Crotalus atrox*, Cg: *Cerrophidion godmani*, Ch: *Crotalus horridus*, Cr: *Causus rhombeatus*, Clr: *Calloselasma rhodostoma*, Cv: *Crotalus viridis*, Da: *Deinagkistrodon acutus*, Dr:

Daboia russellii, Dt: *Dispholidus typus*, Dv: *Demansia vestigiata*, Ep: *Enhydris polylepis,* Gi: *Gloydius intermediu*s, Hs: *Hoplocephalus stephensii*, Lh: *Lapemis hardwickii,* Lm: *Leioheterodon madagascariensis*, Lp: *Liophis poecilogyrus*, Ls: *Laticauda semifasciata*, Mi: *Micropechis ikaheca*, Na: *Naja atra*, Nk: *Naja kaouthia*, Ns: *Notechis scutatus*, Oh: *Ophiophagus hannah*, Om: *Oxyuranus microlepidotus*, Oo: *Ovophis okinavensis*, Pa: *Pseudechis australis*, Pb: *Python bivittatus*, Pf: *Protobothrops flavoviridis*, Pe: *Protobothrops elegans*, Pm: *Protobothrops mucrosquamatus*, Po: *Philodryas olfersii*, Pp: *Pseudechis porphyriacus*, Pt: *Pseudonaja textilis*, Rn: *Rhinoplocephalus nigrescens*, Rtt: *Rhabdophis tigrinus tigrinus*, Sce: *Sistrurus catenatus edwardsi*, Sct: *Sistrurus catenatus tergeminuss*, Tb: *Trimorphodon biscutatus*, Tc: *Tropidechis carinatus*, Td: *Telescopus dhara*, Tg: *Trimeresurus gracilis*, Tj: *Trimeresurus jerdonii*, Ts: *Thamnophis sirtalis*., Vb: *Vipera berus*, Vn: *Vipera nikolskii*, Vs: *Viridovipera stejnegeri.*

Supplementary Figure S15. Summary and a phylogenetic tree of the vespryn family. SV and NV genes (a) and a molecular phylogeny (b) of the vespryn family. Multiple sequence alignments were performed using ClustalW. Phylogenetic trees were constructed with the maximum likelihood method using IQ-TREE (http://www.iqtree.org) based on aligned amino acid sequences. Numbers on branches are bootstrap values with 1000x resampling.

Ac: *Anolis carolinensis*, Ca: *Crotalus atrox*, Ch: *Crotalus horridus*, Lm: *Leioheterodon madagascariensis*, Mi: *Micropechis ikaheka*, Oh: *Ophiophagus hannah*, Pb: *Python*

bivittatus, Pf: *Protobothrops flavoviridis*, Pm: *Protobothrops mucrosquamatus.*

Supplementary Figure S16. Summary and a phylogenetic tree of the 5'-nucleotidase family. SV and NV genes (a) and a molecular phylogeny (b) of the 5’-nucleaotidase family. Multiple sequence alignments were performed using ClustalW. Phylogenetic trees were constructed with the maximum likelihood method using IQ-TREE (http://www.iqtree.org) based on aligned amino acid sequences. Numbers on branches are bootstrap values with 1000x resampling.

Ac: *Anolis carolinensis*, Oh: *Ophiophagus hannah*, Oo: *Ovophis okinavensis*, Pb: *Python bivittatus*, Pe: *Protobothrops elegans*, Pf: *Protobothrops flavoviridis*, Pm: *Protobothrops mucrosquamatus.*

Supplementary Figure S17. Summary and a phylogenetic tree of the dipeptidyl peptidase (DPP) family. SV and NV genes (a) and a molecular phylogeny (b) of the dipeptidyl peptidase family. Multiple sequence alignments were performed using ClustalW. Phylogenetic trees were constructed with the maximum likelihood method using IQ-TREE (http://www.iqtree.org) based on aligned amino acid sequences. Numbers on branches are bootstrap values with 1000x resampling.

Ac: *Anolis carolinensis*, Am: *Alligator mississippiensis*, As: *Austrelaps superbus*, Gj: *Gekko japonicus*, Ns: *Notechis scutatus*, Oh: *Ophiophagus hannah*, Oo: *Ovophis okinavensis*, Pa: *Pseudechis australis*, Pb: *Python bivittatus*, Pe: *Protobothrops elegans*, Pf: *Protobothrops flavoviridis*, Pm: *Protobothrops mucrosquamatus,* Ts: *Thamnophis*

sirtalis.

Supplementary Figure S18. Summary and a phylogenetic tree of the vascular endothelial growth factor (VEGF)-like protein family. SV and NV genes (a) and a molecular phylogeny (b) of the VEGF family. Multiple sequence alignments were performed using ClustalW. Phylogenetic trees were constructed with the maximum likelihood method using IQ-TREE (http://www.iqtree.org) based on aligned amino acid sequences. Numbers on branches are bootstrap values with 1000x resampling.

Ac: *Anolis carolinensis*, Ap: *Agkistrodon piscivorus*, App: *Agkistrodon piscivorus piscivorus*, Ba: *Bitis arietans*, Be: *Bothrops erythromelas*, Bg: *Bitis gabonica*, Bj: *Bothrops jararaca*, Ca: *Crotalus adamanteus*, Ch: *Crotalus horridus*, Gt: *Gloydius tsushimaensis*, Mf: *Micrurus fulvius*, Mt: *Micrurus tener,* Oh: *Ophiophagus hannah*, Oo: *Ovophis okinavensis*, Pb: *Python bivittatus*, Pe: *Protobothrops elegans*, Pf: *Protobothrops flavoviridis*, Pm: *Protobothrops mucrosquamatus*, Sce: *Sistrurus catenatus edwardsi*, Sct: *Sistrurus catenatus tergeminuss*, Smb: *Sistrurus miliarius barbouri*, Ts: *Thamnophis sirtalis*, Vaa: *Vipera ammodytes ammodytes*.

Supplementary Figure S19. Summary and a phylogenetic tree of the phosphodiesterase (PDE) family. SV and NV genes (a) and a molecular phylogeny (b) of the PDE family. Multiple sequence alignments were performed using ClustalW. Phylogenetic trees were constructed with the maximum likelihood method using IQ-TREE (http://www.iqtree.org) based on aligned amino acid sequences. Numbers on

branches are bootstrap values with 1000x resampling. A red dot shows the branch point between toxic and non-toxic proteins.

Ac: *Anolis carolinensis*, Am: *Alligator mississippiensis*, Ca: *Crotalus adamanteus*, Ch: *Crotalus horridus*, Mf: *Micrurus fulvius*, Mr: *Macropisthodon rudis*, Mt: *Micrurus tener,* Oo: *Ovophis okinavensis*, Pb: *Python bivittatus*, Pe: *Protobothrops elegans*, Pf: *Protobothrops flavoviridis*, Pm: *Protobothrops mucrosquamatus*, Smb: *Sistrurus miliarius barbouri*.

Supplementary Figure S20. Summary and a phylogenetic tree of the phospholipase B (PLB) family. SV and NV genes (a) and a molecular phylogeny (b) of the PLB family. Multiple sequence alignments were performed using ClustalW. Phylogenetic trees were constructed with the maximum likelihood method using IQ-TREE (http://www.iqtree.org) based on aligned amino acid sequences. Numbers on branches are bootstrap values with 1000x resampling.

Ac: *Anolis carolinensis*, Dc: *Drysdalia coronoides*, Oh: *Ophiophagus hannah*, Oo: *Ovophis okinavensis*, Pb: *Python bivittatus*, Pe: *Protobothrops elegans*, Pf: *Protobothrops flavoviridis*, Pm: *Protobothrops mucrosquamatus*.

Supplementary Figure S21. Summary and a phylogenetic tree of the C-type natriuretic peptide (CNP) family. SV and NV genes (a) and a molecular phylogeny (b) of the CNP family. Multiple sequence alignments were performed using ClustalW. Phylogenetic trees were constructed with the maximum likelihood method using

IQ-TREE (http://www.iqtree.org) based on aligned amino acid sequences. Numbers on branches are bootstrap values with 1000x resampling.

Ac: *Anolis carolinensis*, Acc: *Agkistrodon contortrix contortrix*, Am: *Alligator mississippiensis*, Ap: *Agkistrodon piscivorus*, As: *Alligator sinensis*, Ba: *Bitis arietans*, Bi: *Boiga irregularis*, Bj: *Bothrops jararaca*, Bp: *Bothrops pauloensis*, Ca: *Crotalus adamanteus*, Cc: *Cerastes cerastes*, Ch: *Crotalus horridus*, Cm: *Chelonia mydas*, Cr: *Calloselasma rhodostoma*, Dr: *Daboia russelii*, Eo: *Echis ocellatus*, Epl: *Echis pyramidum leakeyi*, Gb: *Gloydius blomhoffii*, Gg: *Gallus gallus*, Gh: *Gloydius halys*, Gi: *Gloydius intermedius*, Gj: *Gekko japonicus*, Lm: *Lachesis muta*, Mg: *Meleagris gallopavo*, Ml: *Macrovipera lebetina*, Oh: *Ophiophagus hannah*, Oo: *Ovophis okinavensis*, Pb: *Python bivittatus*, Pc: *Protobothrops elegans*, Pf: *Protobothrops flavoviridis*, Phm: *Phalotris mertensi*, Pm: *Protobothrops mucrosquamatus*, Sca: *Struthio camelus australis*, Sct: *Sistrurus tergeminus*, Smb: *Sistrurus miliarius barbouri*, Tg: *Trimeresurus gracilis*, Ts: *Thamnophis sirtalis*.

Supplementary Figure S22. Summary and a phylogenetic tree of the glutaminyl peptide cyclotransferase (GPCase) family. SV and NV genes (a) and a molecular phylogeny (b) of the GPCase family. Multiple sequence alignments were performed using ClustalW. Phylogenetic trees were constructed with the maximum likelihood method using IQ-TREE (http://www.iqtree.org) based on aligned amino acid sequences. Numbers on branches are bootstrap values with 1000x resampling.

Ac: *Anolis carolinensis*, Bd: *Bothrops diporus*, Bi: *Boiga irregularis*, Bj: *Bothrops*

jararaca, Cg: *Cerrophidion godmani*, Cpb: *Chrysemys picta bellii*, Dr: *Daboia russelii*, Gj: *Gekko japonicus*, Mf: *Micrurus fulvius*, Oo: *Ovophis okinavensis*, Pb: *Python bivittatus*, Pe: *Protobothrops elegans*, Pf: *Protobothrops flavoviridis*, Pm: *Protobothrops mucrosquamatus*. Sct: *Sistrurus catenatus tergeminus*, Tg: *Trimeresurus gracilis*, Ts: *Thamnophis sirtalis*.

Supplementary Figure S1.

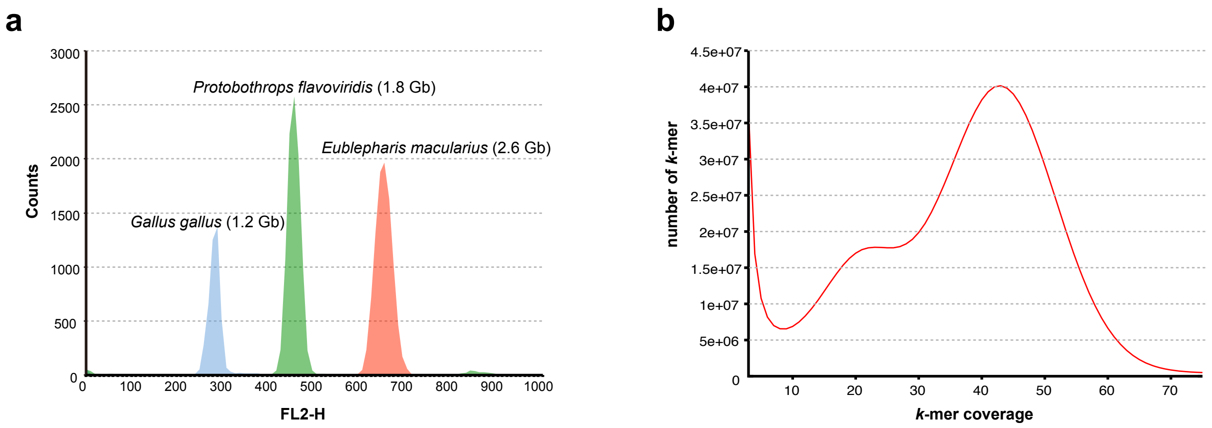

Supplementary Fig. S2.

(a) Hyaluronidase (Hyal)

Member of the family

Gene	Name	Locus	Gene model ID	Transcripts
Hyal01 (svHyal)	svHyaluronidase (svHYAL)	habu1_scaffold7188 : 185477 ... 226495 : -	habu1_s7188_g17820	pb016481_c260499_f1p0_1519
Hyal02 (nvHyal)	hyaluronidase-1 (HYAL1)	habu1_scaffold2564 : 518042 ... 530062 : +	habu1_s2564_g09254	habu1_s2564_g09254.t1
Hyal03 (nvHyal)	hyaluronidase-2 (HYAL2)	habu1_scaffold2564 : 478562 ... 496616 : +	habu1_s2564_g09252	habu1_s2564_g09252.t1
Hyal04 (nvHyal)	hyaluronidase-2 (HYAL2)	habu1_scaffold6849 : 149616 ... 194647 : +	habu1_s6849_g17560	habu1_s6849_g17560.t1
Hyal05 (nvHyal)	hyaluronidase-3 (HYAL3)	habu1_scaffold2564 : 546642 ... 569966 : +	habu1_s2564_g09256	habu1_s2564_g09256.t1
Hyal06 (nvHyal)	hyaluronidase-4 (HYAL4)	habu1_scaffold7188 : 99577 ... 184945 : -	habu1_s7188_g17819	habu1_s7188_g17819.t1

Molecular phylogeny of members

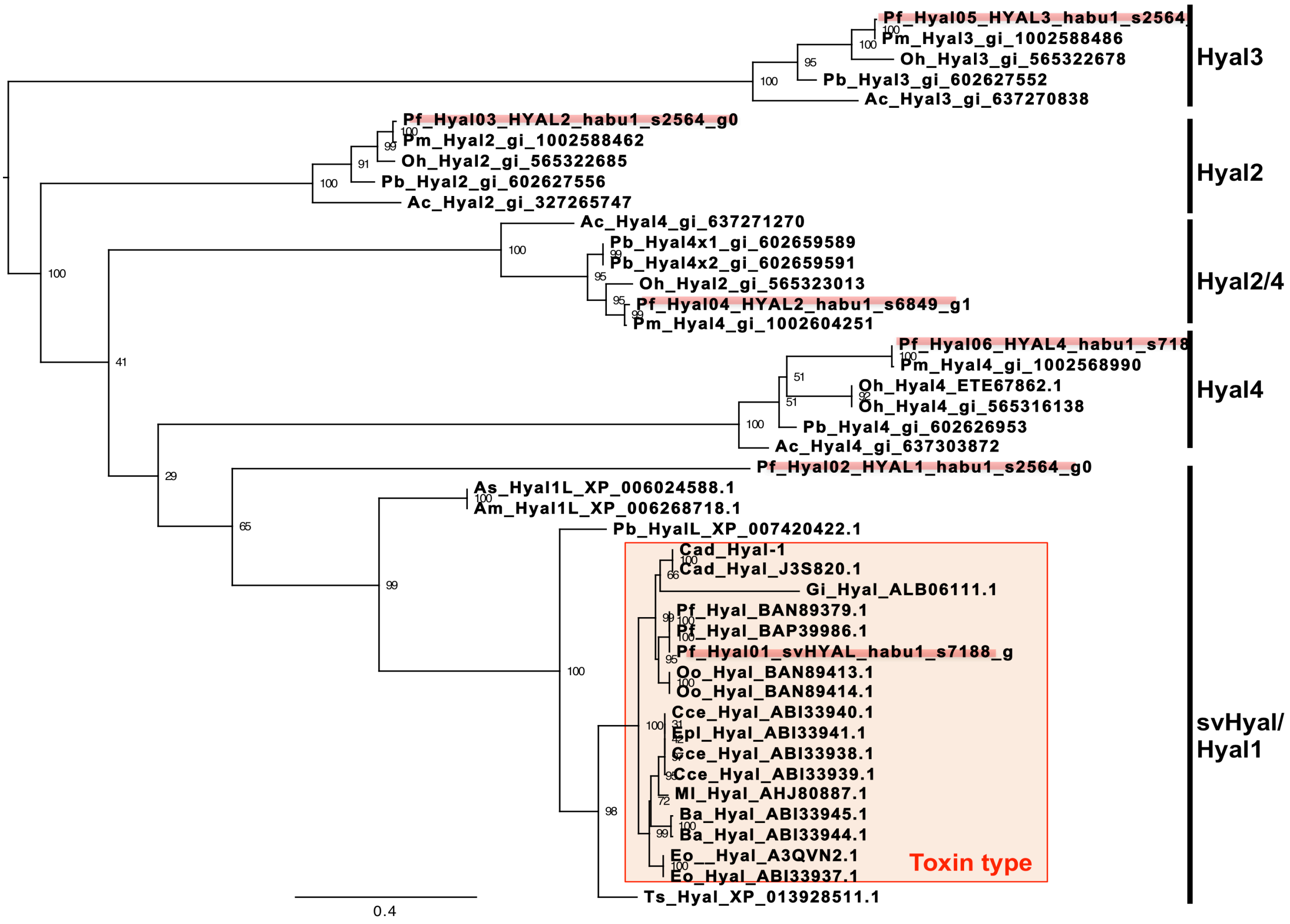

Fig. S2 contineued.

(b) Nerve growth factor (NGF)

Member of the family

Gene	Name	Locus	Gene model ID	Transcripts
Neu01 (svNeu)	svNGF	habu1_scaffold3536 : 227336 ... 309758 : +	habu1_s3536_g11975	pb016481_c100488_f2p5_1112
Neu02 (nvNeu)	BDNF	habu1_scaffold3803 : 208016 ... 266737 : +	habu1_s3803_g12736	habu1_s3803_g12736.t1
Neu03 (nvNeu)	neurotrophin-3 isoform	habu1_scaffold105 : 263936 ... 278118 : +	habu1_s105_g00548	habu1_s105_g00548.t1
Neu04 (nvNeu)	neurotrophin-4 isoform	habu1_scaffold6123 : 46517 ... 70395 : -	habu1_s6123_g16797	habu1_s6123_g16797.t1

Molecular phylogeny of members

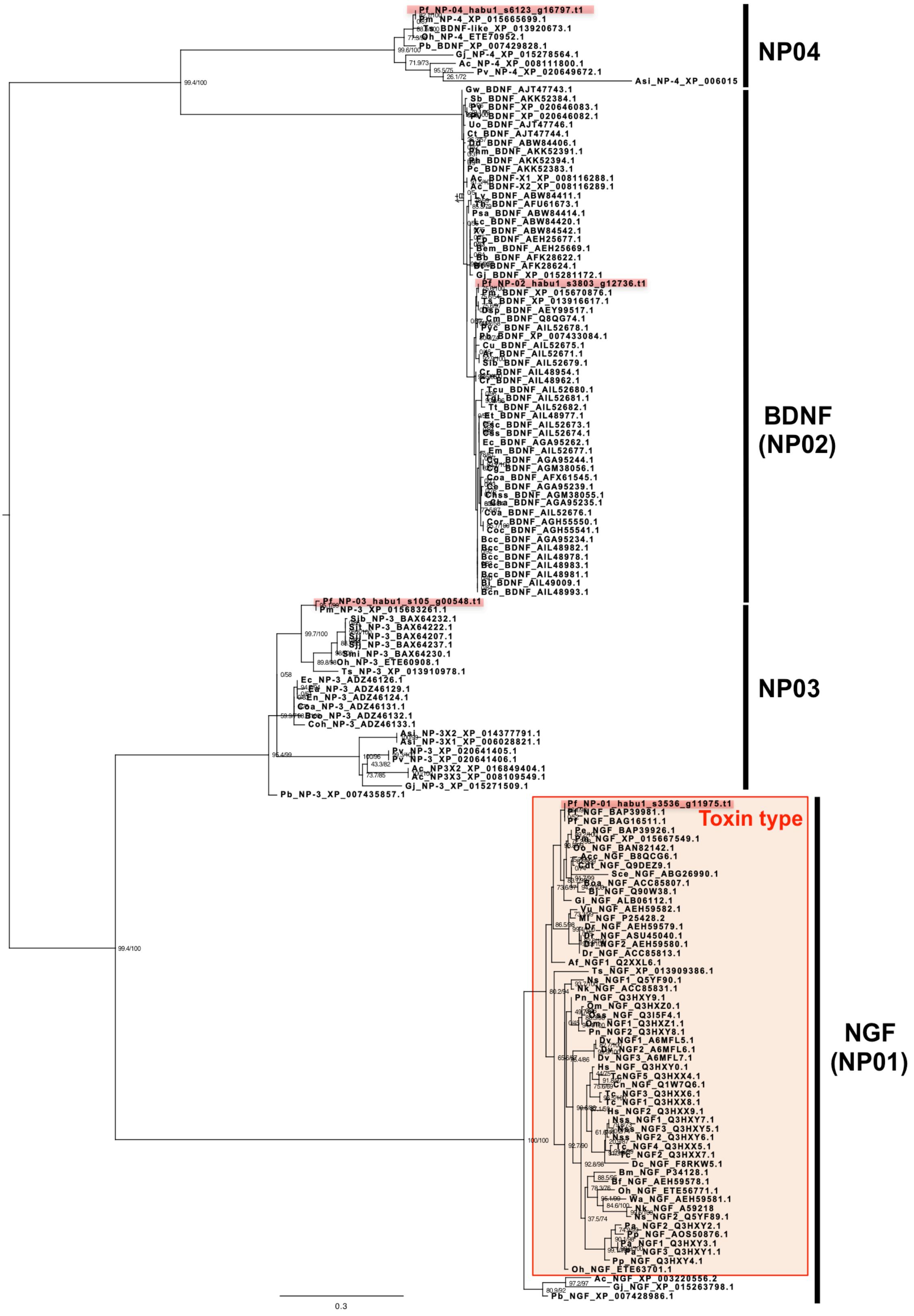

Supplementary Figure S2 contineued.

(c) L-amino acid oxidase (LAAO)

Member of the family

Gene	Name	Locus	Gene Model ID	Transcripts
LAAO01 (svLAAO)	LAAO01 (svLAAO)	habu1_scaffold402940 : 463276 ... 513813 : -	habu1_s402940_g24950	pb016481_c420538_f1p26_2821
LAAO02 (nvLAAO)	LAAO02 (nvLAAO)	habu1_scaffold402940 : 432924 ... 460003 : -	habu1_s402940_g24949	habu1_s402940_g24949.t1
LAAO03 (nvLAAO)	LAAO03 (nvLAAO)	habu1_scaffold402940 : 288786 ... 347897 : +	habu1_s402940_g24947b	habu1_s402940_g24947.t1

Molecular phylogeny of members

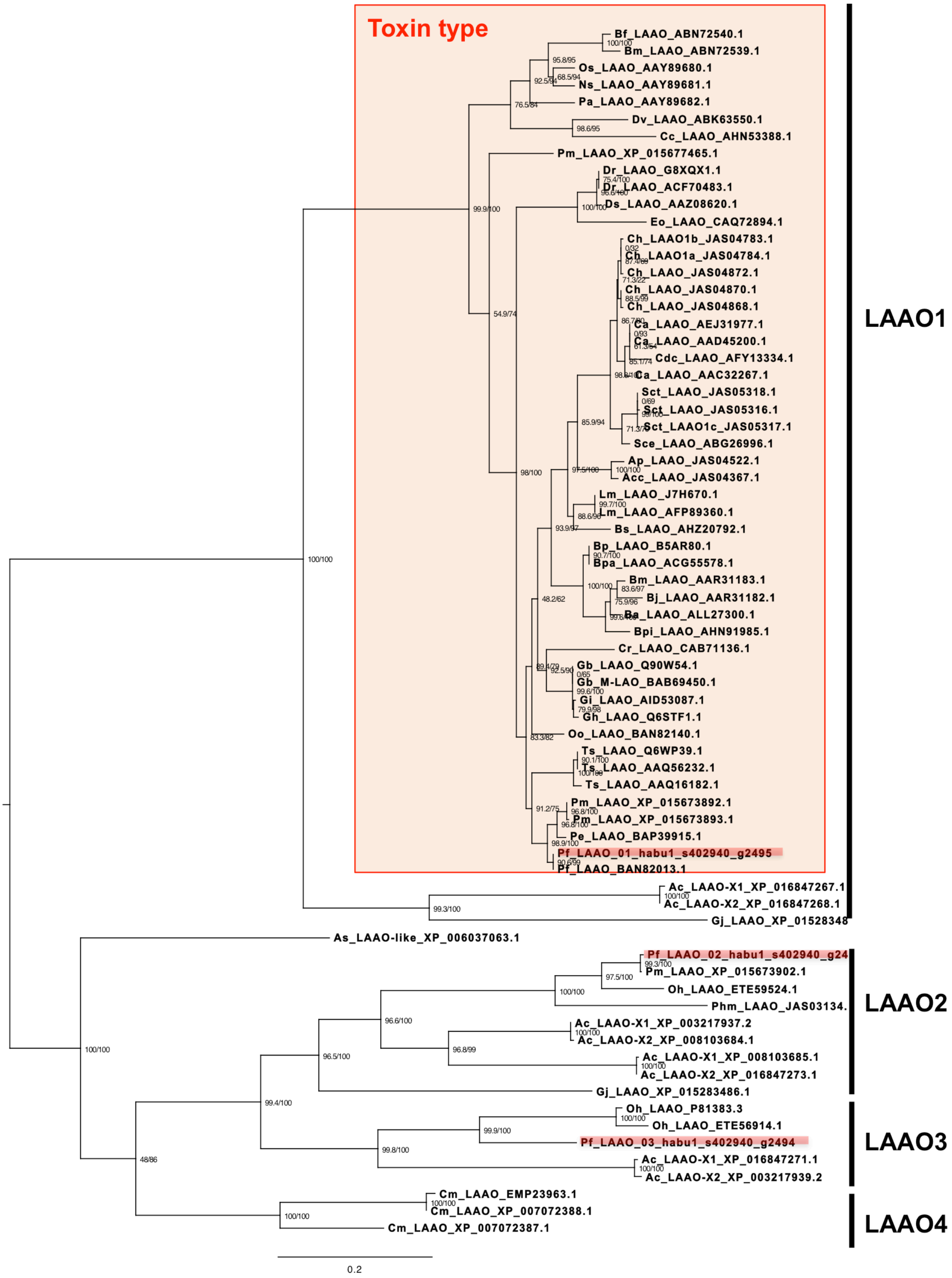

Supplementary Figure S3.

(a) SV genes

Gene	Name	Locus	Gene model ID	Transcripts
svMP01	HV1, Vascular apoptosis-inducing protein	habu1_scaffold2862: 139192-161910: +	habu1_s2862_g10314a	pb016480_c42639_f26p8_2346
svMP02	flavorase	habu1_scaffold2862: 165955-186529: +	habu1_s2862_g10314b	pb016480_c42674_f12p2_2354
svMP03	VMP-III -like	habu1_scaffold2862: 188915-209119: +,<br>habu1_scaffold14911:15918-22474: +	habu1_s2862_g10314c	pb016481_c551930_f88p36_2337
svMP04	jerdonitin-like	habu1_scaffold3258:1...15093: +,<br>habu1_scaffold415864:1...818: -,<br>habu1_scaffold191139: 1...797: -,<br>habu1_scaffold403873:1...701: -	habu1_s3258_g11210	pb016481_c222387_f3p5_2004
svMP05	HR1a	habu1_scaffold3258: 15841-52645: +	habu1_s3258_g11211	pb016480_c115296_f1p1_2355
svMP06	H2 metalloproteinase (metalloprotease P-Iia)	habu1_scaffold14911:2022...5557: +	habu1_s14911_g21429	pb016481_c225249_f1p9_1815
svMP07	HR2a	habu1_scaffold14911: 60789...76673: +<br>habu1_scaffold14911: 1...21550: +	habu1_s14911_g21430a	pb016480_c226_f26p9_2010
svMP08	flavoridin	habu1_scaffold14911:3733...53141: +	habu1_s14911_g21430b	pb016480_c222_f46p26_1997
svMP09	HR1b	habu1_scaffold399953:20785...36711: -	habu1_s399953_g24864a	pb016481_c917376_f5p9_2037
svMP10	Mt-b/ elegantin-like	habu1_scaffold399953:1...38225: -<br>habu1_scaffold410279:1...747: -<br>habu1_scaffold408733:1...51: -<br>habu1_scaffold412967:1...795: -	habu1_s399953_g24864b	pb016481_c16287_f13p13_2020
svMP11	NaMP	habu1_scaffold2862: 106532…130000+	habu1_s2862_g10314d	habu1_s2862_g10314.t2

(b) NV genes

Gene	Name	Locus	Gene model ID	Transcripts
nvMP01	ADAM8/9-like	habu1_scaffold2 : 71286 ... 110080 : +	habu1_s2_g00003	habu1_s2_g00003.t1
nvMP02	ADAM9	habu1_scaffold179838 : 1 ... 914 : +	habu1_s179838_g24144	habu1_s179838_g24144.t1
nvMP03	ADAM 9	habu1_scaffold2858 : 1807398 ... 1833873 : +	habu1_s2858_g10274	habu1_s2858_g10274.t1
nvMP04	ADAM 10-like	habu1_scaffold2336: 1112623...1112538: -,<br>habu1_scaffold4524 : 1 ... 13872 : +,<br>habu1_scaffold333519 : 1 ... 395 : -,<br>habu1_scaffold2934 : 1 ... 18140 : +,<br>habu1_scaffold244794:1..917-	habu1_s4524_g14387/<br>habu1_s333519_g24419/<br>habu1_s2934_g10526	habu1_s2934_g10526.t1
nvMP05	ADAM10	habu1_scaffold878 : 135568 ... 161715 : -	habu1_s878_g03107	habu1_s878_g03107.t1
nvMP06	ADAM11	habu1_scaffold2140 : 97648 ... 117201 : -	habu1_s2140_g07747	habu1_s2140_g07747.t1
nvMP07	ADAM11	habu1_scaffold5075 : 1 ... 61735 : -	habu1_s5075_g15185	habu1_s5075_g15185.t1
nvMP08	ADAM 12	habu1_scaffold2690 : 97936 ... 361842 : +	habu1_s2690_g09595	habu1_s2690_g09595.t1
nvMP09	ADAM 15	habu1_scaffold5687 : 27897 ... 62375 : -	habu1_s5687_g16173	habu1_s5687_g16173.t1
nvMP10	ADAM 17	habu1_scaffold602 : 44893 ... 82435 : -	habu1_s602_g02843	habu1_s602_g02843.t1
nvMP11	ADAM 19	habu1_scaffold5506 : 105086 ... 193255 : -	habu1_s5506_g15874	habu1_s5506_g15874.t1
nvMP12	ADAM 20	(habu1_scaffold8405:494795..504794: +)		
nvMP13	ADAM21	habu1_scaffold399842 : 646178 ... 651147 : +	habu1_s399842_g24578	habu1_s399842_g24578.t1
nvMP14	ADAM 22	habu1_scaffold4012 : 359513 ... 383057 : -	habu1_s4012_g13214	habu1_s4012_g13214.t1
nvMP15	ADAM 32	habu1_scaffold2858 : 1837688 ... 1857057 : +	habu1_s2858_g10275	habu1_s2858_g10275.t1
nvMP16	ADAM 33	habu1_scaffold3839 : 5573 ... 17043 : -	habu1_s3839_g12793	habu1_s3839_g12793.t1
nvMP17	ADAM 33	habu1_scaffold44599 : 1 ... 27737 : +	habu1_s44599_g23329	habu1_s44599_g23329.t1
nvMP18	ADAMTS-like	habu1_scaffold5048 : 540792 ... 774172 : -	habu1_s5048_g15167	habu1_s5048_g15167.t1
nvMP19	ADAMTS1	habu1_scaffold2614 : 247016 ... 269192 : +	habu1_s2614_g09476	habu1_s2614_g09476.t1
nvMP20	ADAMTS 2	habu1_scaffold5378 : 27996 ... 91121 : +	habu1_s5378_g15691	habu1_s5378_g15691.t1
nvMP21	ADAMTS2	habu1_scaffold4909 : 1 ... 71717 : +	habu1_s4909_g14946	habu1_s4909_g14946.t1
nvMP22	ADAMTS2	habu1_scaffold4909 : 72026 ... 141808 : +	habu1_s4909_g14947	habu1_s4909_g14947.t1
nvMP23	ADAMTS 3	habu1_scaffold3496 : 105170 ... 245955 : -	habu1_s3496_g11873	habu1_s3496_g11873.t1
nvMP24	ADAMTS3	habu1_scaffold2091 : 29366 ... 227749 : +	habu1_s2091_g07645	habu1_s2091_g07645.t1
nvMP25	ADAMTS 4-like	habu1_scaffold2369 : 17219 ... 58128 : -	habu1_s2369_g08591	habu1_s2369_g08591.t1
nvMP26	ADAMTS4	habu1_scaffold121 : 92416 ... 110788 : +	habu1_s121_g00604	habu1_s121_g00604.t1
nvMP27	ADAMTS5-like	habu1_scaffold6969:1..5593: +,<br>habu1_scaffold6970 : 1 ... 21977 : +	habu1_s6970_g17669	habu1_s6970_g17669.t1
nvMP28	ADAMTS5	habu1_scaffold2614 : 187286 ... 229869 : +	habu1_s2614_g09475	habu1_s2614_g09475.t1
nvMP29	ADAMTS6	habu1_scaffold16578 : 176146 ... 270945 : -	habu1_s16578_g21764	habu1_s16578_g21764.t1
nvMP30	ADAMTS7	habu1_scaffold1390 : 1630741 ... 1657463 : -	habu1_s1390_g05010	habu1_s1390_g05010.t1
nvMP31	ADAMTS 8	habu1_scaffold2647 : 240949 ... 299435 : -	habu1_s2647_g09516	habu1_s2647_g09516.t1
nvMP32	ADAMTS9	habu1_scaffold523 : 106186 ... 238219 : +	habu1_s523_g02405	habu1_s523_g02405.t1
nvMP33	ADAMTS9-like	habu1_scaffold524 : 46586 ... 55632 : +	habu1_s524_g02406	habu1_s524_g02406.t1
nvMP34	ADAMTS10	habu1_scaffold2275 : 129686 ... 195770 : +	habu1_s2275_g08137	habu1_s2275_g08137.t1
nvMP35	ADAMTS12-like	habu1_scaffold2255 : 1335208 ... 1356653 : -	habu1_s2255_g08060	habu1_s2255_g08060.t1
nvMP36	ADAMTS12	habu1_scaffold2256 : 1 ... 12075 : -	habu1_s2256_g08061	habu1_s2256_g08061.t1
nvMP37	ADAMTS13	habu1_scaffold1917 : 171206 ... 197845 : -	habu1_s1917_g07045	habu1_s1917_g07045.t1
nvMP38	ADAMTS14	habu1_scaffold5729 : 315496 ... 387476 : +	habu1_s5729_g16218	habu1_s5729_g16218.t1
nvMP39	ADAMTS15	habu1_scaffold2647 : 327316 ... 360932 : +	habu1_s2647_g09517	habu1_s2647_g09517.t1
nvMP40	ADAMTS16	habu1_scaffold17754 : 22336 ... 103527 : +	habu1_s17754_g21974	habu1_s17754_g21974.t1
nvMP41	ADAMTS17	habu1_scaffold215 : 1777532 ... 1900885 : +	habu1_s215_g01205	habu1_s215_g01205.t1
nvMP42	ADAMTS18	habu1_scaffold496 : 265045 ... 629934 : -	habu1_s496_g02284	habu1_s496_g02284.t1
nvMP43	ADAMTS19	habu1_scaffold83 : 129465 ... 215485 : -	habu1_s83_g00469	habu1_s83_g00469.t1
nvMP44	MMP-14	habu1_scaffold2519 : 40687 ... 63855 : -	habu1_s2519_g09165	habu1_s2519_g09165.t1
nvMP45	MMP-15	habu1_scaffold2277 : 991963 ... 999434 : +	habu1_s2277_g08190	habu1_s2277_g08190.t1
nvMP46	MMP-16	habu1_scaffold2447 : 249336 ... 445542 : +	habu1_s2447_g08959	habu1_s2447_g08959.t1
nvMP47	MMP-17	habu1_scaffold2313 : 1036 ... 67938 : +	habu1_s2313_g08399	habu1_s2313_g08399.t1
nvMP48	MMP-17	habu1_scaffold1466 : 578466 ... 598489 : -	habu1_s1466_g05412	habu1_s1466_g05412.t1
nvMP49	MMP-19	habu1_scaffold402967 : 95446 ... 134769 : +	habu1_s402967_g25016	habu1_s402967_g25016.t1
nvMP50	MMP-20	habu1_scaffold532 : 778467 ... 796669 : -	habu1_s532_g02441	habu1_s532_g02441.t1
nvMP51	MMP-24	habu1_scaffold4809 : 306319 ... 347795 : -	habu1_s4809_g14788	habu1_s4809_g14788.t1
nvMP52	MMP-25	habu1_scaffold9747 : 1 ... 14152 : +	habu1_s9747_g19608	habu1_s9747_g19608.t1
nvMP53	MMP-25	habu1_scaffold21648 : 40170 ... 53088 : -	habu1_s21648_g22429	habu1_s21648_g22429.t1
nvMP54	MMP-28	habu1_scaffold1501 : 177931 ... 192935 : -	habu1_s1501_g05556	habu1_s1501_g05556.t1
nvMP55	ATP-dependent zinc metalloprotease YME1L1	habu1_scaffold403007 : 90596 ... 121556 : +	habu1_s403007_g25105	habu1_s403007_g25105.t1
nvMP56	Metalloprotease TIKI2	habu1_scaffold13164 : 180021 ... 242825 : -	habu1_s13164_g20985	habu1_s13164_g20985.t1
nvMP57	MPtypeIII-1	habu1_scaffold2862: 26906…90317:+	habu1_s2862_g10314e	habu1_s2862_g10314.t1

(c)

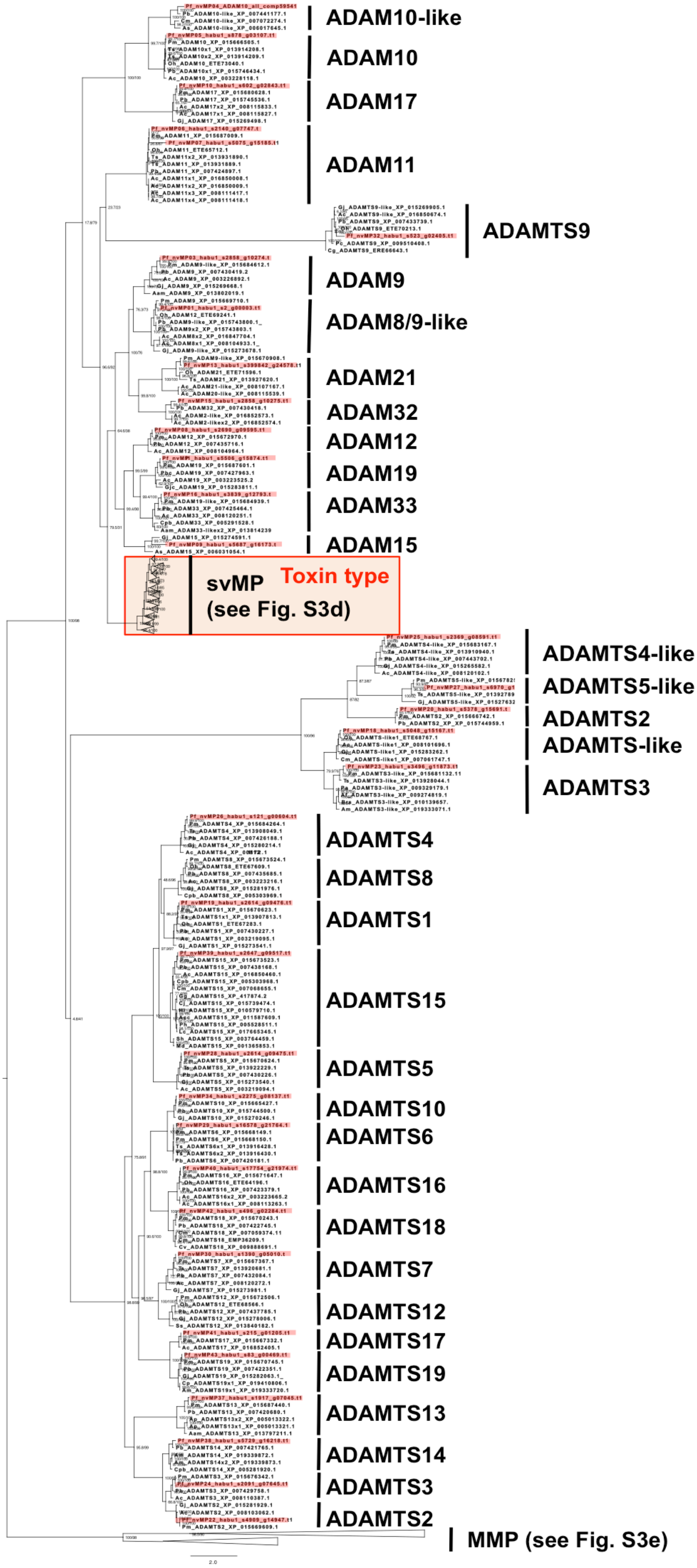
ADAM10-like
ADAM10
ADAM17
ADAM11
ADAMTS9
ADAM9
ADAM8/9-like
ADAM21
ADAM32
ADAM12
ADAM19
ADAM33
ADAM15
svMP
(see Fig. S3d)
Toxin type
ADAMTS4-like
ADAMTS5-like
ADAMTS2
ADAMTS-like
ADAMTS3
ADAMTS4
ADAMTS8
ADAMTS1
ADAMTS15
ADAMTS5
ADAMTS10
ADAMTS6
ADAMTS16
ADAMTS18
ADAMTS7
ADAMTS12
ADAMTS17
ADAMTS19
ADAMTS13
ADAMTS14
ADAMTS3
ADAMTS2
MMP (see Fig. S3e)

(d)

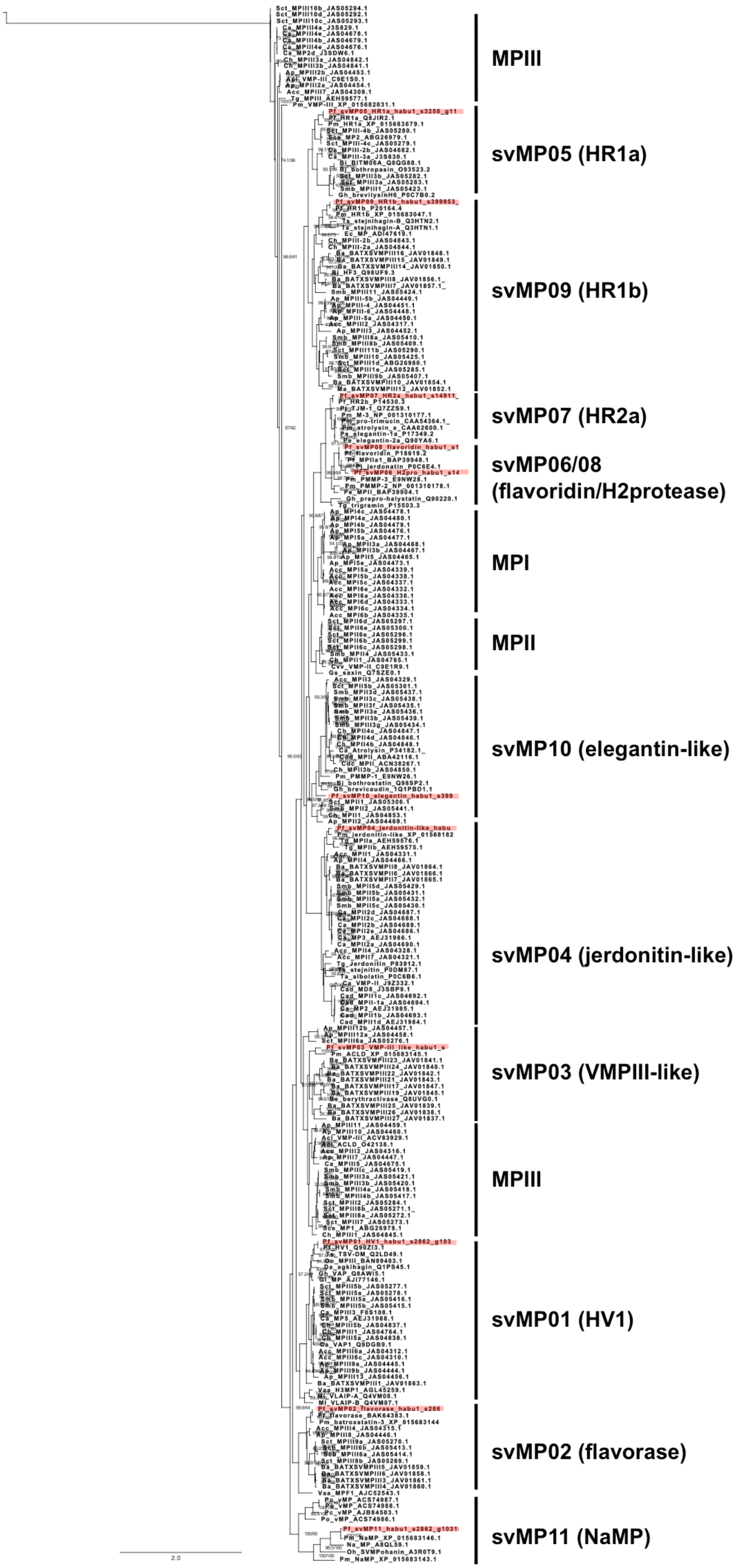
MPIII
svMP05 (HR1a)
svMP09 (HR1b)
svMP07 (HR2a)
svMP06/08
(flavoridin/H2protease)
MPI
MPII
svMP10 (elegantin-like)
svMP04 (jerdonitin-like)
svMP03 (VMPIII-like)
MPIII
svMP01 (HV1)
svMP02 (flavorase)
svMP11 (NaMP)
2.0

(e)

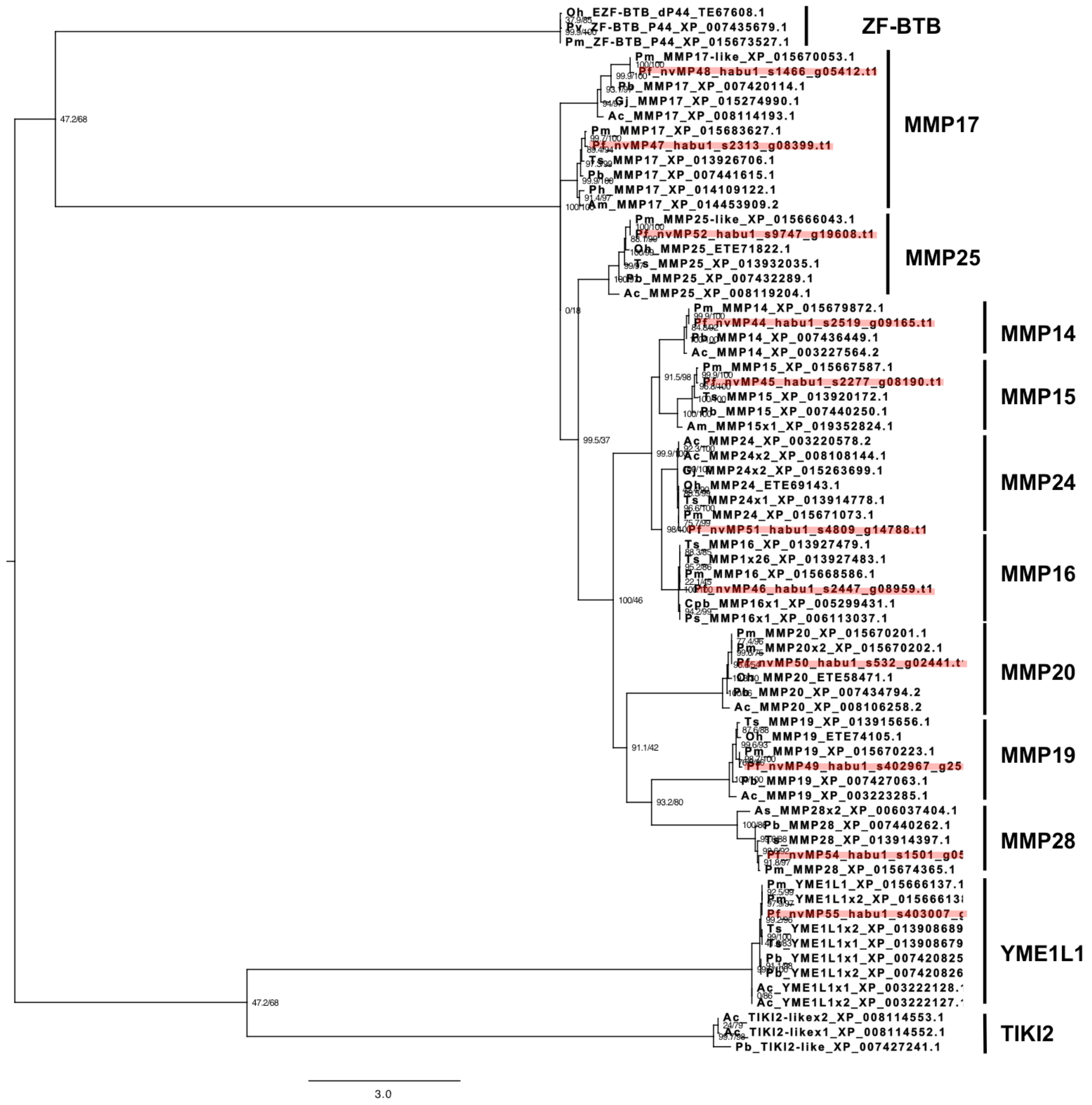

Oh_EZF-BTB_dP44_TE67608.1
Pv_ZF-BTB_P44_XP_007435679.1
Pm_ZF-BTB_P44_XP_015673527.1
ZF-BTB
Pm_MMP17-like_XP_015670053.1
Pf_nvMP48_habu1_s1466_g05412.t1
Pb_MMP17_XP_007420114.1
Gj_MMP17_XP_015274990.1
Ac_MMP17_XP_008114193.1
Pm_MMP17_XP_015683627.1
Pf_nvMP47_habu1_s2313_g08399.t1
Ts_MMP17_XP_013926706.1
Pb_MMP17_XP_007441615.1
Ph_MMP17_XP_014109122.1
Am_MMP17_XP_014453909.2
MMP17
Pm_MMP25-like_XP_015666043.1
Pf_nvMP52_habu1_s9747_g19608.t1
Oh_MMP25_ETE71822.1
Ts_MMP25_XP_013932035.1
Pb_MMP25_XP_007432289.1
Ac_MMP25_XP_008119204.1
MMP25
Pm_MMP14_XP_015679872.1
Pf_nvMP44_habu1_s2519_g09165.t1
Pb_MMP14_XP_007436449.1
Ac_MMP14_XP_003227564.2
MMP14
Pm_MMP15_XP_015667587.1
Pf_nvMP45_habu1_s2277_g08190.t1
Ts_MMP15_XP_013920172.1
Pb_MMP15_XP_007440250.1
Am_MMP15x1_XP_019352824.1
MMP15
Ac_MMP24_XP_003220578.2
Ac_MMP24x2_XP_008108144.1
Gj_MMP24x2_XP_015263699.1
Oh_MMP24_ETE69143.1
Ts_MMP24x1_XP_013914778.1
Pm_MMP24_XP_015671073.1
Pf_nvMP51_habu1_s4809_g14788.t1
MMP24
Ts_MMP16_XP_013927479.1
Ts_MMP1x26_XP_013927483.1
Pm_MMP16_XP_015668586.1
Pf_nvMP46_habu1_s2447_g08959.t1
Cpb_MMP16x1_XP_005299431.1
Ps_MMP16x1_XP_006113037.1
MMP16
Pm_MMP20_XP_015670201.1
Pm_MMP20x2_XP_015670202.1
Pf_nvMP50_habu1_s532_g02441.t
Oh_MMP20_ETE58471.1
Pb_MMP20_XP_007434794.2
Ac_MMP20_XP_008106258.2
MMP20
Ts_MMP19_XP_013915656.1
Oh_MMP19_ETE74105.1
Pm_MMP19_XP_015670223.1
Pf_nvMP49_habu1_s402967_g25
Pb_MMP19_XP_007427063.1
Ac_MMP19_XP_003223285.1
MMP19
As_MMP28x2_XP_006037404.1
Pb_MMP28_XP_007440262.1
Ts_MMP28_XP_013914397.1
Pf_nvMP54_habu1_s1501_g05
Pm_MMP28_XP_015674365.1
MMP28
Pm_YME1L1_XP_015666137.1
Pm_YME1L1x2_XP_01566613
Pf_nvMP55_habu1_s403007_
Ts_YME1L1x2_XP_013908689
Ts_YME1L1x1_XP_013908679
Pb_YME1L1x1_XP_007420825
Pb_YME1L1x2_XP_007420826
Ac_YME1L1x1_XP_003222128.
Ac_YME1L1x2_XP_003222127.
YME1L1
Ac_TIKI2-likex2_XP_008114553.1
Ac_TIKI2-likex1_XP_008114552.1
Pb_TIKI2-like_XP_007427241.1
TIKI2
47.2/68
0/18
99.5/37
100/46
91.1/42
93.2/80
91.5/98
99.9/100
47.2/68
3.0

Supplementary Figure S4.

(a) SV genes

Gene	Name	Locus	Gene model ID	Transcripts
svSP01	TLf1/flavoxobin	habu1_scaffold7597 : 107550 ... 117068 : -	habu1_s7597_g18190a	pb016481_c628_f19p22_1897
svSP02	TLf2	habu1_scaffold409039:463...626: -<br>habu1_scaffold189200:1..2644: +<br>habu1_scaffold142871:1..912: +		pb016481_c385685_f143p90_3720
svSP03	TLf3	habu1_scaffold7597 : 146701 ... 152624 : -	habu1_s7597_g18190b	pb016481_c663_f11p12_1578
svSP04		habu1_scaffold6789 : 134126 ... 143039 : +	habu1_s6789_g17480	pb016480_c50972_f1p2_1888
svSP05		habu1_scaffold6789: 168298...179481: +	habu1_s6789_g17481a	pb016481_c237569_f1p19_1959
svSP06		habu1_scaffold4106 : 1768 ... 14932 : +,<br>habu1_scaffold375860:1..711: +,<br>habu1_scaffold50513:1..3838: +,<br>habu1_scaffold73303:1..843: +,<br>habu1_scaffold270900:1..692: +	habu1_s4106_g13431a	pb016481_c782298_f2p3_6239
svSP07		habu1_scaffold4106 : 20911 ... 28186: +	habu1_s4106_g13431b	pb016481_c395211_f1p12_2348
svSP08		habu1_scaffold6789 : 187000 ... 207128 : +	habu1_s6789_g17481b	pb016481_c1327_f9p6_1578
svSP09		habu1_scaffold7597 : 96885 ... 155905 : -	habu1_s7597_g18190c	pb016481_c243922_f1p5_2691
svSP10		habu1_scaffold4106 : 28886 ... 46059 : +	habu1_s4106_g13432	pb016481_c944_f4p2_1299
svSP11	Jerdonobin-II like, GPV-PA	habu1_scaffold22023 : 12371 ... 34705 : -	habu1_s22023_g22469	pb016481_c591372_f1p3_1420

(b) NV genes

Gene	Name	Locus	Gene Model ID	Transcripts
nvSP01	anionic trypsin-like	habu1_scaffold2510 : 115346 ... 122903 : +	habu1_s2510_g09123	habu1_s2510_g09123.t1
nvSP02	anionic trypsin-2-like	habu1_scaffold2510 : 78736 ... 98860 : +	habu1_s2510_g09122	habu1_s2510_g09122.t1
nvSP03	trypsin I-P38-like	habu1_scaffold2510 : 68775 ... 74705 : -	habu1_s2510_g09121	habu1_s2510_g09121.t1
nvSP04	kallikrein-14-like	habu1_scaffold9123 : 219136 ... 242356 : +	habu1_s9123_g19184	habu1_s9123_g19184.t1
nvSP05	trypsin-like	habu1_scaffold9123 : 194396 ... 219087 : +	habu1_s9123_g19183	habu1_s9123_g19183.t1
nvSP06	chymotrypsin-C	habu1_scaffold9572 : 83215 ... 86125 : -	habu1_s9572_g19445	habu1_s9572_g19445.t1
nvSP07	chymotrypsin-like elastase family member 3B	habu1_scaffold3296 : 1 ... 6460 : +	habu1_s3296_g11282	habu1_s3296_g11282.t1
nvSP08	chymotrypsin-like protease CTRL-1	habu1_scaffold493 : 678049 ... 684597 : -	habu1_s493_g02255	habu1_s493_g02255.t1
nvSP09	Neurotrypsin	habu1_scaffold1537 : 430486 ... 473512 : +	habu1_s1537_g05780	habu1_s1537_g05780.t1
nvSP10	neurotrypsin-like	habu1_scaffold2696 : 320034 ... 343955 : -	habu1_s2696_g09609	habu1_s2696_g09609.t1
nvSP11	thrombin	habu1_scaffold10983 : 253158 ... 268223 : -<br>/habu1_scaffold10995 : 15124 ... 44505 : -	habu1_s10983_g20317	habu1_s10983_g20317.t1
nvSP12	tissue-type plasminogen activator (tPA)	habu1_scaffold2858 : 1135301 ... 1175837 : -	habu1_s2858_g10253	habu1_s2858_g10253.t1
nvSP13	plasma kallikrein	habu1_scaffold11004 : 283304 ... 308535 : -	habu1_s11004_g20335	habu1_s11004_g20335.t1
nvSP14	Inactive serine protease 35	habu1_scaffold1630 : 31548 ... 63325 : -	habu1_s1630_g06161	habu1_s1630_g06161.t1
nvSP15	inactive serine protease PAMR1-like	habu1_scaffold3643 : 56659 ... 132295 : -	habu1_s3643_g12251	habu1_s3643_g12251.t1
nvSP16	Serine protease 23, partial	habu1_scaffold6061 : 41736 ... 53088 : +	habu1_s6061_g16640	habu1_s6061_g16640.t1
nvSP17	serine protease 27-like	habu1_scaffold13321 : 1 ... 17475 : -	habu1_s13321_g21080	habu1_s13321_g21080.t1
nvSP18	serine protease 27-like	habu1_scaffold2928 : 8492 ... 20060 : +	habu1_s2928_g10510	habu1_s2928_g10510.t1
nvSP19	serine protease 27-like	habu1_scaffold661 : 20981 ... 65935 : -	habu1_s661_g03024	habu1_s661_g03024.t1
nvSP20	serine protease 27-like	habu1_scaffold14979 : 1 ... 65141 : -	habu1_s14979_g21447	habu1_s14979_g21447.t1
nvSP21	serine protease 33-like	habu1_scaffold661 : 80056 ... 108788 : +	habu1_s661_g03025	habu1_s661_g03025.t1
nvSP22	transmembrane protease serine 12-like: partial	habu1_scaffold5131 : 399731 ... 416735 : -	habu1_s5131_g15292	habu1_s5131_g15292.t1
nvSP23	serine protease 53	habu1_scaffold3327 : 161929 ... 190072 : -	habu1_s3327_g11330	habu1_s3327_g11330.t1
nvSP24	serine protease 55-like	habu1_scaffold16606 : 34165 ... 36715 : -	habu1_s16606_g21794	habu1_s16606_g21794.t1
nvSP25	serine protease 56	habu1_scaffold2472 : 86176 ... 122259 : +	habu1_s2472_g09051	habu1_s2472_g09051.t1
nvSP26	serine protease HTRA4	habu1_scaffold2861 : 833394 ... 841595 : +	habu1_s2861_g10303	habu1_s2861_g10303.t1
nvSP27	serine protease HTRA1-like	habu1_scaffold1135 : 72756 ... 111731 : +	habu1_s1135_g03965	habu1_s1135_g03965.t1
nvSP28	serine protease HTRA2, mitochondrial-like	habu1_scaffold2921 : 104599 ... 106495 : -	habu1_s2921_g10480	habu1_s2921_g10480.t1
nvSP29	thymus-specific serine protease-like	habu1_scaffold1205 : 38676 ... 53312 : +	habu1_s1205_g04155	habu1_s1205_g04155.t1
nvSP30	mannan-binding lectin serine protease 1-like: partial	habu1_scaffold76496 : 1 ... 797 : +	habu1_s76496_g23722	habu1_s76496_g23722.t1
nvSP31	mannan-binding lectin serine protease 1 :partial	habu1_scaffold120144 : 1 ... 3501 : +	habu1_s120144_g23985	habu1_s120144_g23985.t1
nvSP32	mannan-binding lectin serine protease 2	habu1_scaffold1702 : 13930 ... 24737 : -	habu1_s1702_g06326	habu1_s1702_g06326.t1
nvSP33	mannan-binding lectin serine protease 2	habu1_scaffold1703 : 1 ... 17225 : -	habu1_s1703_g06327	habu1_s1703_g06327.t1
nvSP34	serine protease 27-like	habu1_scaffold14979 : 1 ... 65141 : -	habu1_s14979_g21447	habu1_s14979_g21447.t1

(c)

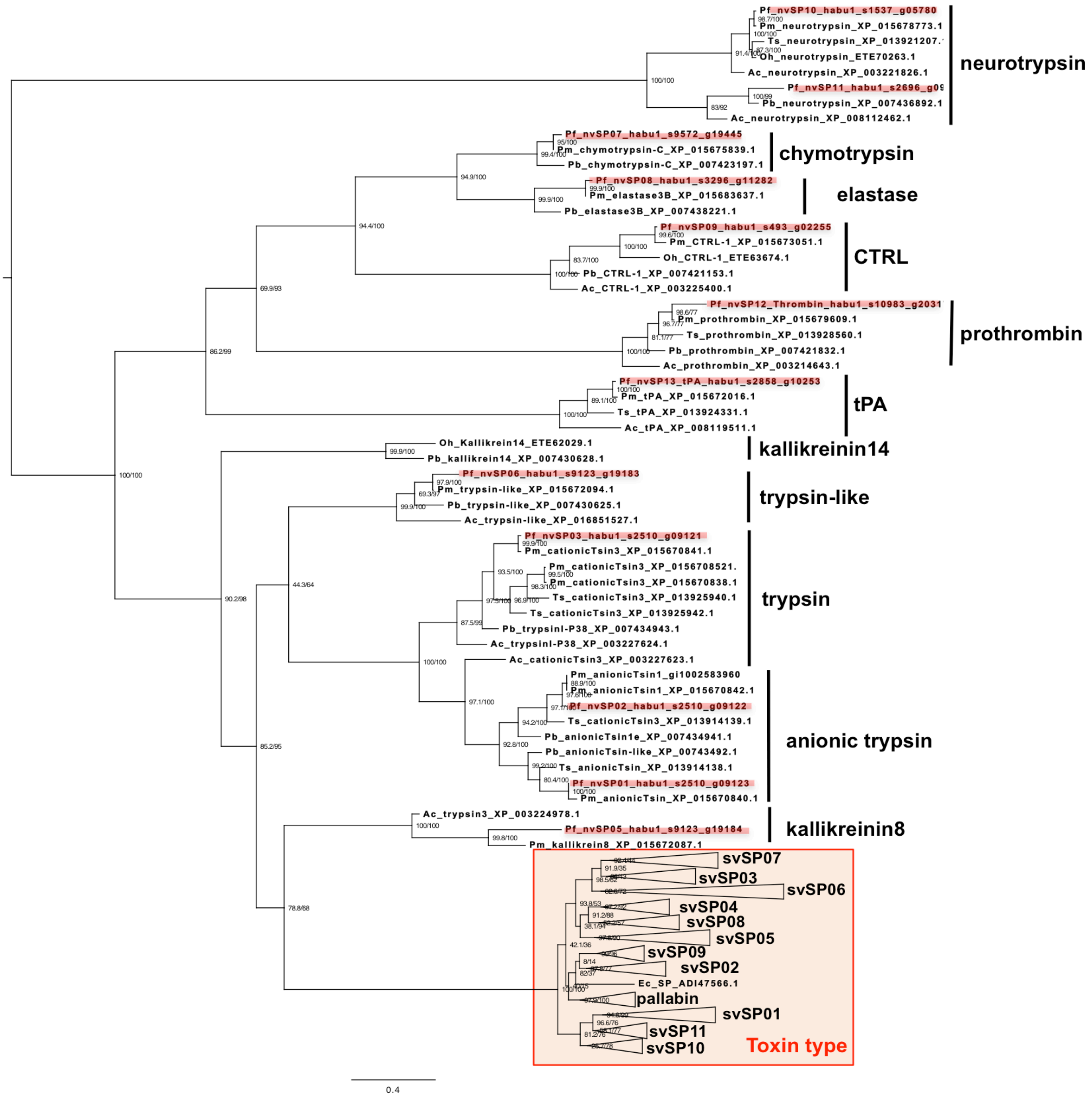

neurotrypsin
chymotrypsin
elastase
CTRL
prothrombin
tPA
kallikreinin14
trypsin-like
trypsin
anionic trypsin
kallikreinin8
Toxin type
svSP07
svSP03
svSP06
svSP04
svSP08
svSP05
svSP09
svSP02
Ec_SP_ADI47566.1
pallabin
svSP01
svSP11
svSP10
0.4

(d)

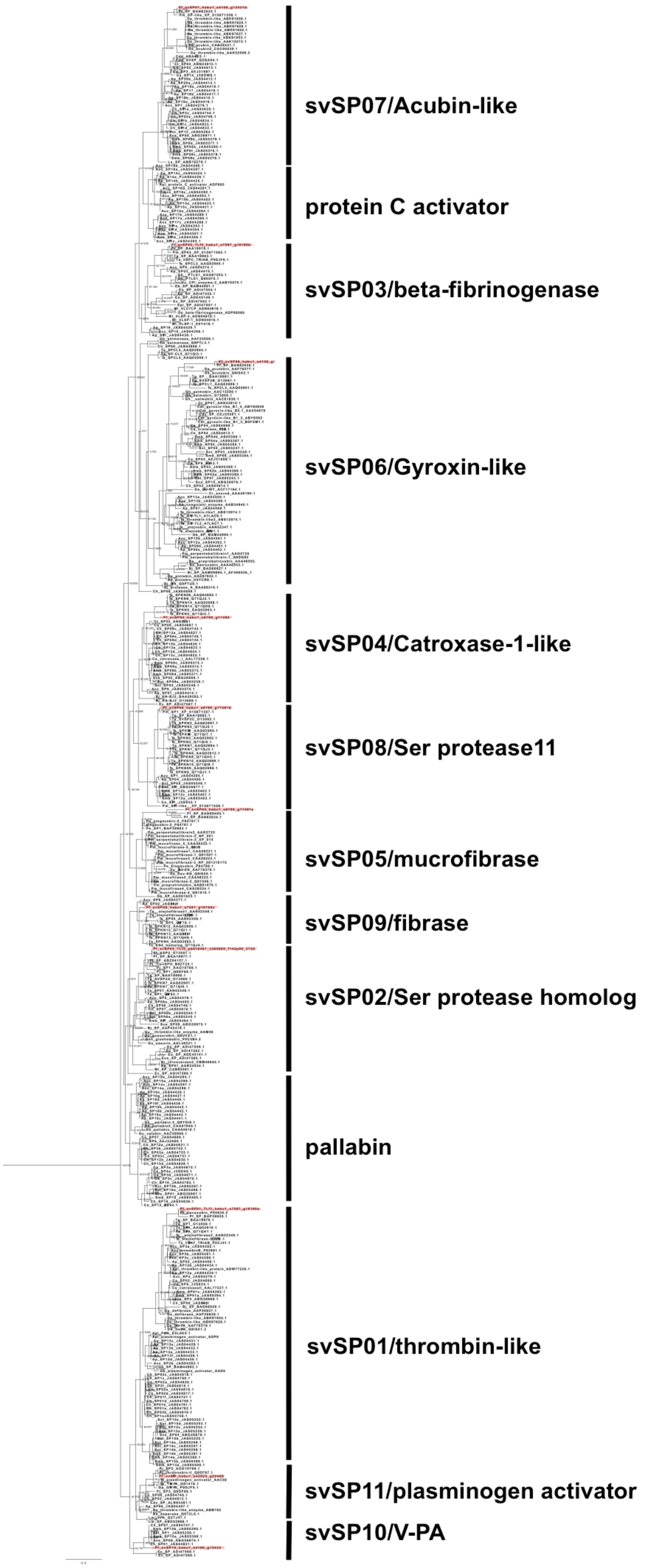
svSP07/Acubin-like
protein C activator
svSP03/beta-fibrinogenase
svSP06/Gyroxin-like
svSP04/Catroxase-1-like
svSP08/Ser protease11
svSP05/mucrofibrase
svSP09/fibrase
svSP02/Ser protease homolog
pallabin
svSP01/thrombin-like
svSP11/plasminogen activator
svSP10/V-PA

Supplementary Figure S5

(a) SV genes

Gene	Name	Locus	Gene Model ID	Transcripts
svCTLP01	IX/X-bp alpha	habu1_scaffold11033: 12909...19459: +		pb016481_c1025238_f1p0_769
svCTLP02	flavocetin-A alpha	habu1_scaffold375321: 193...344: +		pb016481_c1024339_f1p0_729
svCTLP03	stejaggregin-A-like alpha	habu1_scaffold81761: 5026...3746: -<br>habu1_scaffold10061: 161378...161319, 200154...203895: +	habu1_s10061_g19810c	pb016481_c1014958_f1p2_701
svCTLP04	rhodocetin/EmEMS16-like alpha homologue/ stejaggregin-B-like alpha	habu1_scaffold10061 : 161378...178891: -	habu1_s10061_g19810a	pb016481_c1017202_f1p0_682
svCTLP05	IX/X-bp beta_M	habu1_scaffold117853: 386...430: -<br>habu1_scaffold241669: 62...151: -<br>habu1_scaffold399881: 745...837: -<br>habu1_scaffold415837: 1...79: +<br>habu1_scaffold161573: 4...96: +		pb016480_c398342_f1p1_706
svCTLP06	flavocetin-A beta	habu1_scaffold10061 : 155982 ... 167335 : -	habu1_s10061_g19809	pb016481_c1018124_f1p2_702
svCTLP07	rhodocetinEMS16-like-beta	habu1_scaffold10061: 184382...193870: -	habu1_s10061_g19810b	pb016481_c1013095_f1p6_704
svCTLP08	C-lectin02	habu1_scaffold3168 : 129063 ... 132925 : -	habu1_s3168_g10977	habu1_s3168_g10977.t1
svCTLP09	Pf_CTLP_FIX/X BP-A_like	habu1_scaffold399941:1..7140:+<br>habu1_scaffold75126_1..926:+		pb016481_c1017356_f1p0_692
svCTLP10	pb016481_c1008289_f1p0_724	habu1_scaffold3168 : 97112 ... 103333 : +	habu1_s3168_g10975	pb016481_c1008289_f1p0_724

(b) NV genes

Gene	Name	Locus	Gene Model ID	Transcripts
nvCTLP01	C-type lectin	habu1_scaffold10061 : 25086 ... 32544 : +	habu1_s10061_g19804	habu1_s10061_g19804.t1
nvCTLP02	C-type lectin-like	habu1_scaffold10061 : 44806 ... 51663 : +	habu1_s10061_g19805	habu1_s10061_g19805.t1
nvCTLP03	C-type Lectin CRL-like	habu1_scaffold10061 : 52556 ... 89545 : +	habu1_s10061_g19806	habu1_s10061_g19806.t1
nvCTLP04	thymidine phosphorylase	habu1_scaffold10061 : 93712 ... 132005 : -	habu1_s10061_g19807	habu1_s10061_g19807.t1
nvCTLP05	C-type lectin：regenerating islet-derived protein 4-like	habu1_scaffold10061 : 139236 ... 146528 : +	habu1_s10061_g19808	habu1_s10061_g19808.t1
nvCTLP06	C-type lectin BiL-like	habu1_scaffold10589 : 1 ... 10687 : +	habu1_s10589_g20136	habu1_s10589_g20136.t1
nvCTLP07		habu1_scaffold10590 : 1 ... 60935 : -	habu1_s10590_g20137	habu1_s10590_g20137.t1
nvCTLP08		habu1_scaffold10590 : 62389 ... 78515 : -	habu1_s10590_g20138	habu1_s10590_g20138.t1
nvCTLP-09	C-type lectin BiL-like	habu1_scaffold20885 : 1 ... 6175 : -	habu1_s20885_g22359	habu1_s20885_g22359.t1
nvCTLP-10	C-type lectin Cal-like	habu1_scaffold20885 : 9716 ... 23508 : +	habu1_s20885_g22360	habu1_s20885_g22360.t1
nvCTLP-11		habu1_scaffold3162 : 1 ... 44155 : -	habu1_s3162_g10961	habu1_s3162_g10961.t1
nvCTLP-12	C-lectin01	habu1_scaffold3168 : 105257 ... 110525 : -	habu1_s3168_g10976	habu1_s3168_g10976.t1
nvCTLP-13	C-lectin03 (olfactory receptor 5V1-like [Python bivittatus)	habu1_scaffold3168 : 132988 ... 153875 : -	habu1_s3168_g10978	habu1_s3168_g10978.t1
nvCTLP-14	C-lectin04	habu1_scaffold3168 : 158476 ... 201437 : +	habu1_s3168_g10979	habu1_s3168_g10979.t1
nvCTLP-15	C-type lectin	habu1_scaffold5624 : 612654 ... 645778 : -	habu1_s5624_g16096	habu1_s5624_g16096.t1
nvCTLP-16	C-type lectin domain family 2 member A-like	habu1_scaffold2729 : 242646 ... 255780 : +	habu1_s2729_g09708	habu1_s2729_g09708.t1
nvCTLP-17	C-type lectin domain family 2 member B-like	habu1_scaffold4914 : 32203 ... 44645 : -	habu1_s4914_g14978	habu1_s4914_g14978.t1
nvCTLP-18	C-type lectin domain family 2 member D	habu1_scaffold16096 : 1593 ... 16985 : -	habu1_s16096_g21607	habu1_s16096_g21607.t1
nvCTLP-19	C-type lectin domain family 2 member D	habu1_scaffold16095 : 1 ... 33634 : +	habu1_s16095_g21604	habu1_s16095_g21604.t1
nvCTLP-20	C-type lectin domain family 2 member D-like isoform X1	habu1_scaffold7470 : 7148 ... 64124 : +	habu1_s7470_g18072	habu1_s7470_g18072.t1
nvCTLP-21	C-type lectin domain family 2 member D-like	habu1_scaffold2729 : 287626 ... 301914 : +	habu1_s2729_g09710	habu1_s2729_g09710.t1
nvCTLP-22	C-type lectin domain family 2 member D-like	habu1_scaffold2729 : 269596 ... 280053 : +	habu1_s2729_g09709	habu1_s2729_g09709.t1
nvCTLP-23	C-type lectin domain family 2 member F	habu1_scaffold650 : 139753 ... 147435 : -	habu1_s650_g02983	habu1_s650_g02983.t1
nvCTLP-24	C-type lectin domain family 2 member F	habu1_scaffold16095 : 20706 ... 30572 : +	habu1_s16095_g21605	habu1_s16095_g21605.t1
nvCTLP-25	C-type lectin domain family 4 member F	habu1_scaffold5437 : 1 ... 163815 : -	habu1_s5437_g15753	habu1_s5437_g15753.t1
nvCTLP-26	C-type lectin domain family 4 member F	habu1_scaffold5437 : 175001 ... 184325 : -	habu1_s5437_g15754	habu1_s5437_g15754.t1
nvCTLP-27	C-type lectin domain family 4 member G	habu1_scaffold9852 : 245036 ... 264242 : +	habu1_s9852_g19732	habu1_s9852_g19732.t1
nvCTLP-28	C-type lectin domain family 5 member A-like	habu1_scaffold356 : 6432 ... 15155 : -	habu1_s356_g01674	habu1_s356_g01674.t1
nvCTLP-29	C-type lectin domain family 10 member A	habu1_scaffold399929 : 2366 ... 26492 : +	habu1_s399929_g24822	habu1_s399929_g24822.t1
nvCTLP-30	C-type lectin domain family 10 member A	habu1_scaffold399929 : 28656 ... 34341 : +	habu1_s399929_g24823	habu1_s399929_g24823.t1
nvCTLP-31	C-type lectin domain family 10 member A	habu1_scaffold399929 : 35256 ... 46873 : +	habu1_s399929_g24824	habu1_s399929_g24824.t1
nvCTLP-32	C-type lectin domain family 11 member A	habu1_scaffold6124 : 381916 ... 408060 : +	habu1_s6124_g16816	habu1_s6124_g16816.t1
nvCTLP-33	C-type lectin domain family 17, member A: ow affinity immunoglobulin epsilon Fc receptor-like	habu1_scaffold9852 : 223468 ... 243595 : -	habu1_s9852_g19731	habu1_s9852_g19731.t1
nvCTLP-34	C-type lectin domain family 17, member A	habu1_scaffold2388 : 1078710 ... 1129431 : -	habu1_s2388_g08701	habu1_s2388_g08701.t1
nvCTLP-35	C-type lectin domain family 17, member A	habu1_scaffold4489 : 1161650 ... 1171504 : -	habu1_s4489_g14309	habu1_s4489_g14309.t1
nvCTLP-36	collectin-10	habu1_scaffold24 : 236156 ... 286149 : +	habu1_s24_g00208	habu1_s24_g00208.t1
nvCTLP-37	collectin-11	habu1_scaffold1294 : 282428 ... 290611 : -	habu1_s1294_g04540	habu1_s1294_g04540.t1
nvCTLP-38	collectin-12	habu1_scaffold2067 : 4679 ... 91925 : -	habu1_s2067_g07546	habu1_s2067_g07546.t1
nvCTLP-39	E-selectin isoform X1	habu1_scaffold20406 : 1 ... 19656 : -	habu1_s20406_g22285	habu1_s20406_g22285.t1
nvCTLP-40	olfactory receptor 5V1-like	habu1_scaffold3168 : 205916 ... 208854 : +	habu1_s3168_g10980	habu1_s3168_g10980.t1

Supplementary Figure S5 continued

(c)

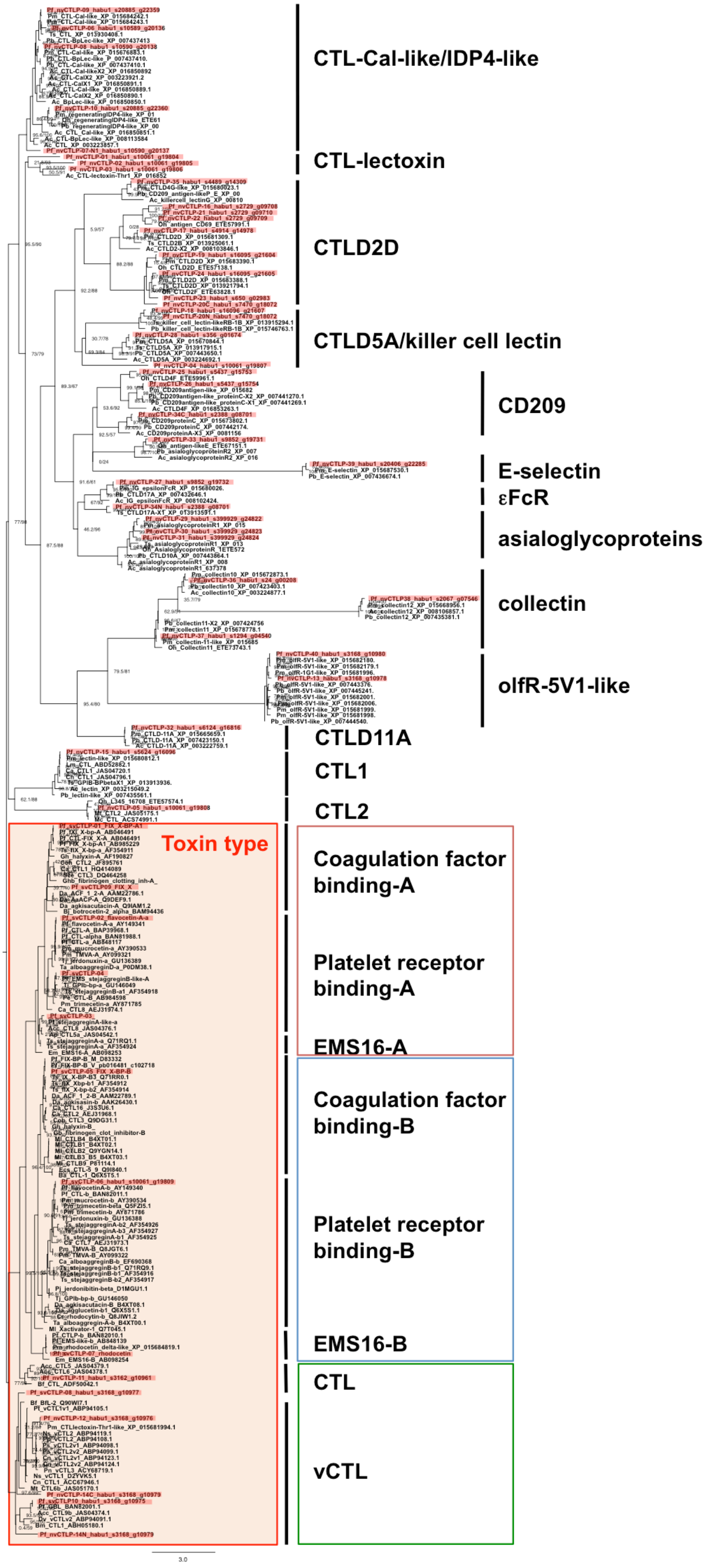

Supplementary Figure S6.

(a) SV genes

Gene	Name	Locus	Gene Model ID	Transcripts
svPLA2-01	Asp49PLA2/PL1a/PL2a	habu1_scaffold9571 : 7266 ... 11848 : +	habu1_s9571_g19434	pb016481_c1004773_f1p3_727
svPLA2-02	PL-Y/PL-X'/PL-B	habu1_scaffold47459 : 1268 ... 2998 : -	habu1_s47459_g23397a	pb016481_c1023909_f1p0_734
svPLA2-03	PLA-N	habu1_scaffold47459 : 1264 ... 3188 : -	habu1_s47459_g23397b	pb016481_c1016263_f1p3_727
svPLA2-04	BPI	habu1_scaffold50376:2642...1454: -		pb016481_c1030113_f1p0_731
svPLA2-05	BPII	habu1_scaffold9571 : 1 ... 1869 : -		pb016481_c1028655_f1p0_728
svPLA2-06	BPIII	habu1_scaffold401404: 1...365: -		
svPLA2-07	PL1b/PL2b	habu1_scaffold9570:1...742: +	habu1_s9570_g19432	pb016481_c1024179_f1p5_745
svPLA2-08	PfPLA6	habu1_scaffold9571 : 1 ... 8417 : -	habu1_s9571_g19433	habu1_s9571_g19433.t2
svPLA2-09	pgPLA2b	habu1_scaffold9571:16216...17662: +		pb016481_c1027830_f1p0_729

(b) NV genes

Gene	Name	Locus	Gene Model ID	Transcripts
nvPLA2-01	PLA2_G1_1	habu1_scaffold9792:202026..204267: +	habu1_s9792_g19655	habu1_s9792_g19655.t1
nvPLA2-02	PLA2_G1_2	habu1_scaffold9792:206686..208528: +	habu1_s9792_g19656	habu1_s9792_g19656.t1
nvPLA2-03	PLA2_G2E_1	habu1_scaffold4562 : 336326 ... 340919: -	habu1_s4562_g14470	habu1_s4562_g14470.t1
nvPLA2-04	PLA2_G3_2	habu1_scaffold2696 : 770786 ... 776844: +	habu1_s2696_g09640	habu1_s2696_g09640.t1
nvPLA2-05	PLA2_G3_4	habu1_scaffold3088 : 458 ... 5355: -	habu1_s3088_g10883	habu1_s3088_g10883.t1
nvPLA2-06	PLA2_G10_1	habu1_scaffold194 : 345256 ... 364398: +	habu1_s194_g01103	habu1_s194_g01103.t1
nvPLA2-07	PLA2_G10_2	habu1_scaffold194 : 366490 ... 382195: -	habu1_s194_g01104	habu1_s194_g01104.t1
nvPLA2-08	PLA2_G10_3	habu1_scaffold2789 : 707889 ... 711217: +	habu1_s2789_g09989	habu1_s2789_g09989.t1
nvPLA2-09	PLA2_G12A_1	habu1_scaffold3769 : 1122983 ... 1127025: +	habu1_s3769_g12642	habu1_s3769_g12642.t1
nvPLA2-10	PLA2_G12B_1	habu1_scaffold2074 : 358299 ... 399065: -	habu1_s2074_g07581	habu1_s2074_g07581.t1
nvPLA2-11	PLA2_G15_1	habu1_scaffold499 : 736999 ... 788724: +	habu1_s499_g02314	habu1_s499_g02314.t1
nvPLA2-12	PLA2_G15_3	habu1_scaffold53923 : 12382 ... 17295: -	habu1_s53923_g23499	habu1_s53923_g23499.t1
nvPLA2-13	PLA2_G15_4	habu1_scaffold5562 : 106910 ... 108039: -		pb016480_c10250_f1p0_1130
nvPLA2-14	PLA2	habu1_scaffold2192 : 72907 ... 74929: -	habu1_s2192_g07871	habu1_s2192_g07871.t1
nvPLA2-15	PLA2G4A	habu1_scaffold8317 : 5635 ... 95005: -	habu1_s8317_g18615	habu1_s8317_g18615.t1
nvPLA2-16	PLA2G4B_1	habu1_scaffold3646:1..6155: -	habu1_s3646_g12259	habu1_s3646_g12259.t1
nvPLA2-17	PLA2G4B_2	habu1_scaffold3645 : 202517 ... 283015: -	habu1_s3645_g12258	habu1_s3645_g12258.t1
nvPLA2-18	PLA2G4C_3	habu1_scaffold11920:77736..127822: +	habu1_s11920_g20651	habu1_s11920_g20651.t1
nvPLA2-19	PLA2G4C_4	habu1_scaffold32464 : 1 ... 60356: +	habu1_s32464_g23033	habu1_s32464_g23033.t1
nvPLA2-20	PLA2_B	habu1_scaffold2320 : 573149 ... 601069 : +	habu1_s2320_g08440	habu1_s2320_g08440.t1
nvPLA2-21	PLA2_B	habu1_scaffold2321 : 2106 ... 33861 : +	habu1_s2321_g08441	habu1_s2321_g08441.t1
nvPLA2-22	PLA2G4F	habu1_scaffold2323 : 1 ... 38699 : +	habu1_s2323_g08442	habu1_s2323_g08442.t1
nvPLA2-23	PLA2G4E_2	habu1_scaffold3645 : 9056 ... 58091: +	habu1_s3645_g12255	habu1_s3645_g12255.t1
nvPLA2-24	PLA2G4E_4	habu1_scaffold2324 : 1 ... 70129: +	habu1_s2324_g08443	habu1_s2324_g08443.t1
nvPLA2-25	PLA2G4E_5	habu1_scaffold2324 : 74886 ... 119324: +	habu1_s2324_g08444	habu1_s2324_g08444.t1
nvPLA2-26	PLA2G4E_6	habu1_scaffold2324 : 122456 ... 211367: +	habu1_s2324_g08445	habu1_s2324_g08445.t1
nvPLA2-27	PLA2G6	habu1_scaffold7565 : 780358 ... 836117: +	habu1_s7565_g18177	habu1_s7565_g18177.t1
nvPLA2-28	PNPLA8	habu1_scaffold5091 : 1 ... 61165: -	habu1_s5091_g15231	habu1_s5091_g15231.t1
nvPLA2-29	PLA1	habu1_scaffold5830 : 1665 ... 118015 : -	habu1_s5830_g16316	habu1_s5830_g16316.t1
nvPLA2-30	PLA2_G16	habu1_scaffold1542 : 1 ... 34425 : +	habu1_s1542_g05787	habu1_s1542_g05787.t1
nvPLA2-31	PLA1mA	habu1_scaffold19666 : 1 ... 11400 : +	habu1_s19666_g22161	habu1_s19666_g22161.t1

Supplementary Figure S6 continued

(c)

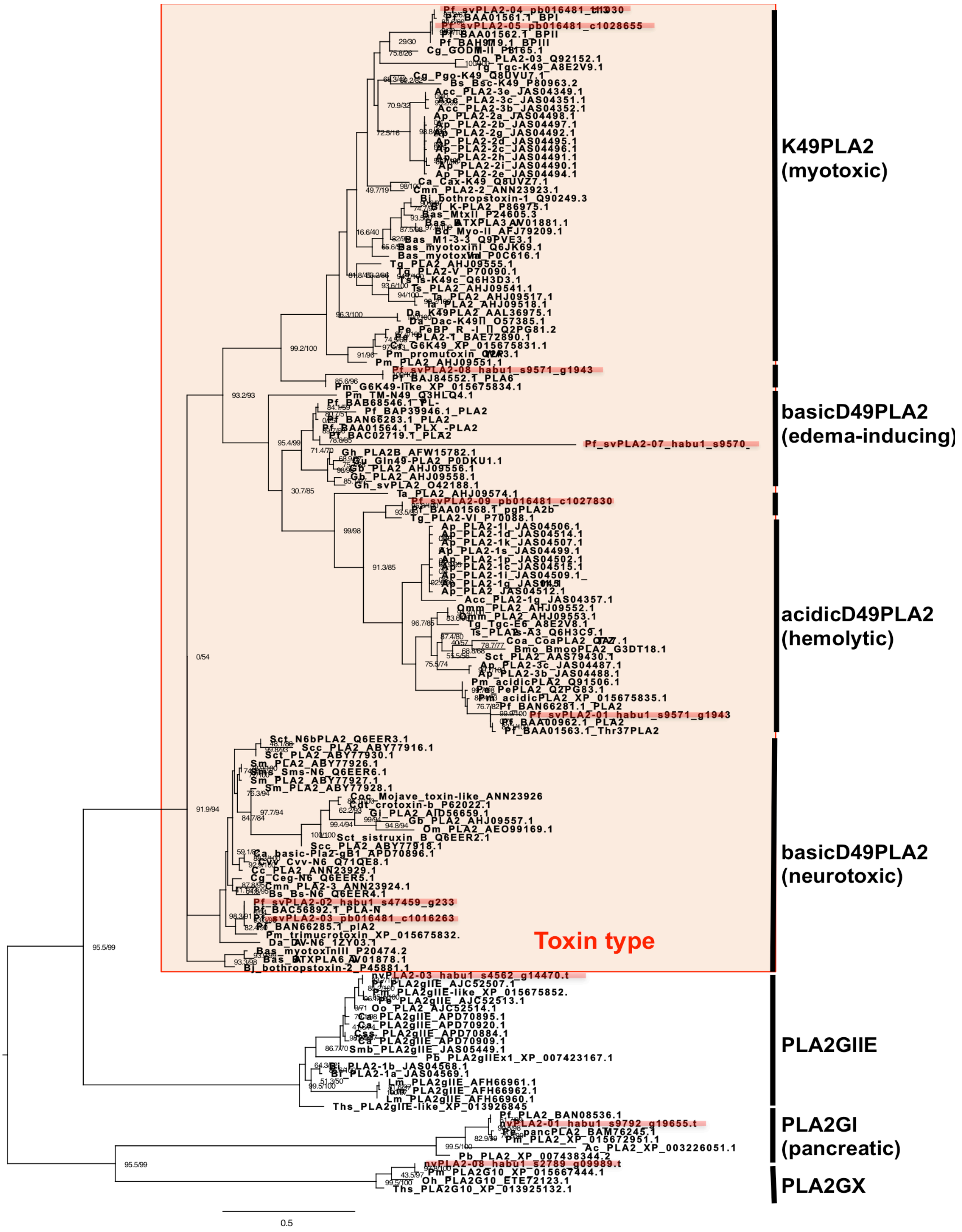

Supplementary Figure S7

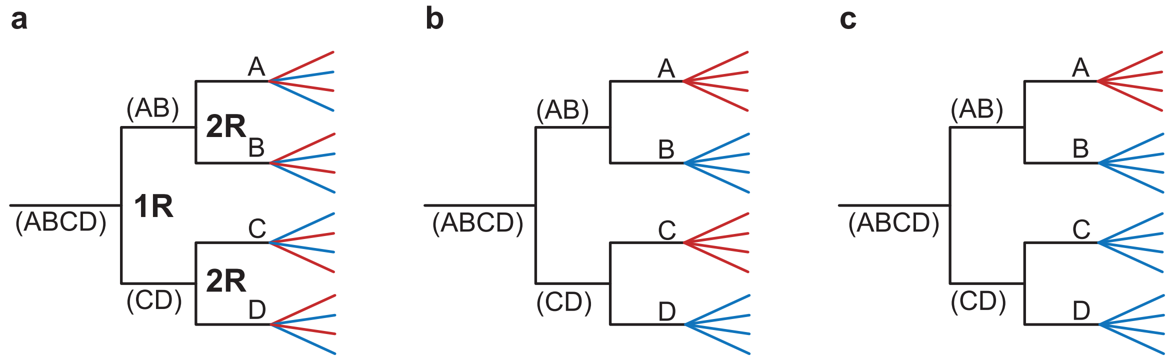

Supplementary Figure S8.

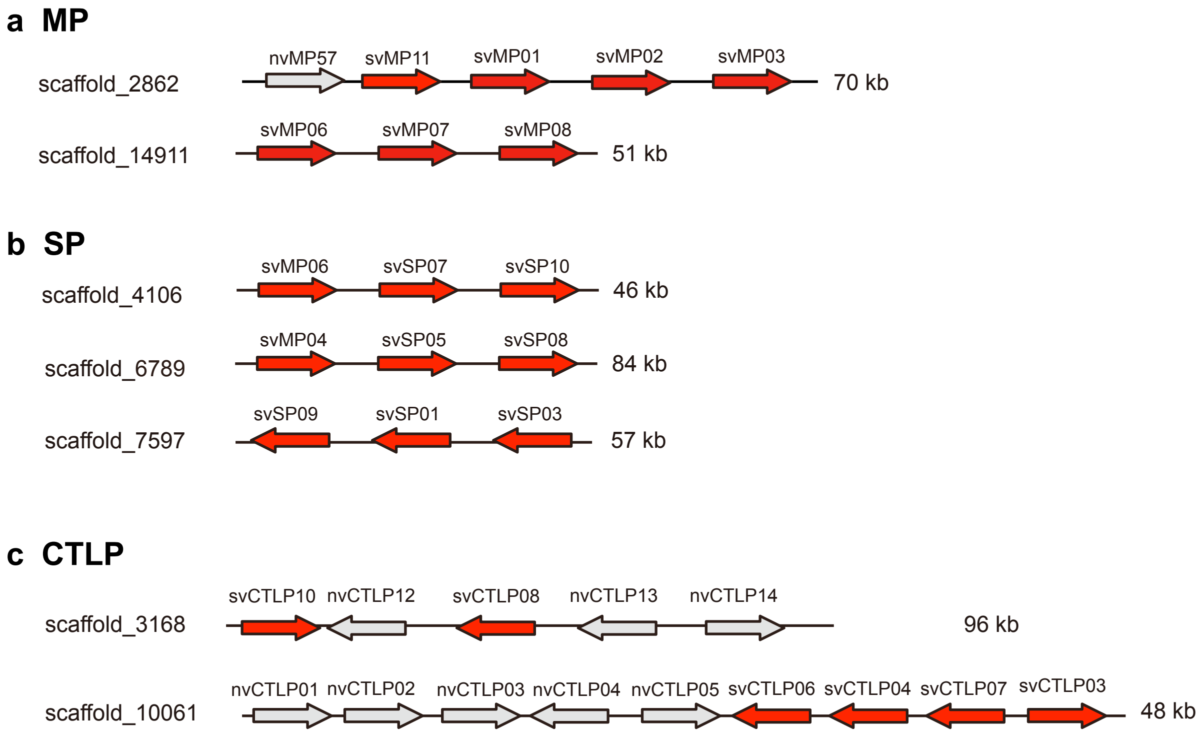

Supplemental Figure S9

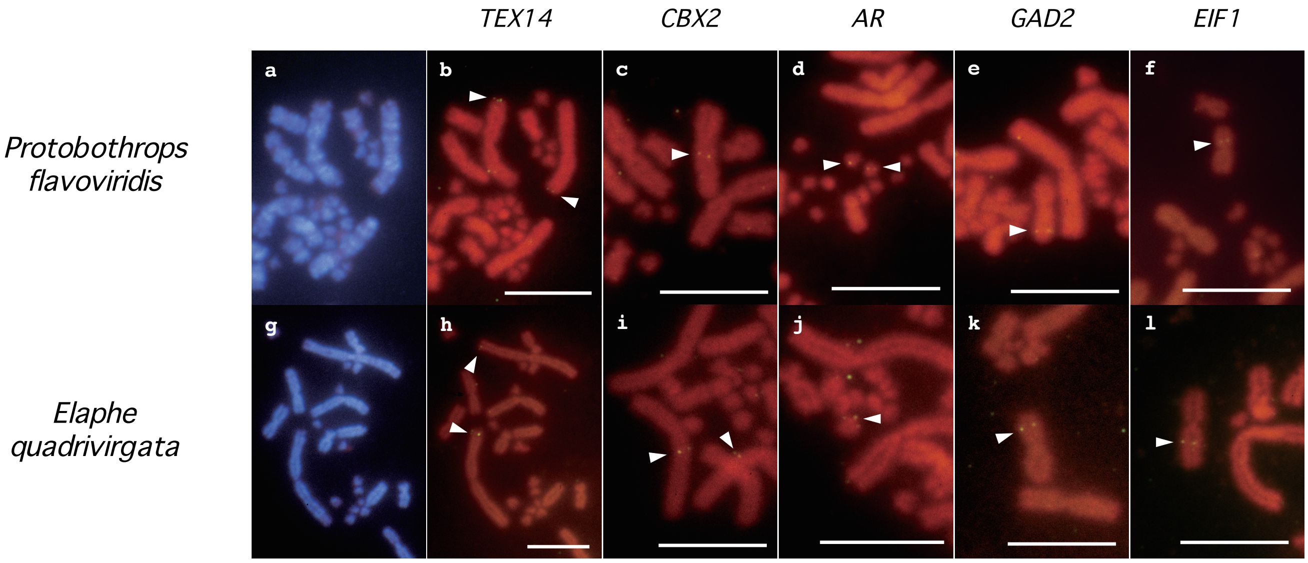

Supprementary Figure S10

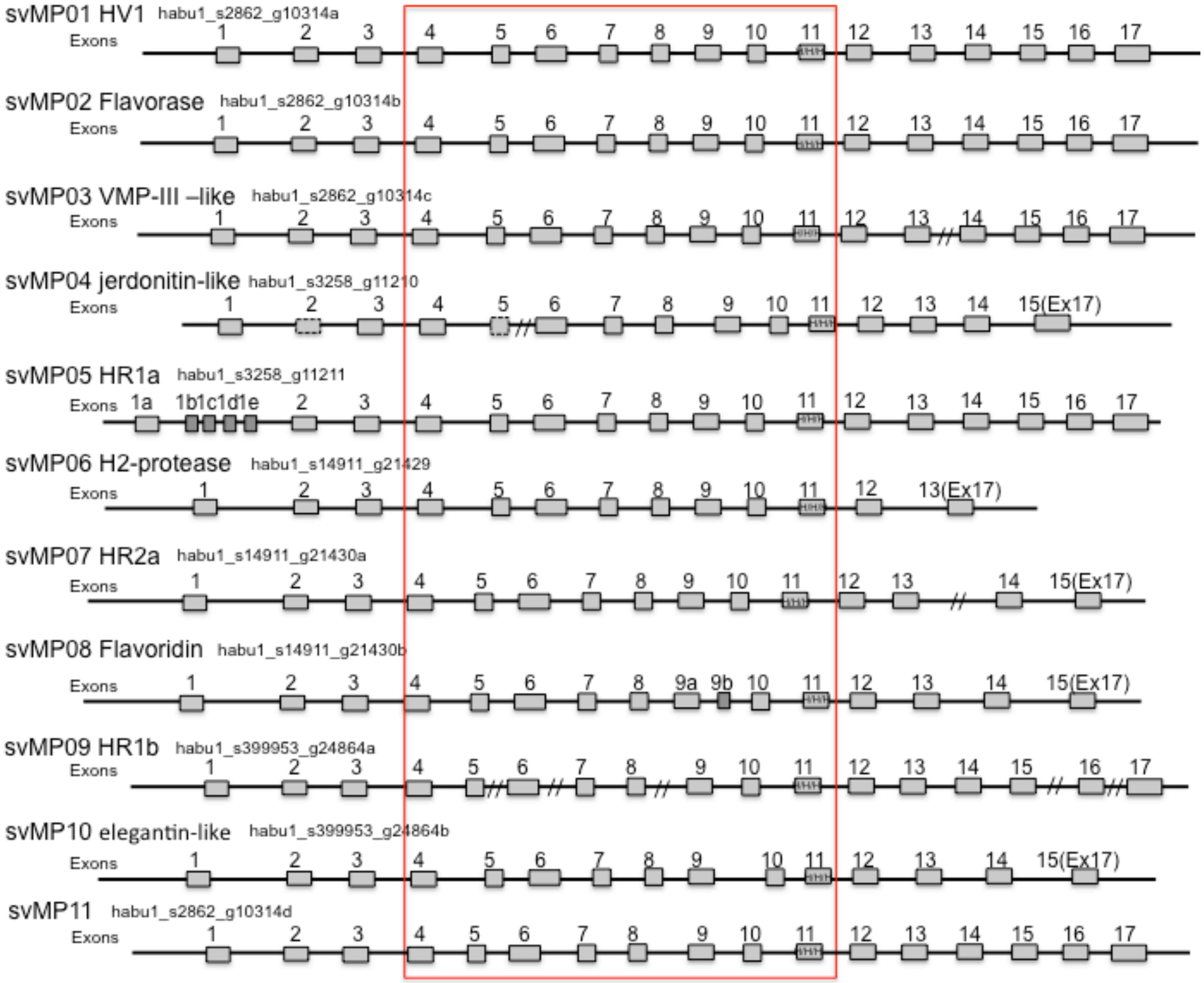

Supprementary Figure S11

a

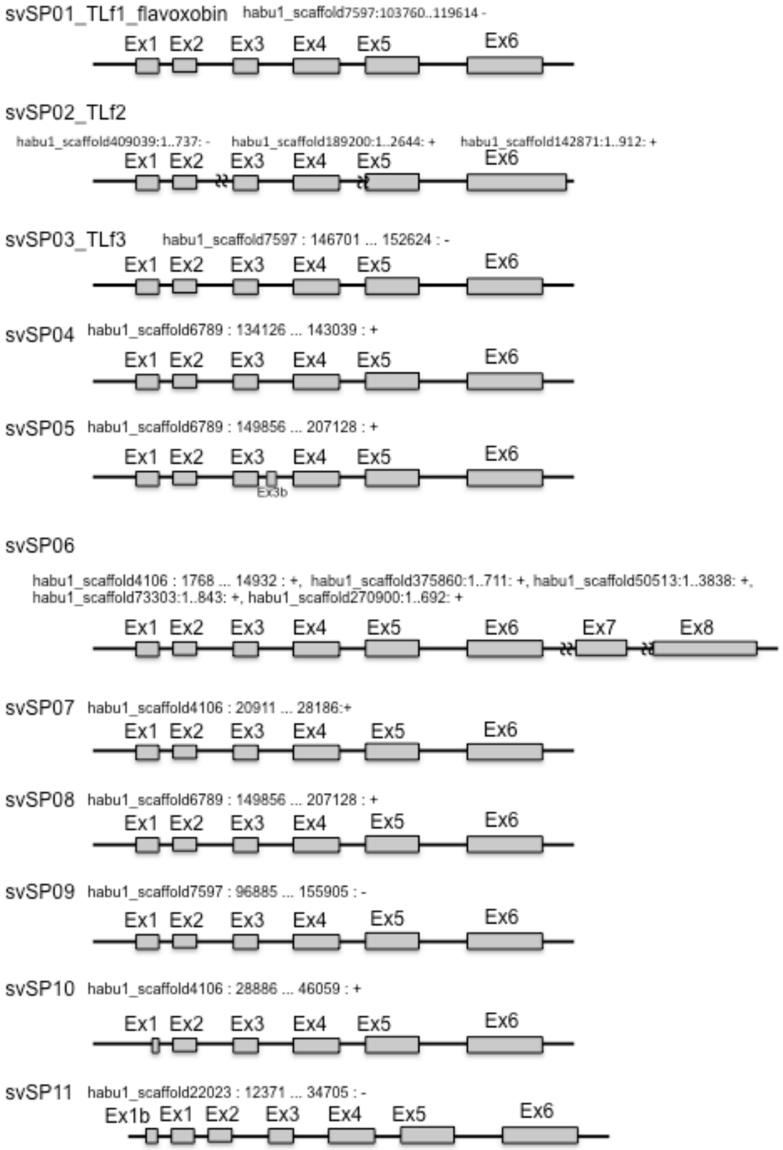

b

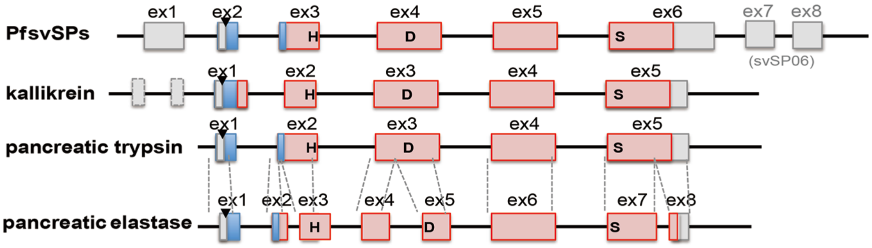

Supplementary Figure S12

(a)

Gene	Name	Locus	Gene Model ID	Transcripts
3FTX01 (sv3FTX)	Pf_3FTX-01	habu1_scaffold4579 : 59513 ... 76175 : -	habu1_s4579_g14476	pb016480_c396810_f1p0_507
3FTX02 (sv3FTX)	Pf_3FTX-02	habu1_scaffold4579 : 119426 ... 122076 : +	habu1_s4579_g14477	habu1_s4579_g14477.t1
3FTX03 (sv3FTX)	Pf_3FTX-03	habu1_scaffold4579 : 142940 ... 144965 : -	habu1_s4579_g14478	habu1_s4579_g14478.t1
3FTX04 (sv3FTX)	Pf_3FTX-04	habu1_scaffold4579 : 45965 ... 48865 : -	habu1_s4579_g14475	habu1_s4579_g14475.t1
3FTX05 (nv3FTX)	Pf_3FTX-05 (UPAR_LY6, CD59A)	habu1_scaffold138 : 73600 ... 130505 : -	habu1_s138_g00722	habu1_s138_g00722.t1
3FTX06 (nv3FTX)	Pf_3FTX-06 (CD59B)	habu1_scaffold138 : 62681 ... 68945 : -	habu1_s138_g00721	habu1_s138_g00721.t1

(b)

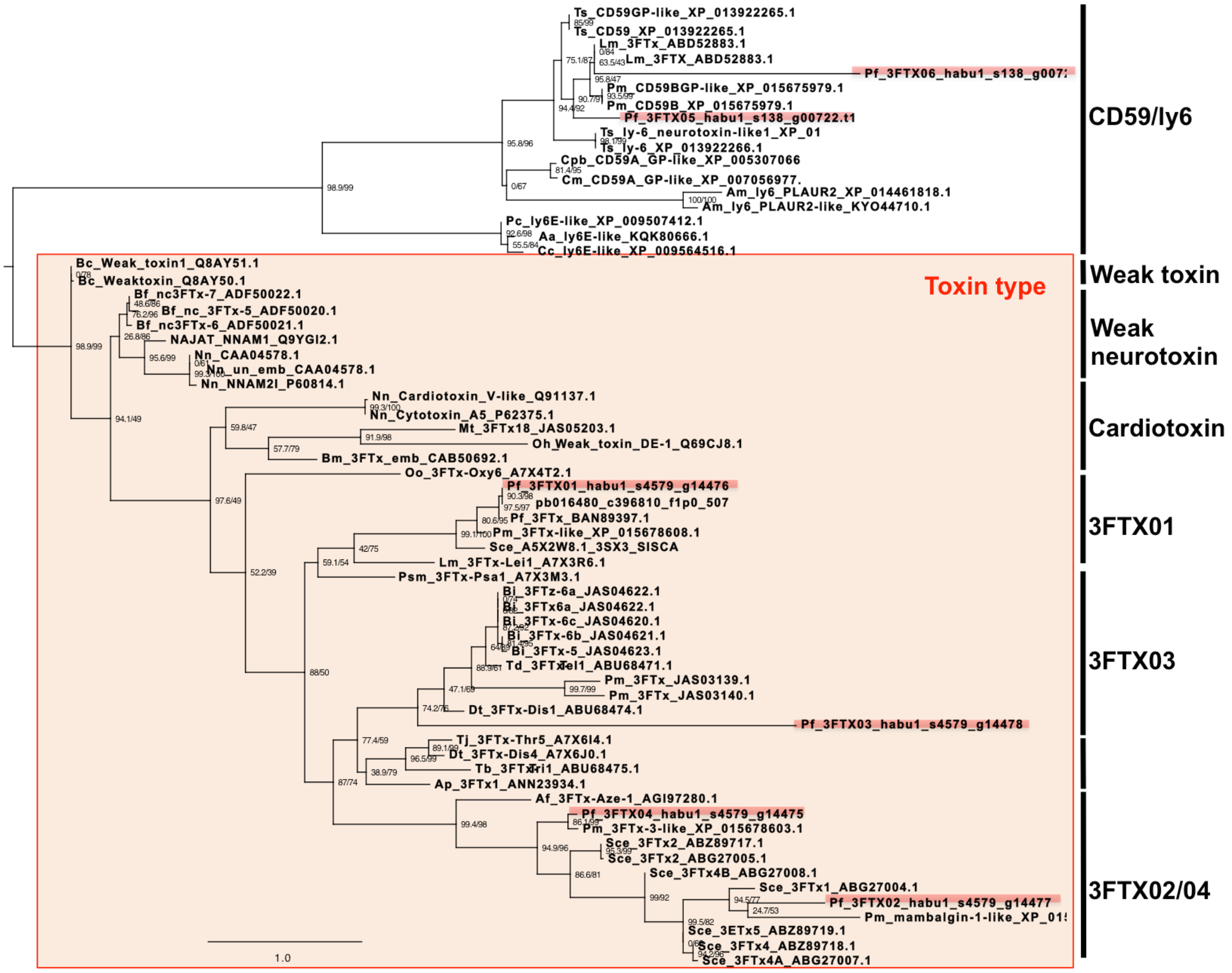

Supplementary Figure S 13

(a)

Gene		Name	Locus	Gene model ID	Transcripts
APase01	APaseN (svAPase)	aminopeptidase N	habu1_scaffold1390 : 1416444 ... 1437202 : +	habu1_s1390_g05001	pb016481_c405527_f2p3_3459
APase02	APaseNPEPL	aminopeptidase NPEPL	habu1_scaffold5205 : 360816 ... 379669 : +	habu1_s5205_g15384	habu1_s5205_g15384.t1
APase03	APaseA (svAPase)	aminopeptidase A	habu1_scaffold3769 : 814477 ... 861472 : -	habu1_s3769_g12636	pb016481_c564840_f1p8_3942
APase04	APaseB	aminopeptidase B	habu1_scaffold8948 : 297629 ... 311470 : +	habu1_s8948_g19021	habu1_s8948_g19021.t1
APase05	APaseO	aminopeptidase O	habu1_scaffold4166 : 161346 ... 345243 : +	habu1_s4166_g13605	habu1_s4166_g13605.t1
APase06	APaseQ	aminopeptidase Q	habu1_scaffold3446 : 147415 ... 149419 : -	habu1_s3446_g11762	habu1_s3446_g11762.t1
APase07	APaseD	aspartyl aminopeptidase	habu1_scaffold133 : 520486 ... 540509 : +	habu1_s133_g00676	habu1_s133_g00676.t1
APase08	APaseM1	methionine aminopeptidase 1	habu1_scaffold7599 : 75984 ... 96985 : -	habu1_s7599_g18195	habu1_s7599_g18195.t1
APase09	APaseM2	methionine aminopeptidase 2	habu1_scaffold9114 : 242443 ... 254705 : -	habu1_s9114_g19166	habu1_s9114_g19166.t1
APase10	XP APase1	xaa-pro aminopeptidase 1	habu1_scaffold4753 : 86146 ... 168417 : +	habu1_s4753_g14695	habu1_s4753_g14695.t1
APase11	XP APase2	xaa-pro aminopeptidase 2	habu1_scaffold4159 : 322646 ... 384475 : +	habu1_s4159_g13556	habu1_s4159_g13556.t1
APase12	XP APase3	xaa-pro aminopeptidase 3	habu1_scaffold1233 : 167726 ... 197694 : +	habu1_s1233_g04248	habu1_s1233_g04248.t1

(b)

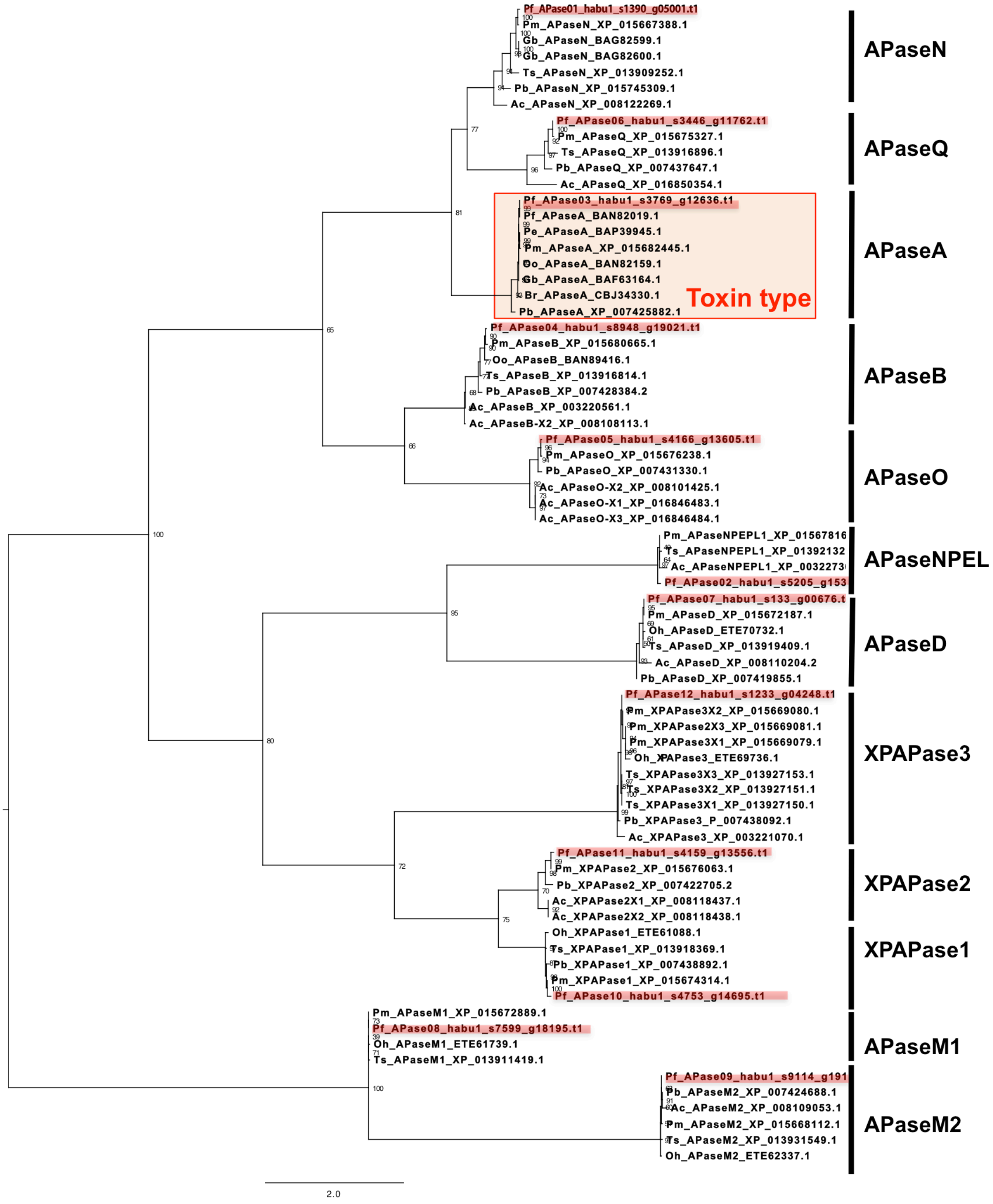

Supplementary Figure S 14

(a)

Gene	Name	Locus	Gene model ID	Transcripts
CRISP01 (svCRISP)	CRISP/triflin	habu1_scaffold22025: 1...14800: -	habu1_s22025_g22470a	pb016480_c42550_f61p14_1314
CRISP02 (svCRISP)	CRISP_like	habu1_scaffold22025:30009...67537: +	habu1_s22025_g22470b	habu1_s22025_g22470.t1
CRISP03 (nvCRISP)	CRISP w egf-like domain	habu1_scaffold1243 : 310296 ... 329704 : +	habu1_s1243_g04322	habu1_s1243_g04322.t1
CRISP04 (nvCRISP)	CRISP w egf-like domain	habu1_scaffold264 : 3584 ... 27775 : -	habu1_s264_g01336	habu1_s264_g01336.t1

(b)

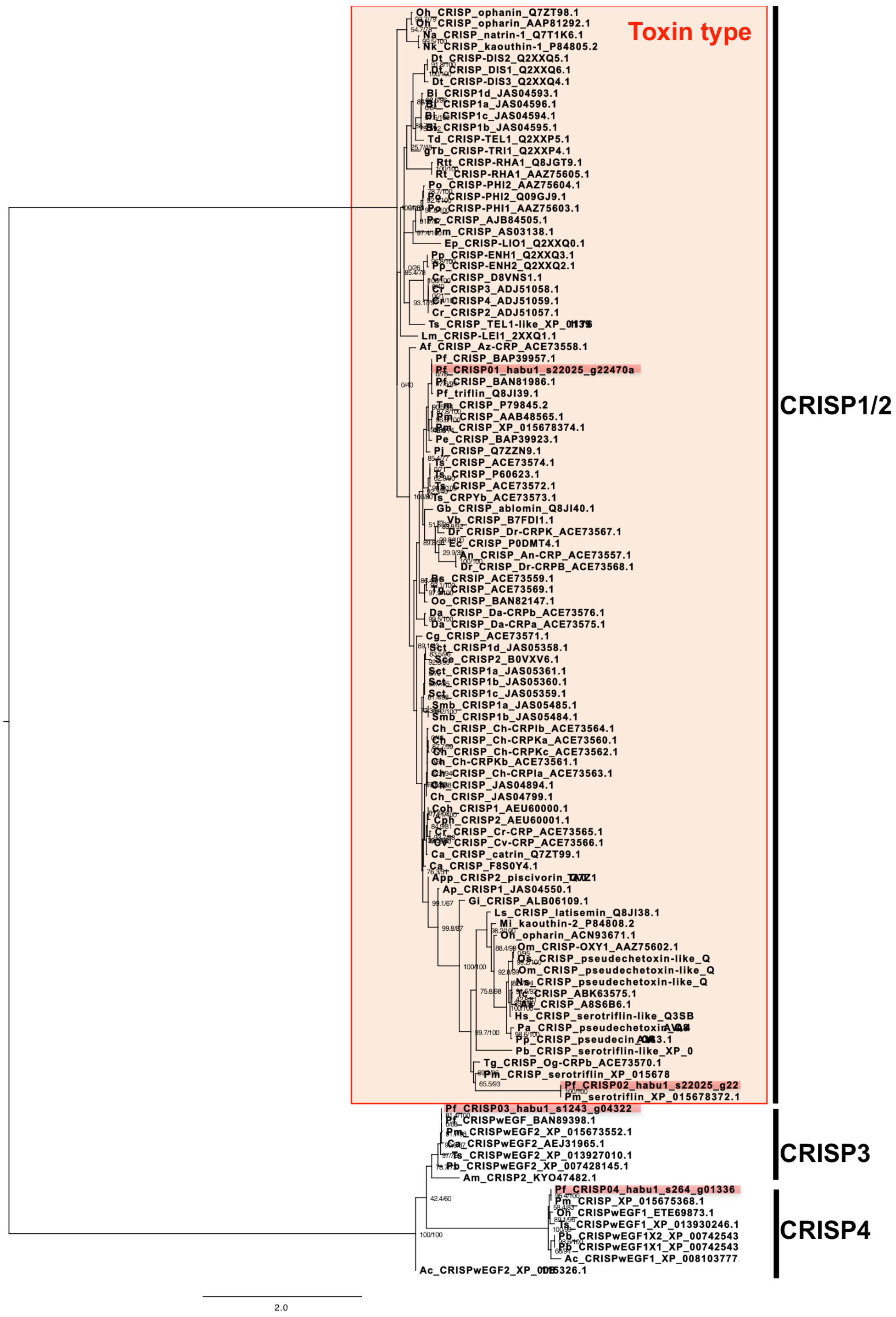

(c)

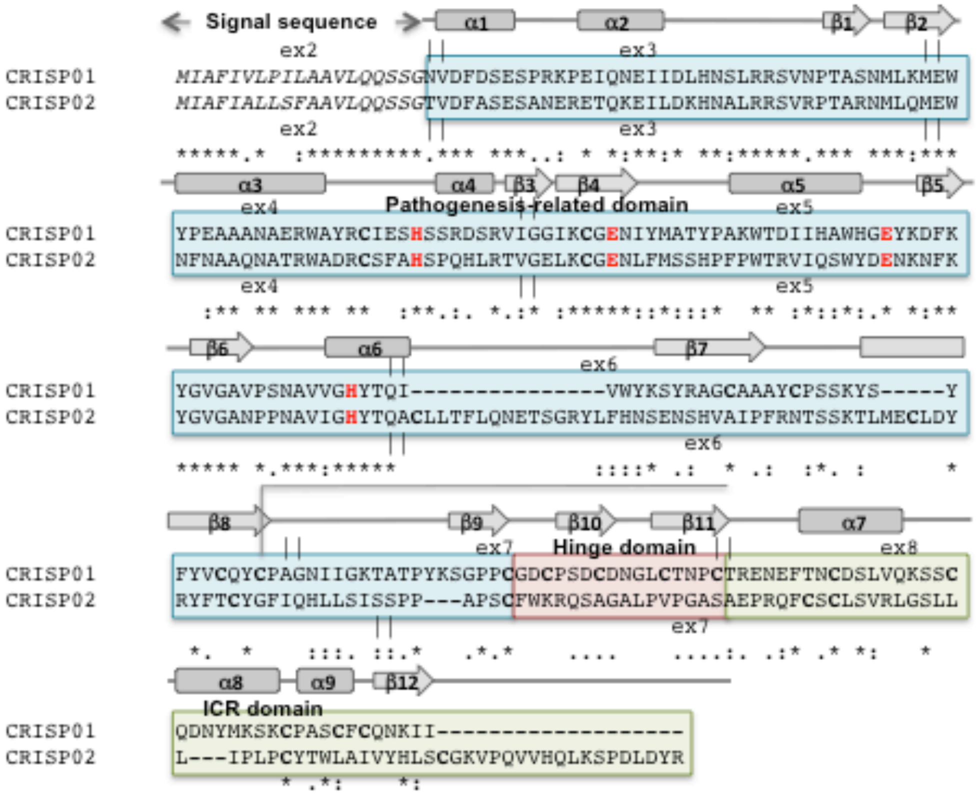

(d)

```
                                                                    ex1          ||
CRISP03     MKAGRAAWRWLEA----FAFLVLLAVSLPPASPALGSSEERLRQACNTCRGIADRFTQGLTDTAKKNFGG
CRISP04     MAFSR-----MKVPPALFSFLLLFQ-----WMPNSGSSQQ---DTCQTCRGLVDNFNKGLERTQRENFGG
                                                                    ex2          ||
            *  .*     ::.    *:**:*:         *  ***::   ::*:****:.*.*.:**  * ::****

                                                          DUF34556(TLR4 regulator)
                ex2        ||              ex3                        ||        ex4
CRISP03     GNTAWEEKTLSKYESSEIRLVEIIENLCDSSNFECNNMVEEHEEHIENWWFKWKKKYPDLFKWLCIETIE
CRISP04     GNTAWEEEKLAKYANSETRLLEVLESVCSTSDFACHQLLERSEDHVEHWWFHEQQQHPDFFQWLCMDTLK
                ex3        ||              ex4                        ||        ex5
            *******:.*:** .** **:*::*.:*.:*:* *::::*. *:*:*:***: ::::**:*:***::*::

                        ||                                   ex5
CRISP03     VCCPAGTHGPDCVACRGGSERPCHGNGNCDGDGTRAGDGSCKCQKEYQGEFCLDCSDGYYNFYKNDTHSV
CRISP04     LCCPSGTYGPDCQTCPGGAEKPCSGYGQCDGEGTSGGTGLCMCQTGYGGPFCSECGDGYYEAARNDSHLV
                        ||                                   ex6
            :***:**:**** :* **:*:** * *:***:** .* * * **. * * ** :*.****:  :**:* *

            Cys-rich (Furin-like) repeat
              ||              ex6                  ||              ex7                ||
CRISP03     CTACHDSCKTCTGATNKDCKDCKEGWLRNEEACVDEDECAVEESPCNSDQYCLNTDGSFSCKACDLSCLG
CRISP04     CAECYRACGRCSGPEDTSCLRCKRGWMLHNQRCIDIDECGTDMAHCRSNQFCVNTEGSYECRDCAKPCIG
              ||              ex7                  ||              ex8                ||
            *: *: :*  *:*. :..*  **.**: ::: *:* ***..: : *.*:*:*:**:**:.*: *  .*:*

            Cys-rich (Furin-like) repeat                  Ca_binding EGF_like domain
                     ex8                 ||                    ex9
CRISP03     CTGEGPNKCKSCVTGYEMKEETCTDVDECS--QTEEVCTRENTNCINTPGGYKCICSEGFEDKDDICVPS
CRISP04     CMGAGPSRCKKCNKGYQRDGVKCLDVDECAGEVEEPVCTGANEVCENTDGSYRCVCAEGHLRKEGICV-E
                     ex9                 ||                    ex10
            * * **.:**.* .**: .  .* *****:    * ***  *  * ** *.*:*:*:**.  *:.***

               ||          ex10
CRISP03     IKAEEKTSANISSPDTHEDL--------------------------------------------------
CRISP04     DKPPDAPEKGFFDDITDDEVVVLQQMFFGAIICALATLAAKGDMVFTAIFIGAVAAMAGYWMSERSDRVL
               ||          ex11
              *:*

CRISP03     -------
CRISP04     DGFMKGR
```

Supplementary Figure S15.

(a)

Gene	Name	Locus	Gene model ID	Transcripts
Ves01 (svVesp)	Vespryn	habu1_scaffold402940 : 157656 ... 165646 : +	habu1_s402940_g24938	habu1_s402940_g24938.t1
Ves02	butyrophilin subfamily	habu1_scaffold402940 : 136268 ... 173215 : -	habu1_s402940_g24937	habu1_s402940_g24937.t1
Ves03	spry domain-containing socs box protein 3 isoform x1	habu1_scaffold2038 : 674725 ... 686992 : +	habu1_s2038_g07466	habu1_s2038_g07466.t1
Ves04	spry domain-containing protein	habu1_scaffold1489 : 27447 ... 30825 : -	habu1_s1489_g05514	habu1_s1489_g05514.t1
Ves05	spry domain-containing socs box protein 4 isoform x1	habu1_scaffold2998 : 118432 ... 298625 : -	habu1_s2998_g10640	habu1_s2998_g10640.t1
Ves06	F-box/SPRY domain-containing protein 1	habu1_scaffold12804 : 318189 ... 339575 : -	habu1_s12804_g20892	habu1_s12804_g20892.t1
Ves07	SPRY domain-containing protein 7	habu1_scaffold13689 : 124686 ... 130582 : +	habu1_s13689_g21202	habu1_s13689_g21202.t1
Ves08	SPRY domain-containing protein 3	habu1_scaffold1516 : 380747 ... 424339 : +	habu1_s1516_g05661	habu1_s1516_g05661.t1
Ves09	fibronectin type III and SPRY domain-containing protein 2-like	habu1_scaffold3496 : 592235 ... 607031 : +	habu1_s3496_g11883	habu1_s3496_g11883.t1
Ves10	spry domain-containing socs box protein 1	habu1_scaffold3776 : 8796 ... 59035 : +	habu1_s3776_g12675	habu1_s3776_g12675.t1
Ves11	fibronectin type iii and spry domain-containing protein 1	habu1_scaffold2525 : 6768 ... 32830 : -	habu1_s2525_g09169	habu1_s2525_g09169.t1
Ves12	ring finger and spry domain-containing protein 1	habu1_scaffold503 : 116026 ... 143018 : +	habu1_s503_g02344	habu1_s503_g02344.t1

(b)

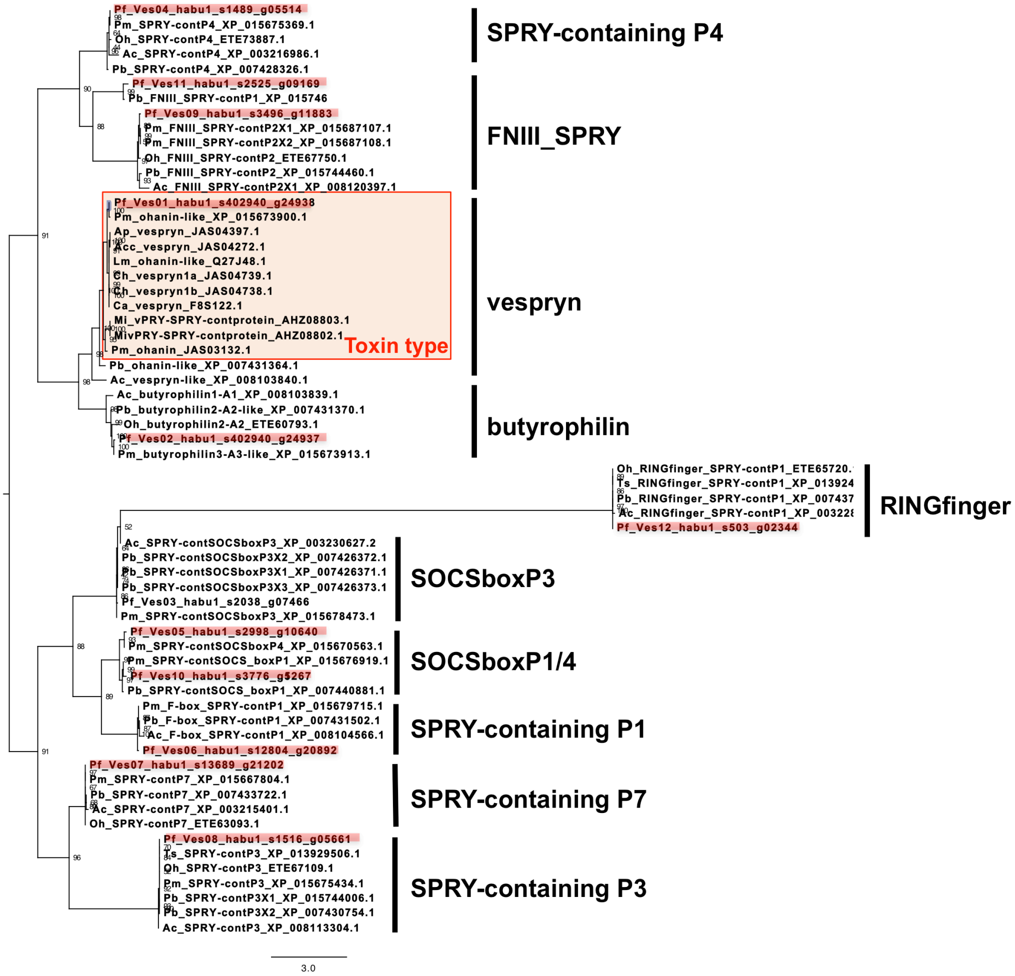

Supplementary Figure S16

(a)

Gene	Name	Locus	Gene Model ID	Transcripts
5Nase01 (sv5Nase)	sv5Nase	habu1_scaffold6028 : 169856 ... 203427 : +	habu1_s6028_g16570	pb016481_c551957_f58p63_2527
5Nase02 (nv5Nase)	5 -nucleotidase domain-containing protein 2-like	habu1_scaffold1426 : 2901530 ... 2936315 : +	habu1_s1426_g05181	habu1_s1426_g05181.t1
5Nase03 (nv5Nase)	5 -nucleotidase domain-containing protein 2	habu1_scaffold6846 : 55556 ... 83885 : +	habu1_s6846_g17547	habu1_s6846_g17547.t1
5Nase04 (nv5Nase)	5 -nucleotidase domain-containing protein 3	habu1_scaffold428 : 301964 ... 331735 : -	habu1_s428_g02066	habu1_s428_g02066.t1
5Nase05 (nv5Nase)	cytosolic purine 5 -nucleotidase isoform	habu1_scaffold1344 : 510687 ... 545803 : -	habu1_s1344_g04804	habu1_s1344_g04804.t1
5Nase06 (nv5Nase)	5_nucleotid	habu1_scaffold1548 : 105056 ... 126204 : +	habu1_s1548_g05833	habu1_s1548_g05833.t1
5Nase07 (nv5Nase)	5_nucleotid	habu1_scaffold8872 : 26926 ... 110043 : +	habu1_s8872_g18961	habu1_s8872_g18961.t1
5Nase08 (nv5Nase)	5 (3)- cytosolic type	habu1_scaffold3440 : 270746 ... 276672 : +	habu1_s3440_g11638	habu1_s3440_g11638.t1
5Nase09 (nv5Nase)	3 (2) -bisphosphate nucleotidase	habu1_scaffold304 : 182426 ... 196985 : -	habu1_s304_g01433	habu1_s304_g01433.t1
5Nase10 (nv5Nase)	cytosolic 5 -nucleotidase	habu1_scaffold18 : 2268164 ... 2279432 : -	habu1_s18_g00174	habu1_s18_g00174.t1
5Nase11 (nv5Nase)	cytosolic 5 -nucleotidase 1b	habu1_scaffold2849 : 462585 ... 469949 : +	habu1_s2849_g10145	habu1_s2849_g10145.t1

(b)

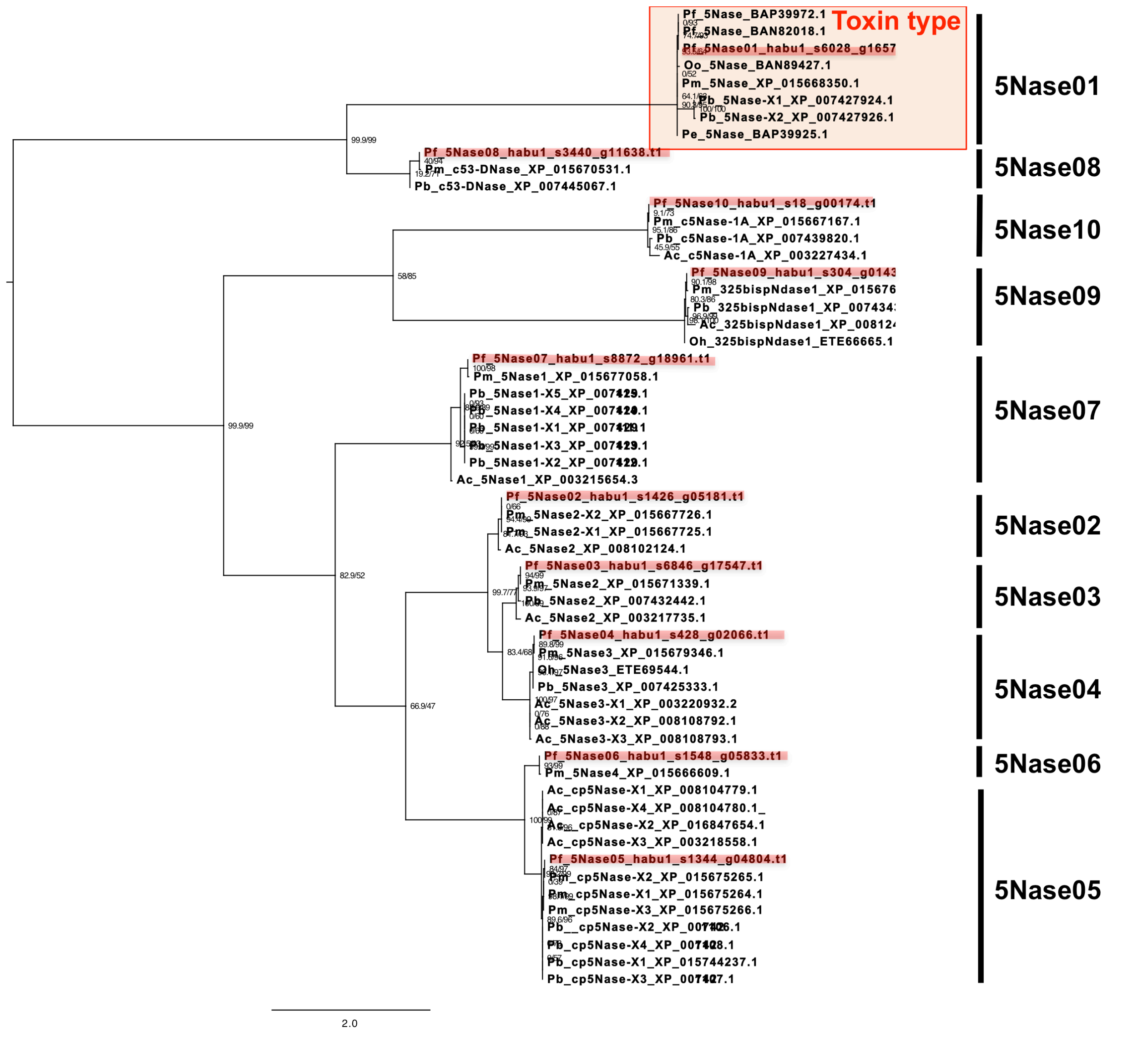

Supplementary Figure S17.

(a)

Gene	Name	Locus	Gene Model ID	Transcripts
DPP01 (nvDPP)	DPP-I	habu1_scaffold1219 : 174906 ... 207609 : +	habu1_s1219_g04195	habu1_s1219_g04195.t1
DPP02 (nvDPP)	DPP-II	habu1_scaffold998 : 329636 ... 331823 : +	habu1_s998_g03532	habu1_s998_g03532.t1
DPP03 (nvDPP)	DPP-II	habu1_scaffold998 : 322306 ... 326986 : +	habu1_s998_g03530	habu1_s998_g03530.t1
DPP04 (nvDPP)	DPP-III	habu1_scaffold7877 : 9596 ... 37295 : +	habu1_s7877_g18328	habu1_s7877_g18328.t1
DPP05 (svDPP)	svDPP-IV	habu1_scaffold1020 : 1996805 ... 2061742 : +	habu1_s1020_g03622	pb016480_c171896_f1p3_4438
DPP06 (nvDPP)	DPP-VIII	habu1_scaffold1390 : 679600 ... 693217 : -	habu1_s1390_g04966	habu1_s1390_g04966.t1
DPP07 (nvDPP)	DPP-IX	habu1_scaffold5905 : 38286 ... 91928 : +	habu1_s5905_g16391	habu1_s5905_g16391.t1
DPP08 (nvDPP)	DPP-X	habu1_scaffold13003 : 203276 ... 412685 : -	habu1_s13003_g20945	habu1_s13003_g20945.t1

(b)

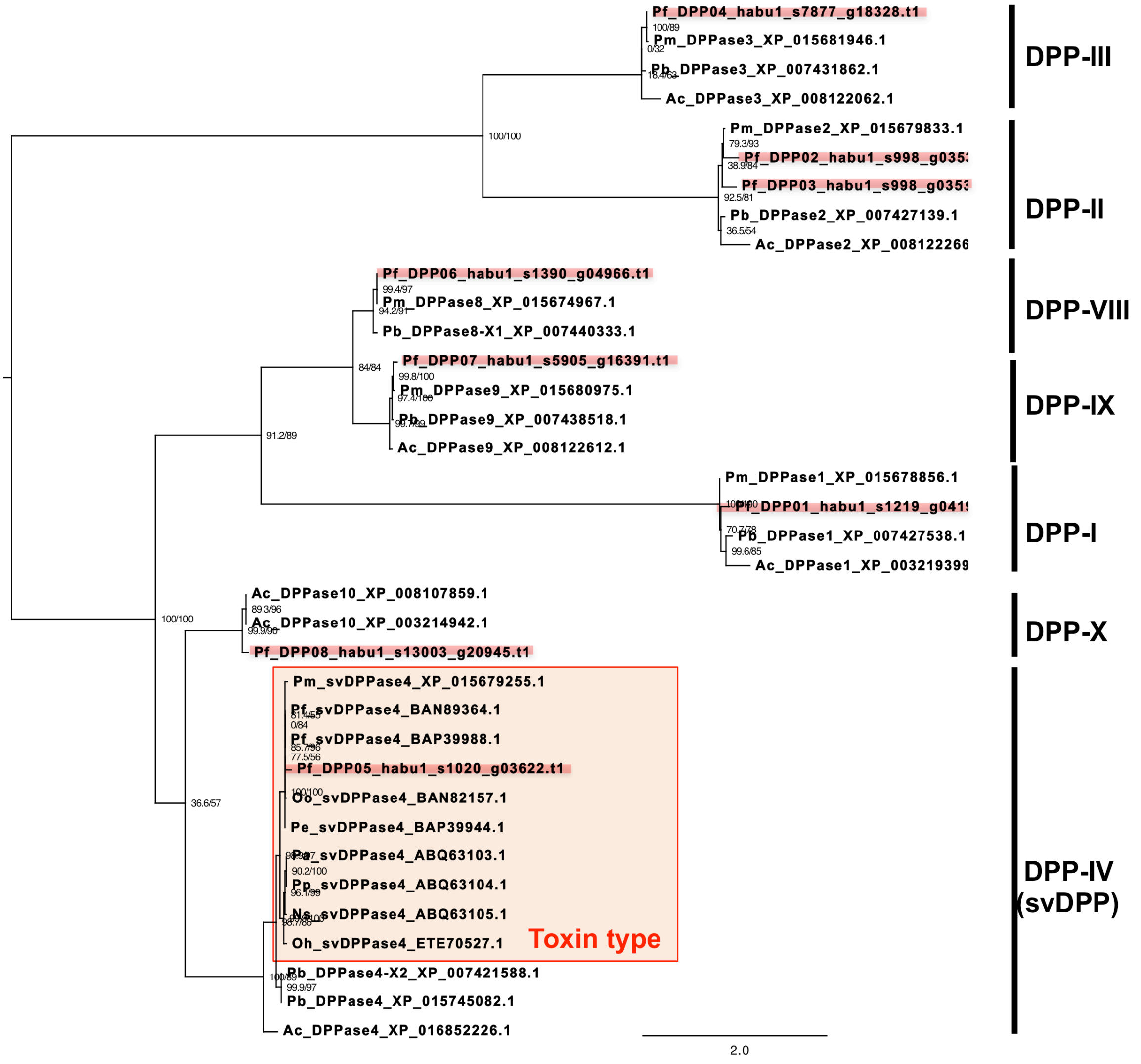

Supplementary Figure S18

(a)

Gene	Name	Locus	Gene model ID	Transcripts
svVEGF01	svVEGF(svVEGF_F)	habu1_scaffold565 : 505831 ... 554056 : -	habu1_s565_g02679	pb016481_c76319_f71p22_1316
nvVEGF01	VEGF-A	habu1_scaffold6836 : 129006 ... 390238 : +	habu1_s6836_g17529	habu1_s6836_g17529.t1
nvVEGF02	VEGF-C	habu1_scaffold9381 : 115236 ... 156444 : +	habu1_s9381_g19343	habu1_s9381_g19343.t1

(b)

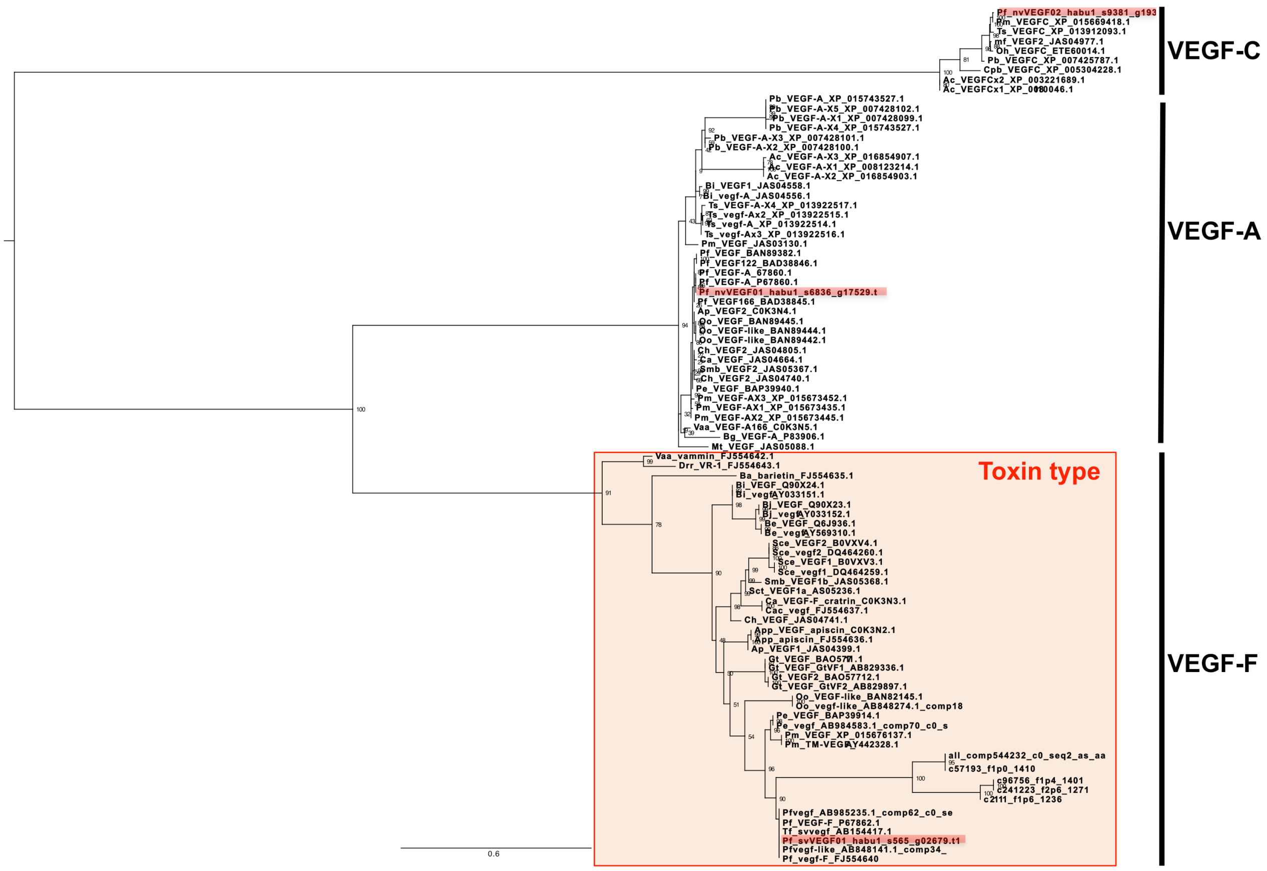

Supplementary Figure S19

(a)

Gene	Name	Locus	Gene Model ID/Annotation Link	Transcripts
PDE_01	PDE_01(svPDE)	habu1_scaffold149 : 1524169 ... 1594790 : +	habu1_s149_g00804	pb016481_c221783_f35p18_2633
PDE_02	PDE_02 (nvPDE)	habu1_scaffold149 : 1600729 ... 1649852 : +	habu1_s149_g00805	habu1_s149_g00805.t1
PDE_03	PDE_03(nvPDE/cAMP)	habu1_scaffold149 : 3362511 ... 3486620 : +	habu1_s149_g00834	habu1_s149_g00834.t1

(b)

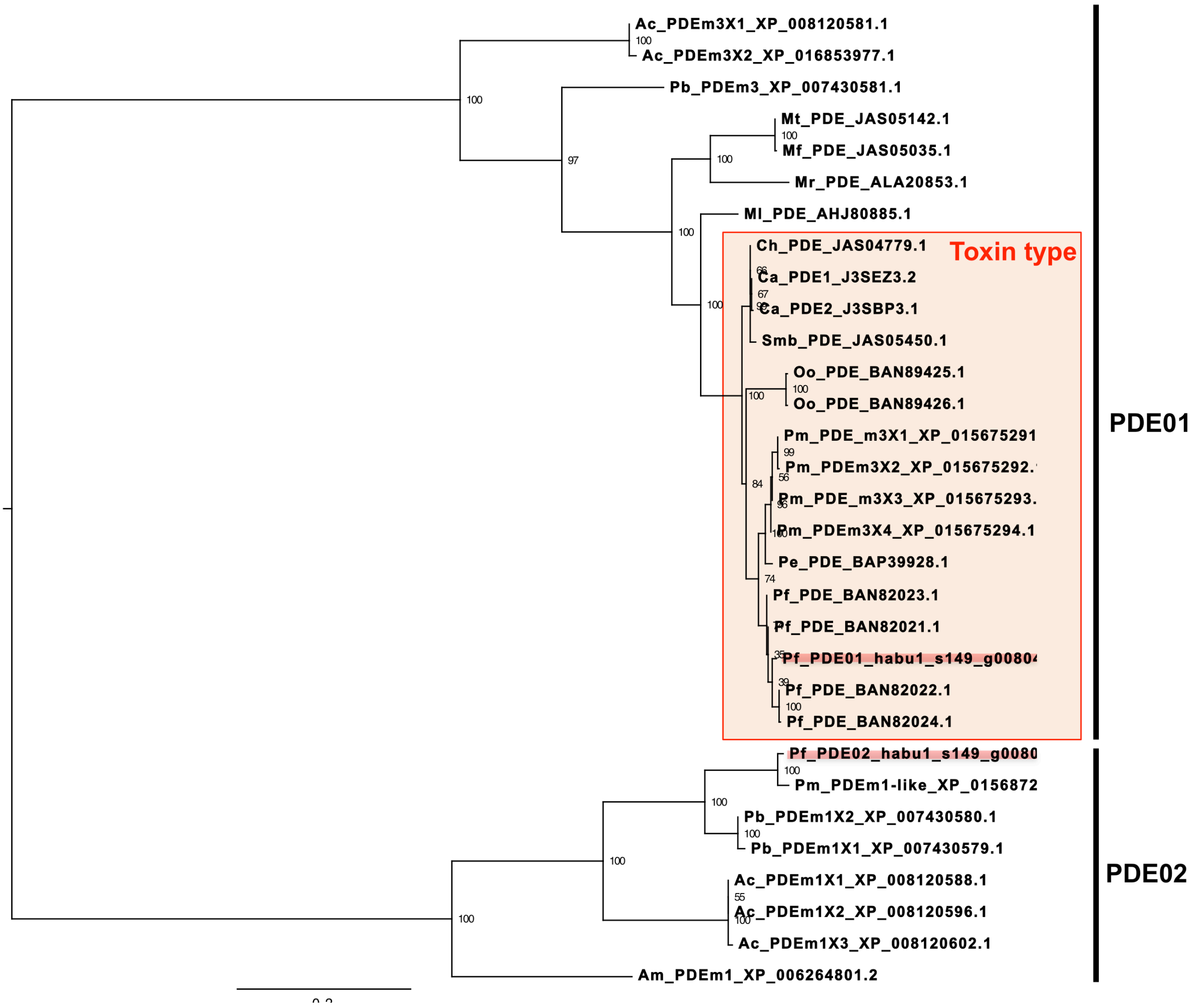

Supplementary Figure S20

(a)

Gene	Name	Locus	Gene Model ID	Transcripts
PLB01 (svPLB)	phospholipase b	habu1_scaffold1233 : 1498267 ... 1531920 : +	habu1_s1233_g04284	pb016481_c662_f13p9_1819
PLB02	phospholipase b-like 2	habu1_scaffold1964 : 35979 ... 45965 : -	habu1_s1964_g07200	habu1_s1964_g07200.t1
PLB03	phospholipase b1(membrane-associated)	habu1_scaffold313 : 1861453 ... 1973632 : -	habu1_s313_g01520	habu1_s313_g01520.t1
PLB04	60 kDa lysophospholipase	habu1_scaffold3352 : 459066 ... 534431 : -	habu1_s3352_g11424	habu1_s3352_g11424.t1
PLB05	lysophospholipase-like	habu1_scaffold303 : 156916 ... 176572 : +	habu1_s303_g01427	habu1_s303_g01427.t1

(b)

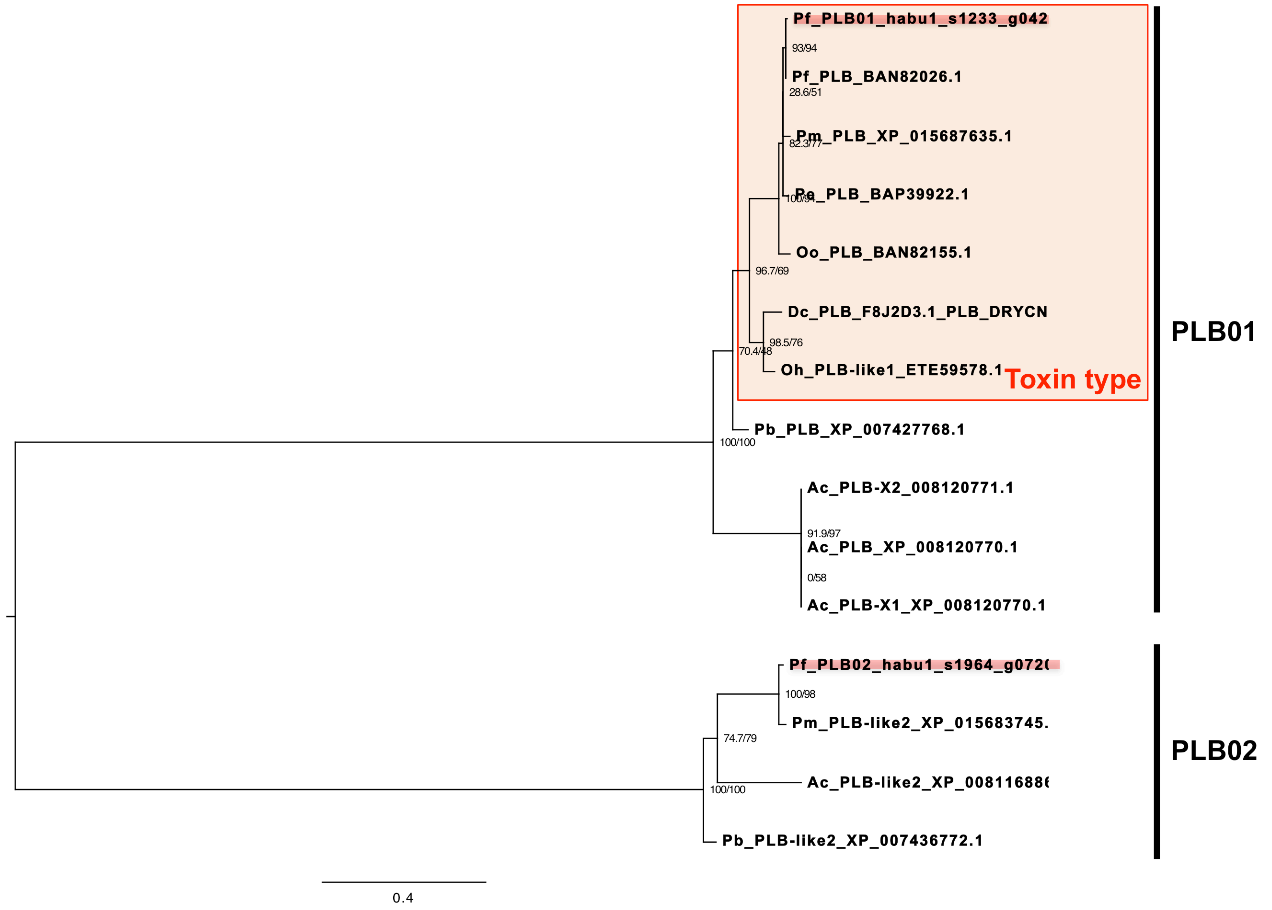

Supplementary Figure S21

(a)

Gene	Name	Locus	Gene model ID	Transcripts
CNP01	BPP_CNP (svCNP)	habu1_scaffold258676: 1...337: -<br>habu1_scaffold4348 : 15402 ... 16141 : -	habu1_s258676_g24318	habu1_s258676_g24318.t1
CNP02	B-type natriuretic peptides A-like	habu1_scaffold20540 : 7387 ... 10895 : -	habu1_s20540_g22311	habu1_s20540_g22311.t1

(b)

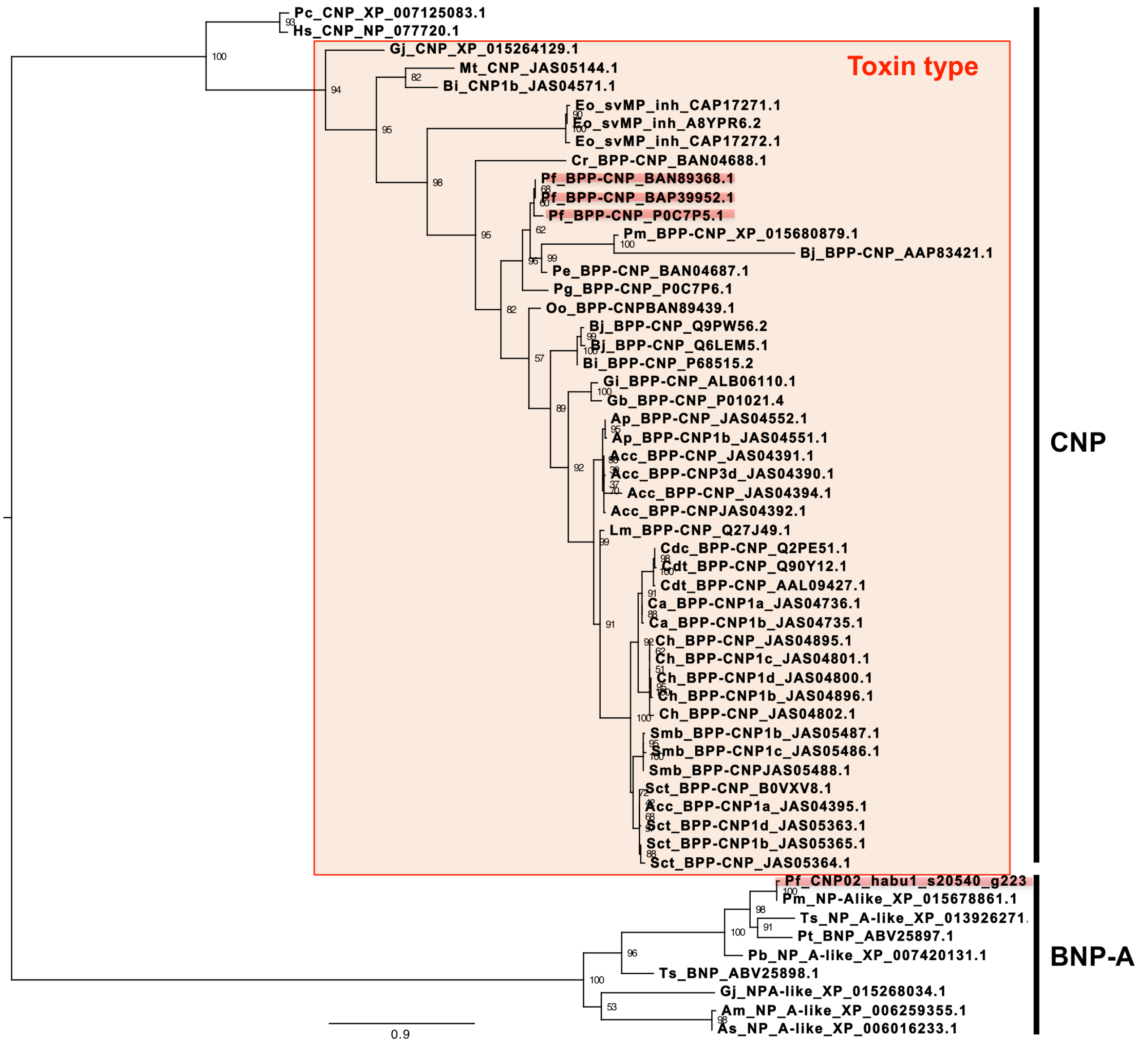

Supplementary Figure S22.

(a)

Gene	Name	Locus	Gene model ID	Transcripts
GPCase01 (svGPCase)	glutaminyl-peptide cyclotransferases	habu1_scaffold510 : 291600 ... 305425 : -	habu1_s510_g02384	pb016481_c398311_f1p1_2071
GPCase02 (nvGPCase)	glutaminyl-peptide cyclotransferases	habu1_scaffold3067 : 69888 ... 75755 : -	habu1_s3067_g10859	habu1_s3067_g10859.t1

(b)

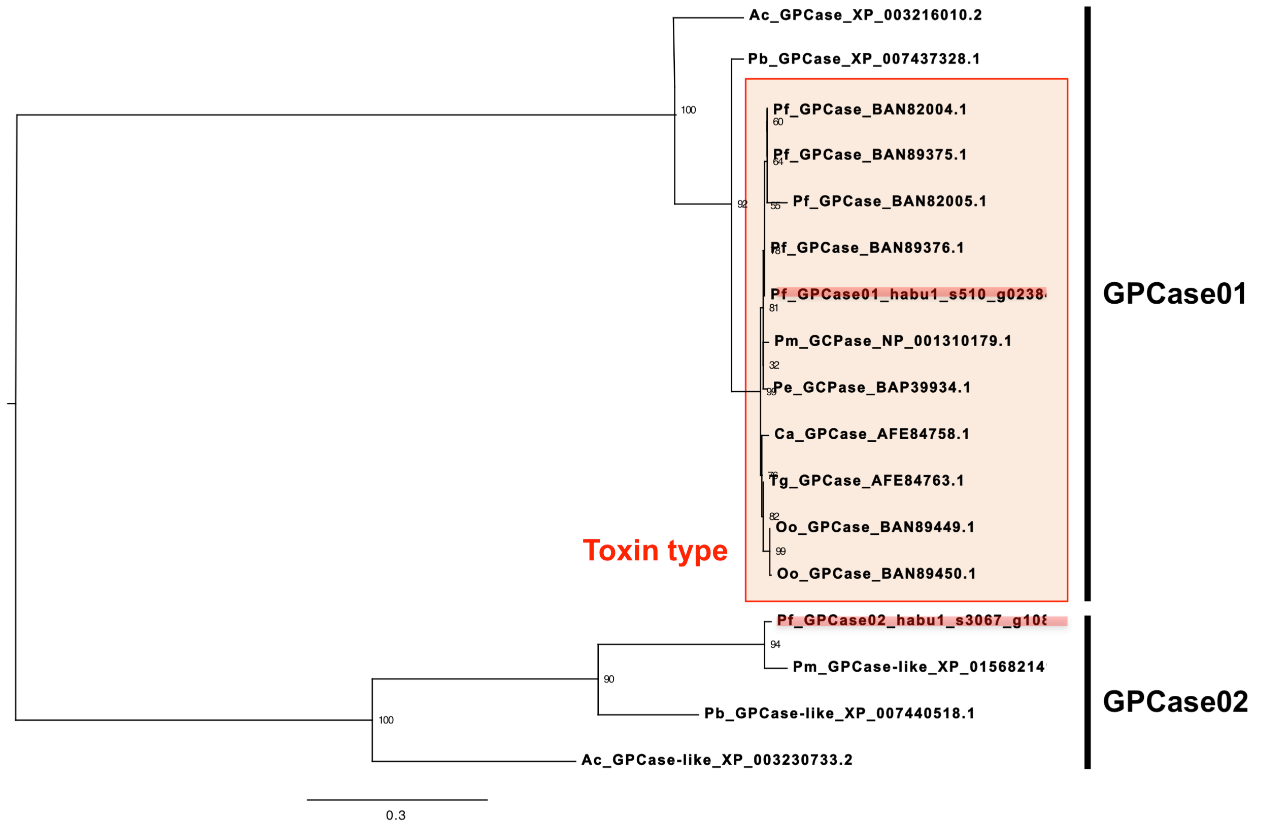

Supplement: Supplementary file 1 — Supplementary Information [file 41598_2018_28749_MOESM1_ESM.pdf]
